# The Northern Route for Human dispersal in Central and Northeast Asia: New evidence from the site of Tolbor-16, Mongolia

**DOI:** 10.1038/s41598-019-47972-1

**Published:** 2019-08-13

**Authors:** Nicolas Zwyns, Cleantha H. Paine, Bolorbat Tsedendorj, Sahra Talamo, Kathryn E. Fitzsimmons, Angaragdulguun Gantumur, Lkhundev Guunii, Odsuren Davakhuu, Damien Flas, Tamara Dogandžić, Nina Doerschner, Frido Welker, J. Christopher Gillam, Joshua B. Noyer, Roshanne S. Bakhtiary, Aurora F. Allshouse, Kevin N. Smith, Arina M. Khatsenovich, Evgeny P. Rybin, Gunchinsuren Byambaa, Jean-Jacques Hublin

**Affiliations:** 1 Department of Anthropology, University of California, 95616 Davis, USA; 2 Department of Human Evolution, Max-Planck Institute for Evolutionary Anthropology, 04103 Leipzig, Germany; 3 Center for Experimental Archaeology in Davis, University of California, 95616 Davis, USA; 4 Department of Archaeology, University of Cambridge, Cambridge, UK; 5 Institute for History and Archeology, Mongolian Academy of Sciences, Ulaanbaatar, Mongolia; 6 Research Group for Terrestrial Palaeoclimates, Max Planck Institute for Chemistry, Hahn-Meitner-Weg 1, 55128 Mainz, Germany; 7 Service de Préhistoire, Université de Liège, 4000 Liège, Belgium; 8 Laboratoire TRACES UMR 5608, Université Toulouse - Jean Jaurès, Toulouse, France; 9 Department of Anthropology, University of Pennsylvania, Philadelphia, USA; 10 Section for Evolutionary Genomics, the GLOBE Institute, University of Copenhagen, Copenhagen, Denmark; 11 Winthrop University, Rock Hill, SC 29733, USA; 12 Department of Anthropology, Harvard University, Cambridge, USA; 13 Institute of Archaeology and Ethnography of the Siberian Branch, Russian Academy of Sciences, Novosibirsk, Russian Federation; 14 Archaeology Institute, University of the Highlands and Islands, Kirkwall, UK; 15 Department of Chemistry “G. Ciamician”, University of Bologna, Via Selmi, 2, 40126 Bologna, Italy

**Keywords:** Archaeology, Archaeology

## Abstract

The fossil record suggests that at least two major human dispersals occurred across the Eurasian steppe during the Late Pleistocene. Neanderthals and Modern Humans moved eastward into Central Asia, a region intermittently occupied by the enigmatic Denisovans. Genetic data indicates that the Denisovans interbred with Neanderthals near the Altai Mountains (South Siberia) but where and when they met *H. sapiens* is yet to be determined. Here we present archaeological evidence that document the timing and environmental context of a third long-distance population movement in Central Asia, during a temperate climatic event around 45,000 years ago. The early occurrence of the Initial Upper Palaeolithic, a techno-complex whose sudden appearance coincides with the first occurrence of *H. sapiens* in the Eurasian steppes, establishes an essential archaeological link between the Siberian Altai and Northwestern China . Such connection between regions provides empirical ground to discuss contacts between local and exogenous populations in Central and Northeast Asia during the Late Pleistocene.

## Introduction

Although models for *H. sapiens’* early dispersals out of Africa emphasize a southern route to Asia^1–5^, Neanderthal and Modern Human (MH) fossils in Siberia^6–9^ suggest that at least two other dispersals took place across the Eurasian steppe north of the Asian high mountains. Given the size of the area considered, human fossils are few but recent studies have suggested that a major change in the regional archaeological record could be indicative of a large-scale human dispersal event. Known as the Initial Upper Palaeolithic (IUP), it refers to the sudden appearance in contiguous regions of a specific blade technology sometimes associated with bone tools and ornaments^10–17^. How old these assemblages are, and how long the phenomenon lasts are still controversial questions, and little is known about the timing and environmental context of these population movements. Here we present new data following excavations of an archaeological site located in a low altitude pass, the *Ikh-Tolborin-Gol*, which connects Siberia with northern Mongolia. Our results document the early occurrence of the Upper Paleolithic in Mongolia and provide a chronological reference for population movement across Northeast Asia during the Late Pleistocene.

## Site Description

**Figure 1:**
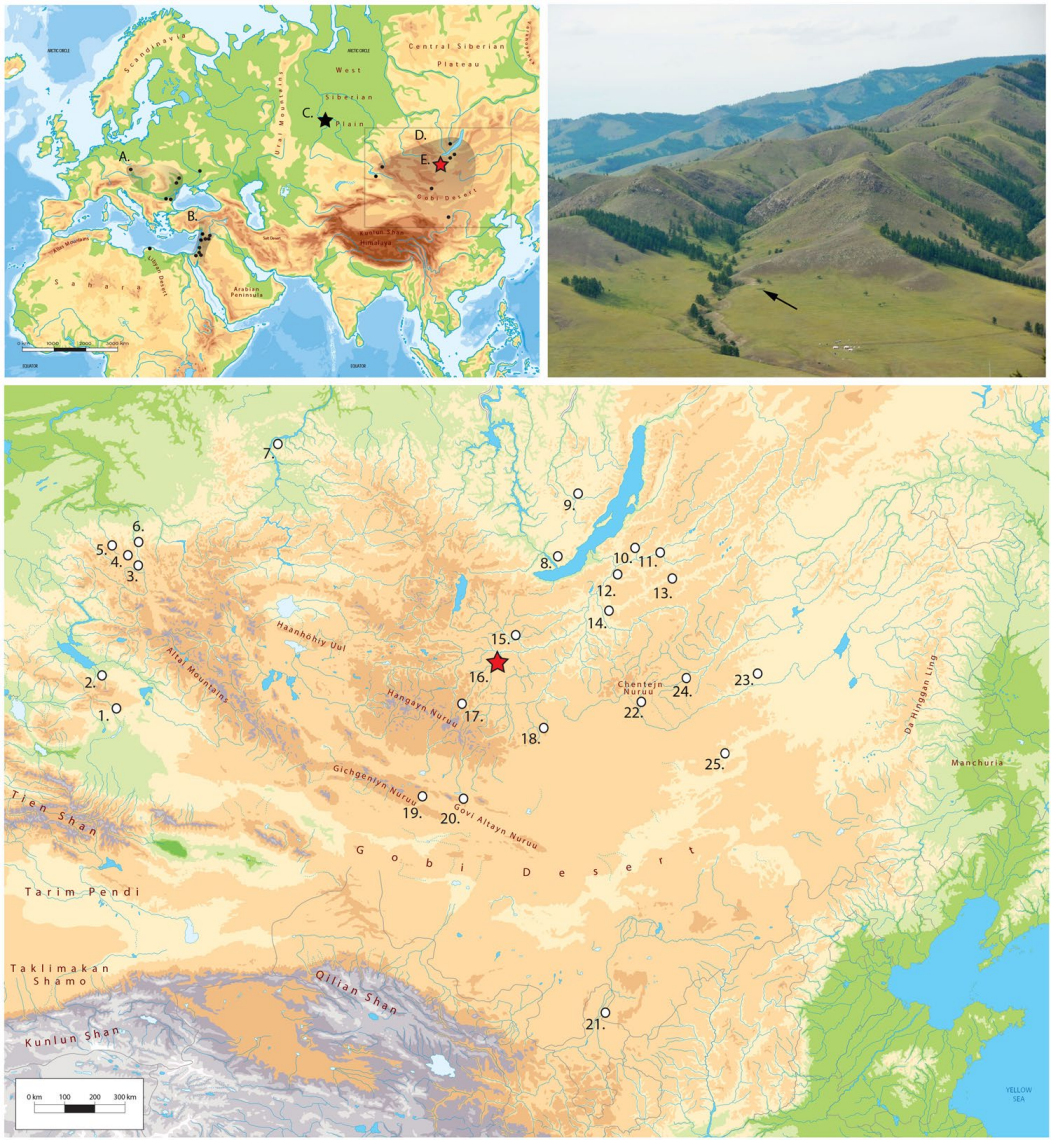
*Above (right)*: Schematic map of the main IUP sites in Central Europe (Bohunician) (**A**), in the Middle-East (Emirean) (**B**) and in Asia (**D**); and the Ust-Ishim human femur (**C**). *Above (left):* View on the T16 site and the Western flank of Tolbor Valley. *Below*: Detailed map of the potential IUP sites in the region. 1, Luotuoshi; 2, Ush-Bulak; 3, Malo Yaloman, Cave; 4, Kara-Bom, 5, Ust-Karakol-1 and Denisova Cave; 6, Kara-Tenesh; 7, Derbina sites; 8, Arembovski; 9, Makarovo-IV; 10, Khotyk; 11, Barun-Alan sites; 12, Kamenka and Varvarina Gora; 13, Tolbaga; 14, Podzvonkaya; 15, Egiin-Gol sites (Dörölj 1–2); 16, Tolbor-16 and Tolbor-4; 17, Tsatsyn Ereg; 18, Mojlt’yn-Am; 19, Chiken sites; 20, Tsagan-Agui; 21, Shuiddonggou 1; 22, Khanzat-1; 23, Khavsgayt (and Salkhit); 24, Rashaan Khad; 25, Otson Tsokhio^16–18,75–77^; Geo-atlas background map).

The Tolbor-16 (N49 13.619 E102 55.383) (T16 hereafter) site is located in the Northern Hangai Mountains, along the western flank of the Tolbor River valley (1169 m asl), 13 km south of the confluence with the Selenga River^18^ (Fig. 1). From 2011 to 2016, we excavated five pits for archaeological and geoarchaeological investigations (SI Section 1). Pits 1 and 4 are the largest excavated areas, whereas Pits 2, 3 and 5 are smaller trenches that provide stratigraphic and chronological control. Six main archaeological horizons dating from the MIS3 to the Holocene have been documented at the site. The material relevant to the earliest human dispersals derives from the lowermost archeological horizon (AH6) in Pits 1 and 4.

### Stratigraphy

The T16 sediments consist of loess, reworked loess and soliflucted laminar silt, with gravel and cobbles. The sites sit at the mouth of a small canyon, which seems to have been a source for the sediment that accumulated through a complex interplay of colluviation, eolian sedimentation, and solifluction. Sedimentological data indicate a low-energy depositional environment (see SI Section 2). In most of the exposed sections at T16, the stratigraphy divides into three main units: the Holocene soil complex (unit 1) comprising one or more chernozem or kastanozem type soils (FAO classification system), an underlying layer of loess and reworked loess (unit 2), and a soliflucted diamict of laminar silt with gravel and cobbles (unit 3), which extends to a depth of 2.5 m. In Pit 4, at least five superposed solifluction lobes were identified within unit 3 (sub-units 3a, 3b, 3c, and 3d); archaeological material is restricted to the upper 2 m of the section and is present in units 1 and 2 and in solifluction lobes 3a, b and c.

**Figure 2:**
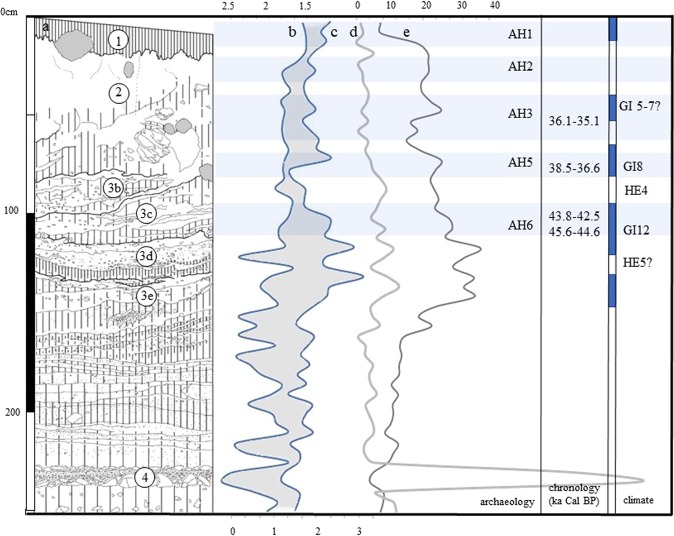
From left to right: (**a**) North stratigraphic section at T16 Pit 4, showing the three main stratigraphic units and solifluction lobes 3b-e (3a was restricted to the westernmost part of the section and was not sampled); (**b**) Standard deviation of the fine (<2 mm) fraction by laser particle size analysis (top axis); (**c**) Organic matter content relative to mineral matter by loss-on-ignition (%, bottom axis); (**d**) Gravel content (wt%, top axis); (**e**) Calcium carbonate content relative to mineral matter by loss-on-ignition (%, hollow dots, top axis) within the sediments at pit 4. Well-sorted primary loess has a low standard deviation; redeposition and soil development affect this (see SI Section 2); the distance between (**b,c**) (gray area) is therefore a rough proxy for climate, with climatic amelioration indicated where the distance is greater. Gravel is present in the sampled sediments through gravitational input; the proportion of gravel increases during prolonged surface exposure or slow sediment accumulation. Carbonate content increases when evaporation is high relative to precipitation. Archaeological horizons (AH), modelled age of the boundaries between events (SI Section 4), and climatic interpretation using NGRIP as a comparison are also indicated.

Six archaeological horizons (AH) were identified within the three stratigraphic units at Pit 4 (Fig. 2). The soil complex contains a few artefacts, and these are designated AH1. AH2 occurs within primary loess at the top of unit 2, and AH3, in reworked sediments lower within unit 2, consists of intrusive material from the underlying unit (probably AH4-AH6). Slope change – resulting in the truncation of unit 3 and its partial incorporation by solifluction into unit 2 – is responsible for the mixed nature of AH3. AH4 and AH5 sit within lobes 3a and 3b, and AH6 - with which this paper is primarily concerned - lies at the bottom of the sequence in unit 3 c. A similar succession occurs in Pit 1 (c. 4 m to the northeast), although it also contains additional sedimentary units (SI Section 2) not preserved in pit 4.

### Chronology

We used three geochronological methods (polymineral post-IR IRSL, Quartz OSL and radiocarbon) to determine the antiquity of the site. For luminescence, we ran paired samples collected from Pits 1, 2 and 3 to allow an overarching chronological framework for sediment deposition (SI Section 3). Measurements obtained on both quartz and polymineral (feldspar-dominated) fine-grained aliquots provide independent age control; with one exception (EVA-1438), all paired ages fall within 2-sigma error. For radiocarbon, we collected bone samples from cultural layers AH2 through AH5 in Pits 1 and 4 (SI Section 4) to obtain radiocarbon dates on the animal species associated with the archaeological deposits. Due to the bone preservation, cut-marks, tool use or manufacture are not identifiable, but the spatial distribution of bones tightly coincides with the archaeological occupations. Deposits without stone tools contain few to no bones.

The results indicate that the T16 sequence dates to the Late Pleistocene, spanning Marine oxygen Isotope Stage (MIS) 3 and MIS2. AH2 is constrained to MIS2; AH3 and AH5 dates between to 35.1–38.5 ka. The material corresponding to the earliest human occupation is associated with AH6 and dated in Pit 1 and Pit 4 to 42.5–45.6 ka. Stratigraphically, AH3 and AH6 provide a temporal bracket for AH4, an assemblage located in a disturbed deposit that probably includes material of different ages. Although the archaeological sequence is concentrated in the two first meters of deposits, the age of the laminar sediment accumulation below AH6 is still to be determined.

### Palaeoenvironmental conditions

The deposition of the 2.5 vertical meters of laminar silt in pit 4 (the first meter hosting AH 4, 5, and 6) is the result of episodic loess deposition, slopewash, carbonate crust formation, and pedogenesis. It records a different precipitation regime than today, with cyclic – both seasonal and millennial-scale – variation in moisture availability (see SI Section 2). Periodic climatic downturns during the deposition of unit 3 result in episodes of solifluction, confirming that northern Mongolia witnessed multiple fluctuations in moisture and temperature during MIS3^19,20^. The high organic matter content of sediments associated with AH6 is consistent with a period of milder, relatively moist climate at the time of the occupation(s). A climatic deterioration follows the deposition of AH6; an episode of solifluction, which takes place together with a reduction in colluviation, the formation of a thick carbonate crust in Pit 1, and a possible episode of loess accumulation in Pit 4, is evidence for a period of cold and aridity. The overlying soliflucted slopewash deposits contain a lithic assemblage (AH5) of different character.

The deposition of the laminar sediment coincides with a period for which regional ecological (and other proxy) records indicate relatively moist conditions^19–25^; the dominance of colluviation over eolian deposition in much of the section supports moist conditions. At the same time, the cyclic development of thin carbonate crusts, normally an indicator of aridity, becomes important before and during the deposition of the IUP sediments (sub-units 3e, d, c); this may relate to strong seasonality of either insolation^21^ or precipitation patterns. Abundant root cell calcite throughout the studied section indicates the presence of grass, and, although reworked, strongly humic and calcareous crumbs within the redeposited material are consistent with steppe vegetation; episodic decreases in the mean particle size of the silt size fraction within the reworked sediments (SI Section 2) may relate to denser vegetation cover at certain times during the hypothesized warm events recorded in sub-units 3e, d, and c and a concomitant reduction in the energy of reworking. The transition to eolian deposition is difficult to date owing to truncation, but ^14^C dates from Pits 1 and 4 suggest a shift before the MIS3-2 transition; the timing of this change, while coinciding with global cooling and aridification, links the section to climate records from the south and east (eg Salawusu^26^, Guliya ice core^27^, Hulu cave^28^) where pronounced cooling is recorded from an earlier date than in the North Atlantic, and to regional climate records, which suggest a change to arid conditions at this time^19,20,23–25^.

In Pit 1 and Pit 4, bones occur in low concentrations within all AHs. They are visibly more numerous at the top of the laminated sediments of unit 3, within AH5 and AH6. By contrast, we found no bones associated with the archaeologically sterile sediments below (SI Section 4). The degree of fragmentation combined with surface alteration is a significant obstacle to the identification of taxa or anthropogenic modifications. Zooarchaeology by Mass Spectrometry (ZooMS)^29,30^ screening is in progress to identify species on bones from Pit 4 (SI Section 6) while preliminary results agree with previous zooarchaeological identifications^18^. They indicate the occurrence of *Bos* sp., Caprinae sp. and *Equus* sp., Felid sp. and Elephantidea sp. *Bos* sp. is associated with human occupation in solifluction lobes 3a trough 3c for which three different age boundaries are estimated using radiocarbon dates and Bayesian modelling: 35.1–38.5 ka; 36.6–42.5 ka and 42.5–45.6 (dating the AHs described above) (SI Section 4). Most of these taxa are common in an Pleistocene open steppe or a taiga environment but they also occur northward, in sites where tundra environments predominate (e.g. Transbaikal)^31^. According to a pollen record from the region, steppe and taiga environments alternate during the cold and warm climate fluctuations in the MIS3^25^.

### Lithic assemblage from AH6

The oldest archaeological assemblage, AH6, occurs within stratigraphic Unit 3c in Pit1 and in Pit4. The assemblage described here is a sample selected for its stratigraphic integrity and its clear association with the chronological data (SI Section 5). It contains 826 lithic artifacts with 362 piece-plotted artifacts (>20 mm) such as 19 cores and preforms, and 287 blanks, of which 91 are retouched tools. It also includes 463 smaller fragments (<20 mm). The artifacts are made of cryptocrystalline materials of medium to fine grain. The origin of the raw material is mostly local and it outcrops throughout the valley in sub-vertical bands.

**Figure 3:**
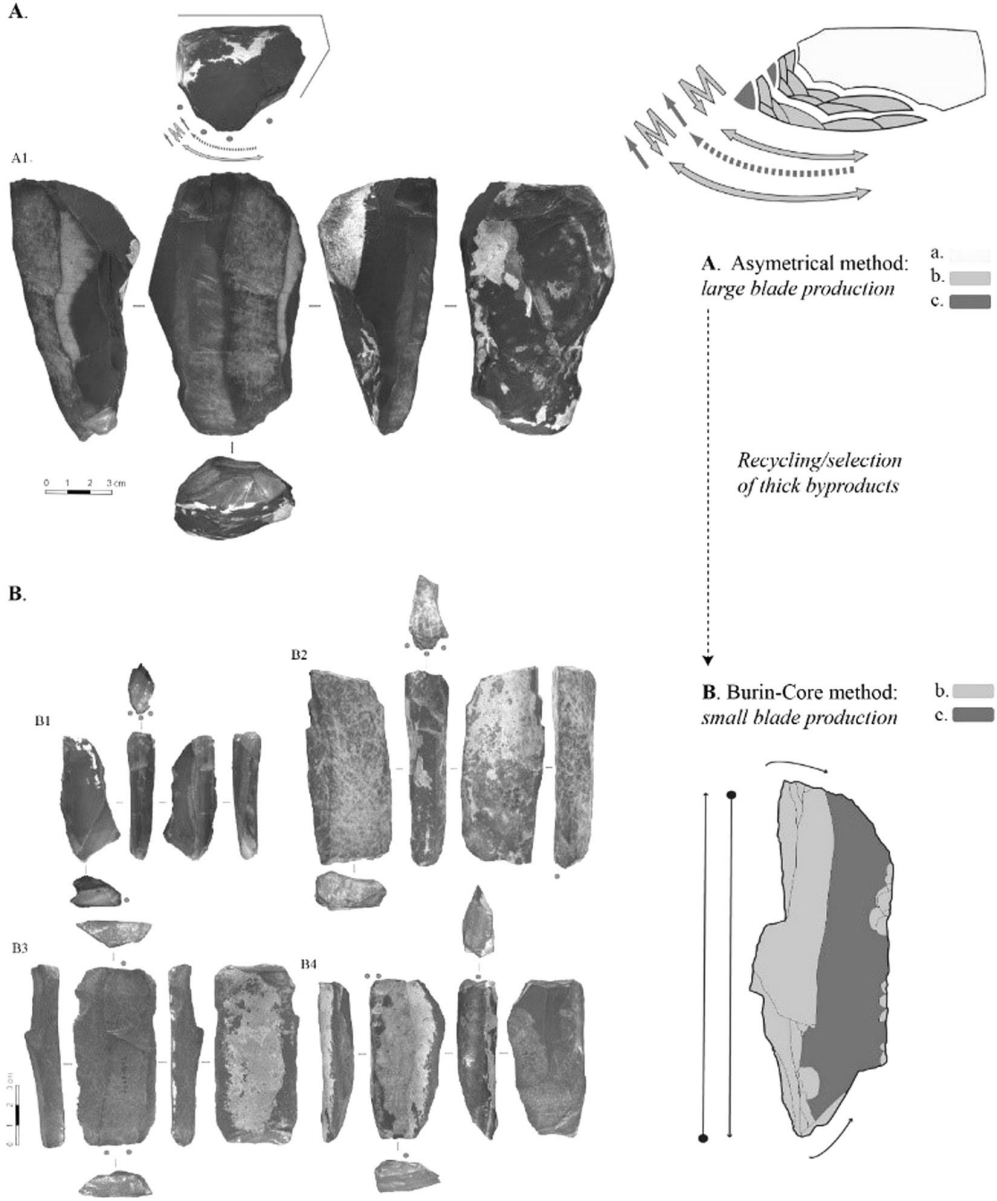
Reduction sequence model for the blade production in AH6. Asymmetrical reduction (**A**) produces large blades (Ab) (SI5) used as tools and thick technical blades (Ac); some of the thickest blades are turned into cores to produce small blades/bladelets using the burin-core (B1, B2; B3) or the truncated-facetted methods (B3).

The assemblage mainly consists in blades and artifacts linked with their production. Two specific patterns of core reduction dominate the sample. A method known elsewhere as asymmetrical (or oblique) core reduction^14^ leads to the production of large (and sometimes massive) blades, detached from two opposed platforms. The core reduction is initiated by the production of a crest while the frequent maintenance is documented by the removal of neo-crest and side (*debordant*) blades (Fig. 3A). The striking platform is regularly reshaped by the removal of core tablets. While some of the large blades are retouched into tools, the thickest ones are turned into cores using the burin-core method, to produce small blades/bladelets^32^ (Fig. 3B) (SI Section 5). The combination of the asymmetrical and the burin-core methods into an intricate system provides the basis for a solid technological definition of the IUP^14,17^, and specifically the Asian variant thereof.

As it is often the case with IUP, some features in the assemblage are reminiscent of the Middle Palaeolithic (MP) (SI Section 5). For instance, a few artifacts resemble Levallois cores but a close look at their size and geometry indicates that they are blade cores pushed to exhaustion. In the sample studied, the MP features seem to reflect the site function (expedient tools, exhausted cores), the technique (mineral hammer) or the method (e.g. *debordant* blade) in use during the blade production. Overall, all the specific features of the assemblage are perfectly in line with definition of the IUP in Asia^14,16^.

## Implications

The definition of the IUP in Central and Northeast Asia is mostly based on the detailed study of sites from the Gorny Altai (e.g. Kara-Bom)^10–17^ dated as early as 45–47 ka^32–34^. Eastward, where the Selenga River reaches Lake Baikal, a group of sites shows the same lithic technology (SI Section 5). At Kamenka, the IUP is potentially old but with problematic dating inconsistencies ranging 45.8 ka to 28.1 ka^35,36^. There are other sites between the Altai and Lake Baikal that probably belong to this technocomplex but for which confirmation is needed (e.g. Makarovo-V, dated to >39 ka uncal BP)^37^. In the same drainage system, T16 (along with Tolbor-4) yielded strong evidence for the occurrence of the IUP about 450 km upstream from Lake Baikal. With dates ranging between >41.5 to 31.5 ka uncal BP, the chronology is still uncertain^38–40^. The results presented here confirm that the IUP reaches Northeast Asia by 45 ka. We also illustrate geological processes (e.g. solifluction) that, if not identified, could generate artificial noise in the chronological signal (SI Sections 2 and 5).

The AH6 assemblage from T16 confirms that the IUP occurs in the Selenga River basin at roughly the same time it appears in the Altai. With such striking similarities between coeval assemblages, the most parsimonious way to explain such an archaeological consistency between contiguous regions is to consider the Asian IUP is a relatively united phenomenon – or techno-complex^14,41^ – that dispersed across Siberia to reach Mongolia around 45 ka^10–17,42^, moving south and east to reach Northwestern China^43–45^ perhaps as far as the Tibetan Plateau^46^.

**Figure 4:**
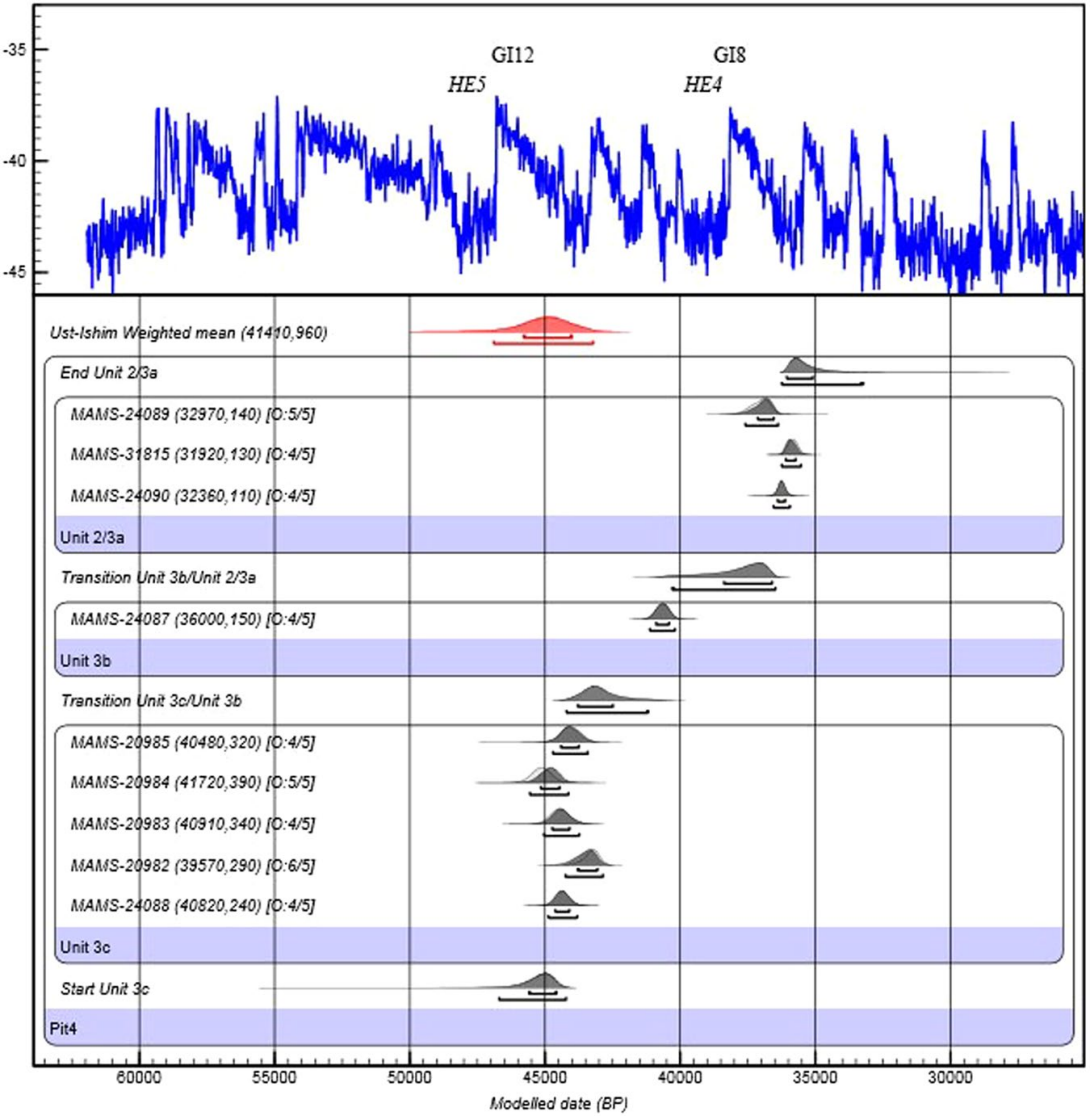
Bayesian model of T16 radiocarbon dates and the mean ^14^C date of Ust-Ishim *H. sapiens* femur. The IUP assemblage is associated with Unit 3c, and later UP assemblages are associated with Unit 3b and Unit 2/3a. The calibrated date of the earliest *H. sapiens* fossil in Northeast Asia, Ust-Ishim (Fig. 1) is in red. Dates are calibrated using IntCal13^78^; the model and boundaries were calculated using OxCal 4.3^79^ including a General t-type Outlier Model^80^. The results are aligned with the NGRIP δ18O climate record.

The ages associated with the IUP at T16 and the modelled age boundaries for Unit 3c correspond to a short-lived period of milder climatic conditions ca. 45 ka (Fig. 4). This temperate episode is known in the northern hemisphere marine record as Greenland Interstadial 12 (GI12)^47^. Its environmental impact is well-documented in the terrestrial record of the Eurasian steppe^48^ and following a climate crisis during which Neanderthal populations reduced significantly, GI12 is thought to have contributed to the repopulation of Europe^49^. It also coincides with the appearance of the Emireo-Bohunician^50,51^, a techno-complex known as the Western variant of IUP^17^ that would correspond to human expansions from the Levant into Central Europe^13,15^. Although the impact of GI12 on the landscape is more variable eastward, it may have had profound demographic impact in the region. During this time period, hunter-gatherers ventured into challenging environments such as the tundra of Western Siberia^8^, one of the world’s largest unbroken lowlands (below 100 m asl), or up to 72° latitude North, well within the Arctic Circle^52^.

Based on our chronological data, the particularly harsh climatic event which appears to conclude the IUP in the valley – evidenced by an episode of solifluction, with a reduction in overall sedimentation rates, patchy loess accumulation, and the formation of a thick calcium carbonate crust in Pit 1 (SI Section 2) – is tentatively correlated with Heinrich Event 4 (HE4). Originally identified in the Atlantic Ocean^53^, the HE4 event (ca. 40 ka) is well documented in Asia^28,54^. In fragile eco-systems such as that at T16, where the long-term accumulation of sheet erosion deposits suggests sparser vegetation cover outside of climatic optima, millennial-scale cold events such as HE4 leading to changes in annual temperature or precipitation may accelerate catastrophic changes in vegetation and carrying capacity^55^. The position of the Tolbor sites at the transition zone between westerlies-dominated Siberia and monsoon-dominated southern- and eastern Inner Asia (and within the present-day core of the Siberian high which drives the Asian winter monsoon) makes them vulnerable to the complex interplay of several linked atmospheric systems in the past. Hence, the sedimentary record at T16 can be connected to both North Atlantic and East Asian climate records. The IUP here coincides with a period of warmer, moister, and probably more strongly seasonal conditions, which possibly changed the carrying capacity of the landscape and enabled human expansion through the area. The subsequent cold arid event appears to see off the IUP populations, suggesting that both global and regionally specific fluctuations in temperature and moisture collectively drove presence and absence here.

In the absence of a direct association between archaeological assemblages and identifiable human remains, at least two main hypotheses must be formulated. The first posits that the makers of the IUP in Asia are early *H. sapiens*. The dates from T16 are identical to those obtained from the human femur from Ust-Ishim, currently the oldest known representative of our species in the Eurasian steppe. The latter indicate that a dispersal event took place during the GI12 (Fig. 4)^8^. AH6 is significantly older than the age estimates of 35 ka for the Salkhit skull from in east Mongolia^56^ (Fig. 1). We note that the latter is equivalent to the lower chronological boundary of AH3. Also, we find more support for this hypothesis in the archaeological record of the Selenga River Basin. Where preservation allows for it, the earliest examples of beads and bone tools known in the region are associated with the IUP^16,57^. These objects have been almost exclusively associated with our species while they keep being produced in later phases of the Upper Paleolithic and beyond^18,57–59^.

The alternative hypothesis is that the IUP makers were local populations other than *H. sapiens*. Archaeological, fossil and genetic data suggest that Neanderthals crossed Central Asia several times^6,7,9^, potentially following the same inland route as *H. sapiens* up to the Altai. To date, there are no identifiable Neanderthal remains east of the Northwest Altai and there are simply no other human taxa securely dated to the GI12 in Siberia, Mongolia or Northwestern China. A potential chronological overlap is not excluded^6^, but Neanderthal remains found in cave sites such as Okladnikov or Chagirskaya are associated with Mousterian assemblages distinct from the IUP^60^. Despite recent efforts, the archaeological associations between fossil taxa, dates and archaeological components of layer 11 in Denisova Cave are yet to be firmly established^7,61,62^. The timing, distribution and the archaeology of the hominins known as the Denisovans is even less clear. Genetic diversity between specimens from the eponymous site suggests a long but probably discontinuous occupation of the Altai^63^, including encounters with Neanderthals and episodes of gene flow between with the two populations^9^. Most of these events- and the only direct date available for Denisovan specimen - pre-date (and sometime greatly^64^) the first occurrence of IUP in the Altai. While gene flow events are documented between Denisovans and *H. sapiens*, it is notable that age estimates ca. 45 ka for an early encounter^65^ are consistent with the dates presented here for AH6 (Fig. 4).

To summarize, the data at hand suggests that *H. sapiens* are more likely to be the makers of the IUP. The sudden appearance of this technology in Mongolia takes place in GI12, a temperate phase contemporaneous with the expansion of *H. sapiens*, in Western and Northern Siberia. Other scenarios cannot be rejected but they are less parsimonious, and they simply lack convincing alternatives regarding the archaeology of *H. sapiens* populations. Either way, the population movements illustrated by the dispersal of the IUP occur in a general context of climatic instability associated with MIS3 in Mongolia, and more generally in Central and Northeast Asia^66^. Such variations in the higher latitude of the Eurasian steppe imply different challenges (eg. continental climate, aridity) than those met along the southern route into the Indian subcontinent^67^. An inland route to Asia borne out by the archaeological and fossil record is, however, not exclusive of other dispersal models (e.g. Southern or ‘coastal’ Route) while it remains consistent with the available genetic data. The genome of an aboriginal Australian shows that deeply rooted populations have a shared ancestry coming from at least two early *H. sapiens* dispersals out of Africa into Asia^68^. Meanwhile, extant populations from continental East-Asia^69^ and in Melanesia^70,71^ show evidence of gene flow from Denisovan individuals from at least two gene flow events^65^. The timing might still be uncertain, but the dates presented here for the dispersal of IUP are consistent with the age estimates for an early *H. sapiens* – Denisovan encounter, distinct from the other gene flow events that would take place much later in Southeast Asia (or Melanesia)^65^. After subsequent mutations, the genes passed along from Denisovans around 45 ka may have been decisive for the survival of our species in extreme environments (Tibetan populations’ adaptation to hypoxia of high altitude)^72,73^. Finally, it is notable that East-Asian and Melanesian populations do also show evidence of gene flow coming from Neanderthal individuals^65^. Granted that the Pleistocene population dynamics were probably complex, a model integrating dispersals of several taxa, such as Neanderthal and *H. sapiens* across the continent is a parsimonious complement to a strict coastal migration scenario^74, 42^.

## Conclusions

The results obtained at T16 indicate that the IUP techno-complex occurs in Western front of East Asia as early as 45 ka. Contemporaneous with skeletal evidence of *H. sapiens*, the IUP in Tolbor establishes an essential archaeological link between Siberia and Northern China. Together with evidence from these adjacent regions, the material found in AH6 suggest that a widespread behavioral shift took place along the Eurasian steppe belt during a period of climatic instability. The latter included numerous milder and wetter phases that potentially increases the carrying capacity of the landscape, thereby facilitating human expansions in these regions. Although causality links remain to be established, the timing of the IUP and *H. sapiens* dispersal appears to be coeval with the millennial-scale GI12 temperate climate event.

## Method

We excavated the material presented here between 2011 and 2015 and analyzed it in 2016–2017. The emphasis is on the lowest archaeological assemblage (AH6) in Pit4 and Pit1. We used a total station to map the anthropogenic features but also to locate in place artifacts bigger than 2 cm in length. When possible, we recorded orientation and inclination of the artifacts. Within stratigraphic units, we excavated sediments by arbitrary spits of 3–5 cm thickness and dry-sieved using a 4- and 2-mm mesh. We recorded sample locations with the total station. Geological analyses include lithostratographic descriptions, analyses of bulk samples and micromorphology. In Pit 1 and Pit 4, we collected bulk samples every 5 cm (SI 2) from the ground surface and for micromorphology, sediment blocks at the interface between units or sub-units. More information can be found in the the Supplementary information.

## Supplementary information

Supplementary Info
