# Supplementary material for: The Northern Route for Human dispersal in Central and Northeast Asia: New evidence from the site of Tolbor-16, Mongolia
Source: Sci Rep. 2019 Aug 13;9:11759. doi: 10.1038/s41598-019-47972-1 (PMC6692324; doi:10.1038/s41598-019-47972-1)
Supplement: Supplementary file 1 — Supplementary Info [file 41598_2019_47972_MOESM1_ESM.pdf]

Supplementary Information for

The Northern Route for Human dispersal in Central and Northeast Asia: New evidence from the site of Tolbor-16, Mongolia

Nicolas Zwyns, Cleantha H. Paine, Bolorbat Tsedendorj, Sahra Talamo, Kathryn E. Fitzsimmons, Angaragdulguun Gantumur, Lkhundev Guunii, Odsuren Davakhuu, Damien Flas, Tamara Dogandzic, Nina Doerschner, Frido Welker, J. Christopher Gillam, Joshua B. Noyer, Roshanne S. Bakhtiary, Aurora F. Allshouse, Kevin N. Smith, Arina M. Khatsenovich, Evgeny P. Rybin, Gunchinsuren Byambaa, Jean-Jacques-Hublin

Nicolas Zwyns
Email: nzwyns@ucdavis.edu

This PDF file includes:

Supplementary text
Figs. S1 to S30
Tables S1 to 13
References for SI reference citations

1. **Excavation**

1.1. Site location

1.2. Excavated areas

1.3. Excavation method

2. **Sediment analyses**

2.1. Sampling

2.2. Laboratory methods

2.3. Description of the stratigraphy

2.4. Sedimentological analyses

2.5. Interpretations

3. **Luminescence dating**

3.1. Sample collection

3.2. Laboratory preparation and equivalent dose measurements

3.3. Luminescence characteristics

3.4. Dose rate calculation

3.5. Age calculations

4. **Radiocarbon dating**

4.1. Samples selection and location

4.2. Pre-treatment and dating

4.3. Modelled age estimates

5. **AH6 Lithic assemblage**

5.1. Spatial distribution and sampling procedures

5.2. Fabric analyses

5.3. Technological description

5.4. Relevance for the regional record

6. ZooMS

6.1. Sample composition

6.2. Results

1. Excavation

1.1. Site location

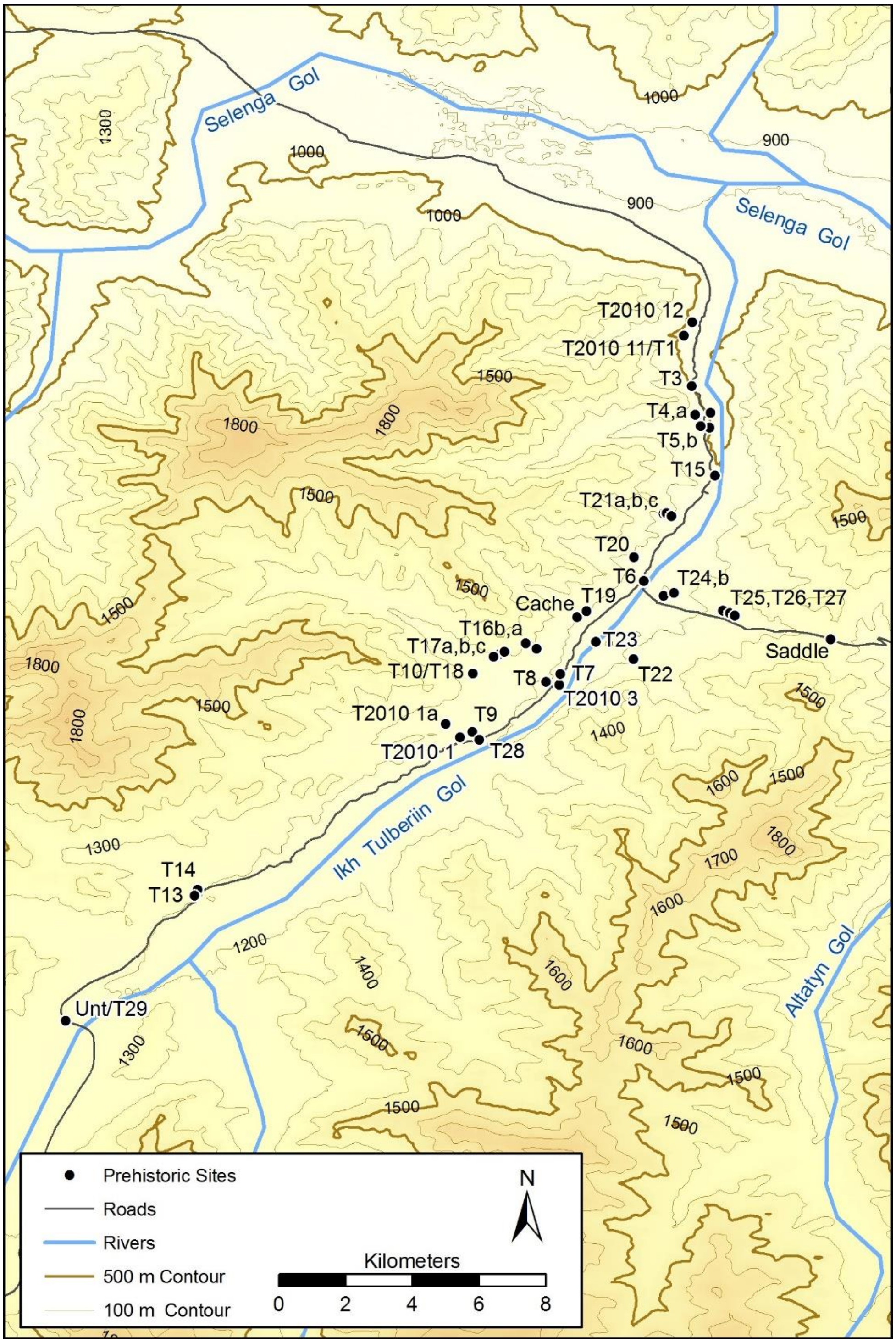

Figure S1: Distribution of the Upper Palaeolithic stone tool concentrations ('sites') in the Tolbor Valley. The main Pleistocene sequences are T4, T15, T16, T17 and T21

The Ikh-Tolborin-Gol is located approximately 300 km northwest of Ulaanbaatar, roughly 100km south of the Russian-Mongolian border. The Tolbor-16 (N 49° 13' 621"; E 102° 55' 381") (T16 hereafter) site is in the Northern Hangai, along the western flank of the Tolbor valley (1154 m asl). The site lies on the left bank of deep rill, at the junction between a rocky canyon coming down from the Shar Khad Mountain and the Valley open grassland (Fig. S2A, B, D). Currently, the rill activates during snowmelt and heavy rain. Its undulating trajectory is drawn by a succession of semi-circular eroded surfaces that exposed Pleistocene deposits and Palaeolithic stone artefacts. Gully incision truncates an early Holocene soil (the recent Holocene soil follows the gully slope), dating the current morphology of the stream bank to the Holocene. In the direct vicinity of the sites, the western slopes are covered by steppe grassland with a presence of boreal trees in the narrow canyons adjacent to the valley. Larch (*Larix sibirica*), Siberian pine (*Pinus sibirica*, *Pinus silvestris*), and White Birch (*Betula platyphylla*) occur on north facing slopes where the soils retain enough moisture to support them (1). About 85% of the annual precipitation (250-300 mm) falls during the warm season, between June and September. The annual average temperatures are between -0.9°C and -1.6°C (with minima of -46.2°C) and the landscape is characterized by the Selenge-Orkhon forest-steppe.

Since the 1970s, systematic surveys led to the discovery of ca. 40 Palaeolithic localities in the Tolbor valley (Fig. S1) while recent surveys revealed that adjacent valleys were occupied during the same periods. Most of the artefact concentrations were identified along the Western flanks of the valley and referred to as ‘sites’. The first full-scale excavation took place between 2004 and 2007 at the stratified site of Tolbor-4. The project was led by members of the Institute of Archaeology and Ethnography, Siberian Branch of the Russian Academy of Science, and the Mongolian Institute of Archaeology (2). Between 2007 and 2011, the stratified site of Tolbor-15 was excavated under the field direction of one of the PIs, S.A. Gladyshev (3, 4).

1.2. Excavated areas

In 2010, surveys by S.A. Gladyshev and A.V. Tabarev led to the recognition of additional Palaeolithic stations. Tolbor-16 and Tolbor-17 were tested and recognized as potential stratified sites (5). The same year, two 2x1 m test pits separated by 35 m have been dug along the slope.

Pit 1 is located between the stream and one of the numerous stone mounds that covers the western hillsides of the valley (Figure S2-B). The main artefact concentration was found approximately between 1.70 m and 2.00 m from the ground surface, and no artefacts were reported below the cultural layer. In the initial test pits, artefact provenience was not systematically recorded, and sediments were not screened. A bone sample found in the lower part of the 2010 test pit yielded a first radiocarbon date. Although its association with the archaeology was not clear, it indicated a preservation of the collagen reaching the limit of the radiocarbon range (45.4 ka <, AA-93134) (6).

Between 2011 and 2016, the site was excavated by an international team under the field direction of N. Zwyns and B. Tsedendorj (7). During this period, 12 m^2 were excavated bringing the total surface to 14 m^2. The whole excavated area was brought down circa 2.2 m below the ground surface, until signs of human occupation could no longer be detected. One of the excavation units was dug for an extra 50 cm to test for signs of human occupation below the main concentrations.

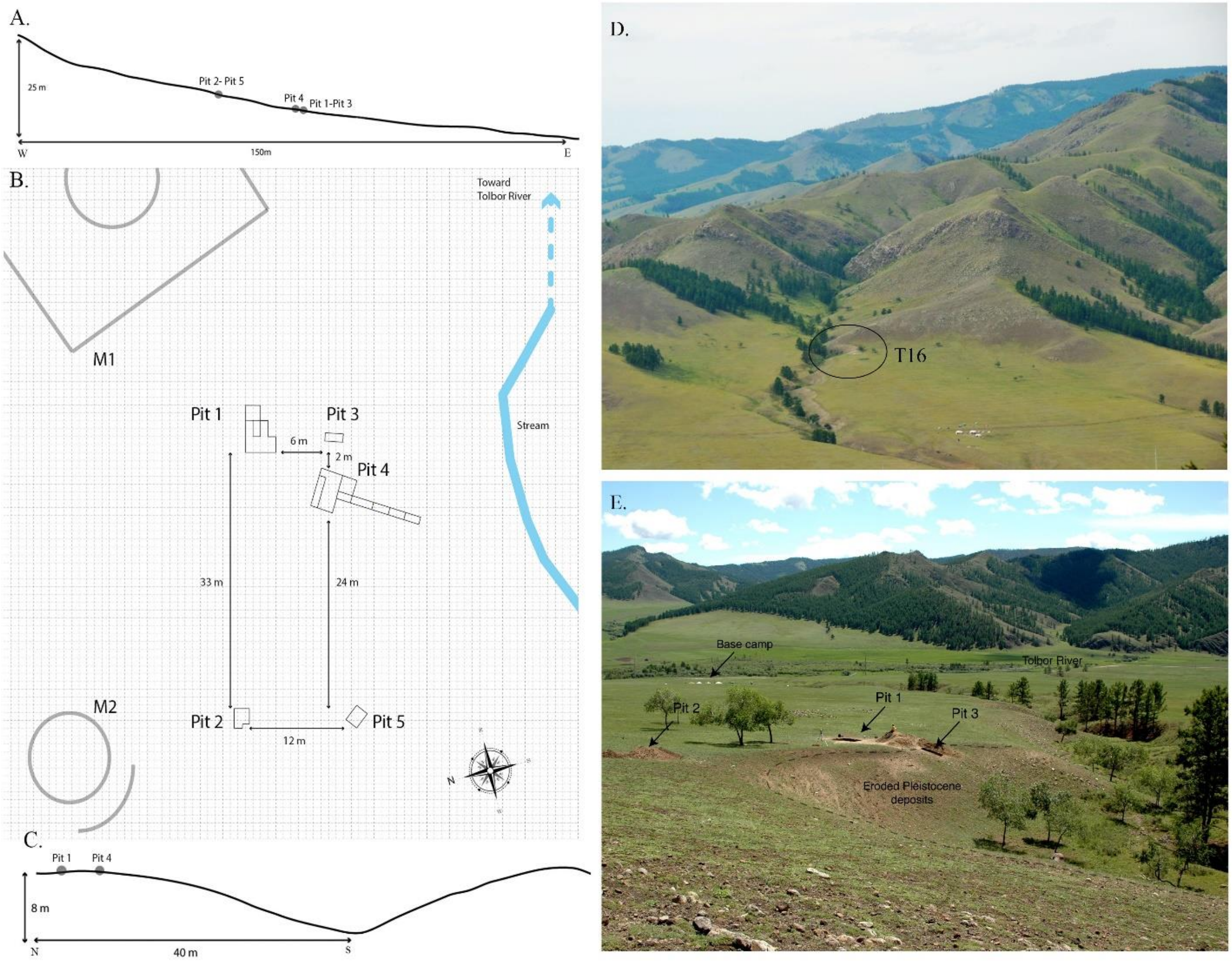

Figure S2: West – East longitudinal section of the Valley Flank with location of the excavation pits. B. Map with location of the excavation pits (M is stone mounds). C. North-South cross-sectional view of the stream. D. Western flank of the Tolbor Valley with the location of T16 site. E. T16 (2012, Pit 4 was not excavated yet), view from the West.

Pit 2 was first tested in 2012 and further excavated in 2012 and 2016. OSL samples (SI-section 3) were collected from the exposed section (Fig. S2E). Two additional units were opened to the south and brought down to approximately 2 m below surface. The sequence documents a time span from the MIS3 to the Holocene. The material is currently under study.

Pit 3 is small cut of circa 2 m^2 each opened for stratigraphic purposes and to evaluate the preservation of the archaeological layers. It has yielded MIS3 and MIS2 deposits rich in archaeological material.

Pit 4 was first opened in with a trench of 4 x 1m was dug upslope along the meander. The goal was to take advantage of the erosion to reach deeper deposits. In 2014, the trench was extended by 1m to the East and 3m to the west, making 15 m^2. In 2015, we dug steps along the erosional surface of the meander down to the stream for stratigraphic purposes. No archaeological material was found below 1.40 m.

Pit 5 is a cut opened in 2014 in order to confirm the depth of the Unit 2/ Unit 3 interface in the upper part of the slope.

2.1. Excavation method

The data collection is adapted after McPherron & Dibble (8). We used a total station for mapping the general topography of the site and the anthropogenic features but also to locate in place artefacts bigger than 2 cm in length. When possible, we measured the orientation and inclination of the long axis. Within stratigraphic units, we excavated sediments by arbitrary spits of 5 cm thickness and dry-sieved using a 4- and 2-mm mesh. We recorded sample locations with the total station. We used a Trimble Nomad (with EDM CE software) and then transferred to MSAccess before being processed in New-Plot GIS software (8, 9). Graphic documentation consists of geo-located digital pictures, hand-made and digital mapping, 3D models and situational sketches.

2. Sediment analyses

At Pit 1, bulk samples for analysis were collected at 5cm intervals in a column extending from the top to the base of the section. Within unit 3, careful sampling ensured that samples came from single laminae within the finely laminar sediment, and the sampling interval varied as a result. Blocks for micromorphological analysis were also collected from the section (see figure S5). At Pit 4, bulk samples for analysis were collected every 5cm along a column extending from the top to the base of the north wall of the excavation, including a test pit and a stepped trench cut down the side of the gully (see figure S6). The total depth of the sampled sediment is 255cm.

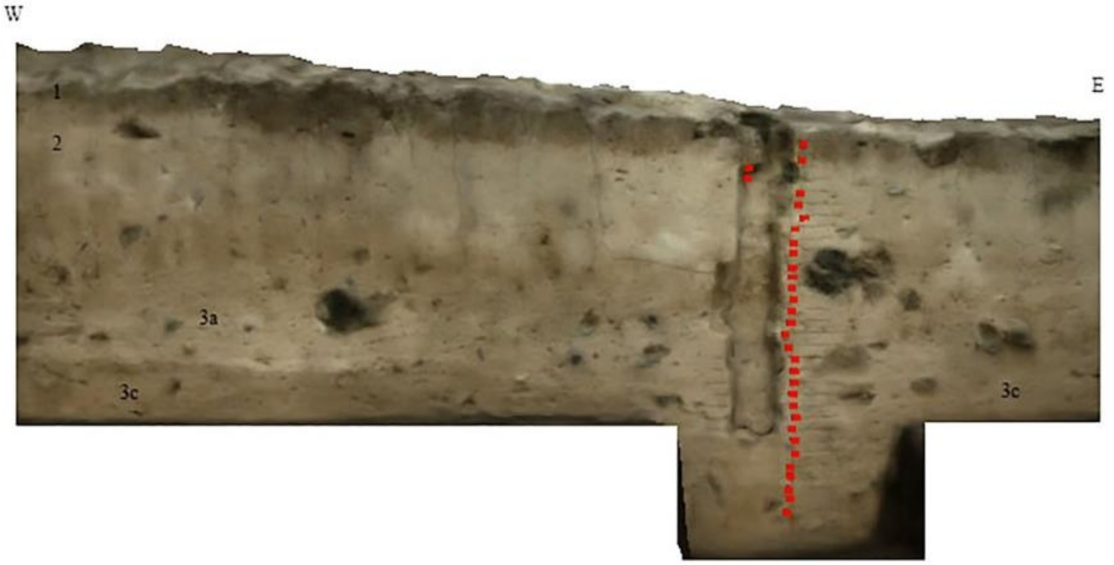

Figure S3: Sampling locations for bulk sediment analyses in Pit 4, north wall (Samples 1-32). Geo-localized 3D photogrammetry in Agisoft Photoscan, data collected in New Plot (8, 9) 3D plot generated in ISTI-CNR MeshLab.

2.2. Laboratory Methods

Screening and weighing, loss-on-ignition, magnetic susceptibility, and laser particle size analyses took place in the department of Geography, University of Cambridge. For loss-on-ignition analysis, samples were placed in a weighed crucible and successively heated to 400, 480, and 950°C in a muffle furnace. Changes in sample mass after each heating indicate the proportion of uncarbonized and carbonized organic matter and calcium carbonate, respectively, in each sample. Magnetic susceptibility was measured on a Bartington Instruments magnetic susceptibility meter. Screened samples were dehydrated, weighed into pots, three measurements were taken and the results averaged. Laser particle size analysis was carried out using a Malvern Mastersizer 2000. Screened samples (<2mm) were disaggregated with sodium hexametaphosphate for 24 hours. A vortex mixer was used to agitate the samples before their introduction into the Mastersizer.

Blocks for micromorphological analysis were prepared in the McBurney Laboratory for Geoarchaeology, Department of Archaeology, University of Cambridge. The intact blocks of sediment were air dried, then oven dried for 48 hours to drive off any remaining moisture, whose presence might disrupt the subsequent impregnation of the blocks with clear resin. The dry blocks were immersed in a preparation of polyurethane resin thinned with acetone in the ratio 9:1, and with MEKP in a minute quantity (0.00002%), and were

placed in a vacuum chamber to ensure that the resin penetrated to the center of the blocks and filled all of the voids and pore spaces within them. More resin was added as needed to ensure complete immersion of the blocks. The samples were then allowed to cure for two months at room temperature. Slices of the now-hardened blocks were cut using a rock saw, temporarily mounted on glass, lapped on a Brot thin section machine to create a smooth surface, permanently mounted on prepared glass slides using polyurethane resin, lapped to a thickness of around 30 µm and hand-finished by sanding, then cover slipped. Three of the thin sections were prepared by Julie Miller (Earthslides). The thin sections were analysed using petrographic microscopes in plane and cross-polarized light.

2.3. Description of the stratigraphy

<u>Tolbor-16 Pit 1:</u> The sediments at Pit 1 can be divided into three main units (Figure S5a): the Holocene soil complex (unit 1; depth 0-69cm; samples 1-14), a thin deposit of light brown loess with sparse gravel (unit 2; depth 70-114cm; samples 15-24) and soliflucted humic patches at the contact with the underlying unit (depth 115-129cm; samples 24-26), and a thick deposit of soliflucted laminar silt with gravel and cobbles which extends to the bottom of the section (unit 3; depth 130-185cm; samples 27-43). The results of lab analyses are presented alongside the drawing in Figure S5b-h, which is the west wall of the excavation, transverse to the direction of dip. Unit 1 is subdivided into sub-units 1a (the recent soil; a kastanozem (FAO); depth 0-15cm; samples 1-3), 1b (the A-horizon of a buried chernozem; depth 16-55cm samples 4-11), and 1c (probably the B-horizon of the chernozem; depth 60-69cm; samples 12-14). The interface of units 2 and 3 is marked by slope change and a solifluction event which disrupts the stratigraphy at this point (115-129cm). Material from the two units is mixed at this interface, and soliflucted humic patches are also observed here. Unit 3 is subdivided into sub-units 3a (depth 130-139 cm; samples 27-32), 3b (depth 140-159cm samples 33-38), and 3c (depth 160-185cm; samples 39-43), a succession of solifuction lobes. The thickness of unit 3 above AH5 (subunit 3c) is much greater here than in Pit 4, and it appears that, due to solifluction, parts of these sediments are either thinner or missing within the sampled section at Pit 4.

<u>Tolbor-16 Pit 4:</u> The sediments at Pit 4 can be divided into 4 main units (Figures S4 and S6a): the recent soil, a light brown kastanozem (unit 1; samples 1-3, 0-15cm depth), a thin (truncated) deposit of light brown loess with sparse gravel and humic patches toward its base (unit 2; samples 4-9, 16-49cm depth), a thick deposit of soliflucted laminar silt which extends to a depth of about 2.5 meters (unit 3; samples 10-32, 50-230cm depth), and a thin grain-supported deposit of sorted gravels (unit 4; samples 47-52, 231-255cm depth), possibly marking the top of a deeper sequence of fan-type sediments. Unit 3 is subdivided into solifluction lobes 3a (a stone-banked lobe to the west of the section, not sampled), 3b (samples 10-19; 50-99 cm depth), 3c (samples 20-23, 100-119cm depth), 3d (samples 24-26, depth 120-134cm) and 3e (samples 27- 46, depth 135-230cm). As at Pit 1, the interface between units 2 and 3 is marked by an episode of slope change – resulting from the initiation of loess deposition – and solifluction which truncates unit 3 and mixes material from units 3 and 2 along the contact. Section drawings for the main excavation are presented in Figure S4a and b; results of analyses are presented alongside a deeper section drawing (compiled from the steps cut into the hill) in Figure S6.

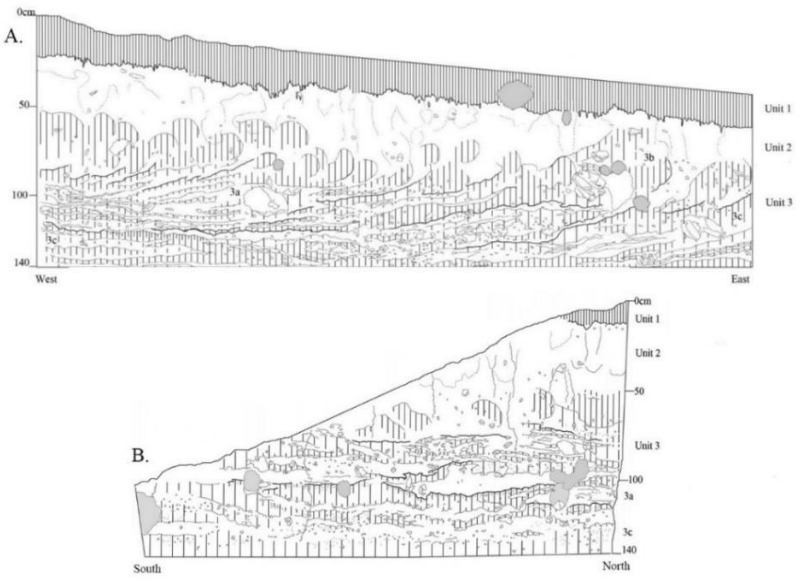

Figure S4: Pit 4 stratigraphic section. A. North Wall, B. West wall.

2.4. Sedimentological analyses

Loss-on-Ignition: The results of loss-on-ignition analysis at Pit 1 are presented in figure S5b (carbonate content, grey line), c (carbonized organic matter, blue line) and d (uncarbonized organic matter, orange line). The results of loss-on-ignition at Pit 4 are presented in figure S6b (carbonate content, grey line), c (carbonized organic matter, blue line), and d (uncarbonized organic matter, orange line). Particularly high organic matter and calcium carbonate contents are recorded for sub-units 3c, 3d, and 3e.

Magnetic susceptibility: The results of magnetic susceptibility analyses at Pit 1 are presented in figure S5h, and at Pit 4 are presented in figure S6h. Magnetic susceptibility varies through the sections but not systematically. The highest measured value at Pit 4 is within unit 2. A small peak occurs in the gravel horizon of unit 4, perhaps because of the lithology of the gravel.

Particle size analysis: Gravel contents (measured by screening and weighing the sediment samples) at Pit 1 are presented in figure S5g and at Pit 4 are presented in figure S6g. Figure S5d and e, and S6d and e are derived from laser particle size analyses of the fine fraction (>2mm). Figures S5d and S6d present the standard deviation within the analysed samples; primary loess is typically very well sorted with a low standard deviation, while weathering, soil development, and colluviation all raise the standard deviation within the samples for different reasons. Where standard deviation within the sections is low, the dominance of eolian processes is presumed. Figure S5e and S6e present the mean particle size of the silt fraction. This is intended as a measure of the energy of deposition; sand and clay are excluded because they may reflect post-depositional processes. Figure S7 presents the proportions of clay, silt, and sand (S7b) and particle size distribution curves for all of the sampled sediments at Pit 4 (S7c). All of the analysed sediments display a mixture of sand, silt, and clay (despite gravel contents as high as 22% by weight). The plots in figures S5d, e, and f, S6d, e, and f, and figure S7 confirm that multiple, generally low-energy sedimentary processes affect the sampled sediments.

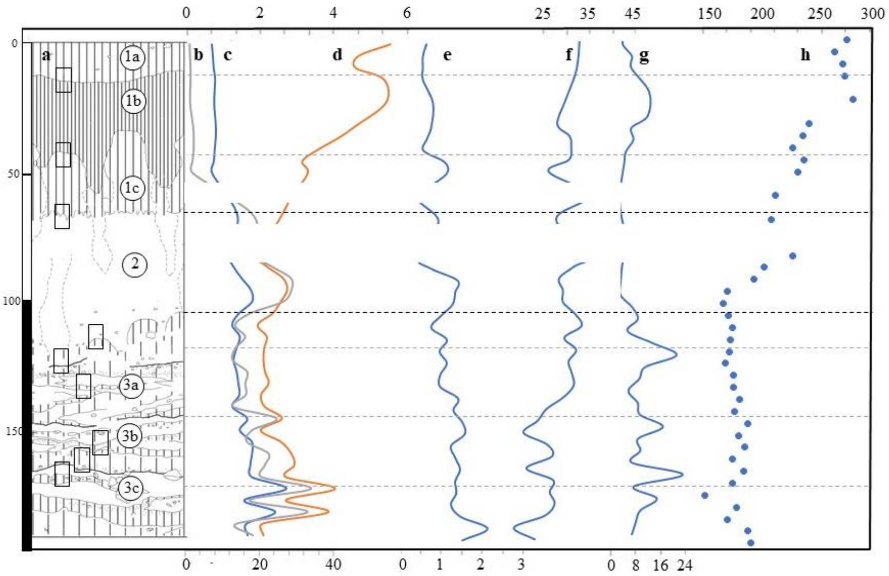

Figure S5. The Western wall at Pit 1 (a) and the results of sedimentological analyses. Rectangles: micromorphology. Shading indicates sediment color. b. Calcium carbonate content (grey line, %, bottom axis). c. Carbonized organic matter (blue line, %, top axis) and d. Uncarbonized organic matter (orange line, %, top axis) by loss-on-ignition. Gaps are samples which were removed by customs officials. e. Standard deviation within the fine (<2mm) fraction, by laser particle size analysis. The extent to which the sampled sediments are dominated by loess-like sedimentation (low standard deviation) or sheet erosion or soil development (higher standard deviation) may be a proxy for precipitation. f. Mean particle size of the silt fraction (µm, top axis). Coarser particles mean higher-energy deposition. g. Gravel content of the sampled sediments (weight %, bottom axis). Gravel represents gravitational input. Increased gravel content indicates longer surface exposure and thus slower sedimentation, and the distance between e and f indicates surface stability. h. Magnetic susceptibility (SI). Variation below the Holocene soil complex is difficult to explain, and may reflect the lithology of the local bedrock and its varying presence within the section.

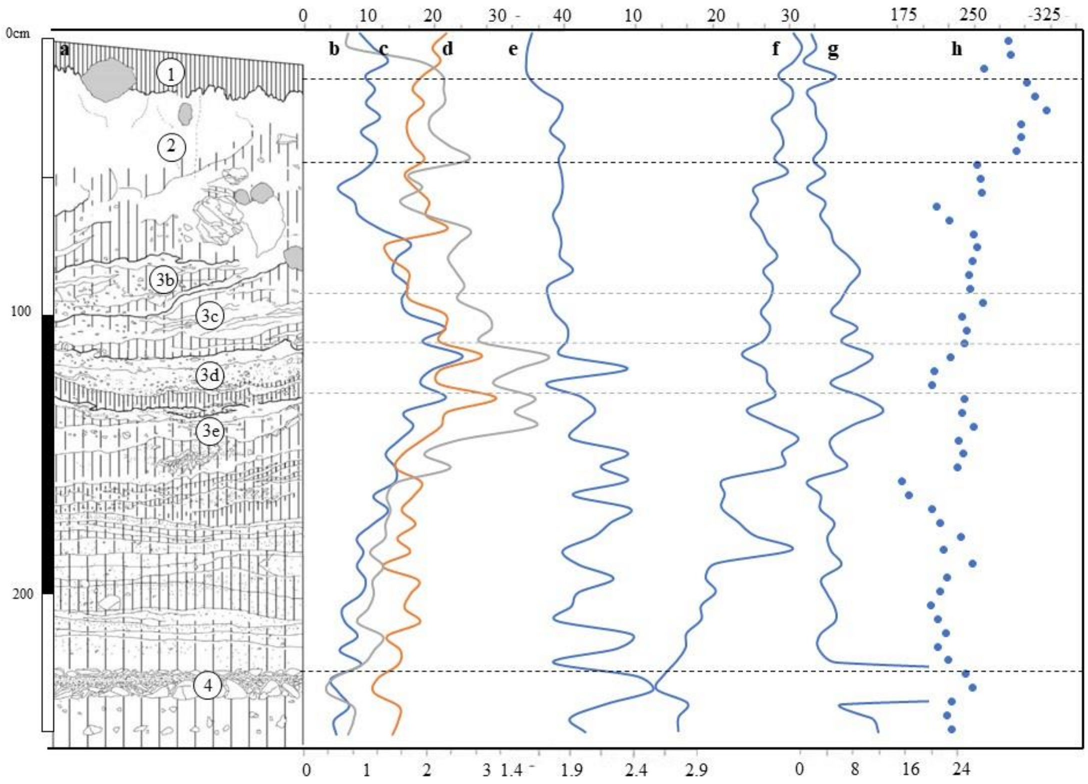

Figure S6: a. The sampled section at Pit 4, including a stepped trench below the main excavation, and the results of sedimentological analyses. Shading indicates sediment color. b. Calcium carbonate content (grey line; %, top axis), c. Carbonized organic matter (blue line, %, bottom axis). d. Uncarbonized organic matter (orange line, %, bottom axis) and in the sampled sediments, by loss-on-ignition. e. Standard deviation in the fine fraction (<2mm) by laser particle size analysis. The extent to which the sampled sediments are dominated by loess-like sedimentation (low standard deviation) or sheet erosion or soil development (higher standard deviation) may be a proxy for precipitation. f. Mean particle size of the silt fraction (μm, top axis). Coarser particles mean higher-energy deposition. g. Gravel content of the sampled sediments (weight %, bottom axis). Gravel represents gravitational input. Increased gravel content indicates longer surface exposure and thus slower sedimentation, and the distance between e and f indicates surface stability. h. Magnetic susceptibility (SI). Variation below the Holocene soil complex is difficult to explain, and may reflect the lithology of the local bedrock and its varying presence within the section.

2.5. Interpretation

<u>The geomorphological situation</u>

The T16 site sits on a solifluction terrace, a relatively flat-topped landform with a steep scarp formed by the superposition of successive lobes of soliflucted material, and is considerably elevated above the floodplain of the Tolbor River. Although of Holocene origin, the present-day gully which dissects the landform follows the course of a drainage – incised into the bedrock – of considerable antiquity; before the Holocene a cone of sediments existed at its mouth. The material of the solifluction terrace seems to have been thickest by the opening of the drainage into the wider Tolbor valley and dips away from this point, and it is likely that the redeposited material of the sheet erosion deposits of unit 3 also moved along the drainage. Somewhere beneath these sediments lies a bajada – visible elsewhere along the valley and in roadcuts – of which the gravels of unit 4 may mark the top. The rock-cut drainage, and others like it along

the valley, probably supplied the coarse material for this earlier fan deposition as well. The geomorphological situation at T16 is important to the sedimentary and stratigraphic interpretation presented here because of the role of water in the deposition of unit 3. Water – as an agent of transport and in the form of soil moisture – is the cause of the sediments' laminarity, which is described in more detail below. The position of the sections adjacent to and even – in the lower excavated steps of Pit 4 – within the drainage should probably be borne in mind when considering the hydrological regime at the time of site formation.

The loess and loess-like sediments of units 1 and 2 at Pit 4

Particle size analyses confirm eolian sedimentation as a dominant process during the time periods represented by the deposition of units 1 and 2. Even in the Holocene soil of unit 1 the organic matter content is relatively low, and the particle size distribution curve is consistent with loess (possibly with some removal of the very fine fraction by washing). The explanation for this is that Holocene gully incision truncates the loess deposit of unit 2 (substantially thicker in Pit 1 just a few meters away), and a relatively thin, weak soil forms at the surface. Unit 2 contains sparse gravel (increasing with depth). Particle size distribution curves (figure S7c), while still indicating the dominance of eolian sedimentation, suggest that the role of colluviation increases with depth in the sediments of unit 2. The contact with unit 3 is erosional and, in the sampled section, thick owing to solifluction. A slope change is evident from the dip of the sediments; loess accumulation against the hillside likely resulted in a steeper profile. The development of a weak soil (samples 10-13) and its subsequent reworking by solifluction (following this new, steeper slope) result in local truncation of the underlying sediments and the admixture of material from unit 3.

The textural laminae of unit 3 at Pit 4

The laminar silt of unit 3 is very poorly sorted, and clay and very fine silt are included in the same deposit as cobbles and even boulders. In thin section (figure S8), the mineralogy of the coarse (>approx. 100µm) and fine fractions within the laminar sediment are different, with the former comprising fragments of the local igneous bedrock, and the latter dominated by silt-sized grains of quartz, which represent eolian sedimentation (loess); a loess parent material for the sediments is likewise indicated by the predominance of silt within the section (figure S7b and c). The mineral fraction of the laminar sediment has therefore at least two sources (one proximal and one distal). Within unit 3 the quartz silt is mostly incorporated into inhomogeneous aggregates of more and less humic material (Figure S8a and b). Sand stringers (again of the local bedrock; Figure S8a and b) and fragments of the calcium carbonate pendants which form under larger clasts are all suggestive of reworking of the sediments, particularly by flowing water. With a few exceptions, most of the samples from unit 3 show particle size distribution curves (figure S7c) consistent with reworking (more gently peaked curves, multiple peaks) or soil development (enhanced clay content). Although loess is the parent material, the dominant depositional process within unit 3 seems to be sheet erosion.

Gravel, cobbles, and boulders are too big to have been transported in what appears to have been a low energy reworking. Some of the sand and gravel seem to have actually been generated post-depositionally by frost action and mechanical weathering during solifluction (larger cobbles are physically crushed to lenses of gravel), but the arrival of the larger clasts within a sandy silt matrix is presumably due to gravitational input from the bedrock outcrops just upslope. The coarser clasts are not uniformly distributed through the section; instead particular layers seem to play host to lines of cobbles or gravel. Although the bedrock source may have experienced climate-dependent changes in the rate of clast production (likely

through frost shatter), in general the presence of larger clasts seems to indicate a decrease in the rate of sedimentation (surface stabilization, reduced colluviation and sheet erosion) resulting in longer surface exposure. Lines of cobbles sit at the surfaces of some the solifluction lobes identified in the section drawing; the gravel content of the sampled sediments (figures S5g and S6g) is here used as a proxy for sedimentation rate, with lower gravel content – because of the multiple sedimentary processes involved in the deposition of the studied sediments – indicating more rapid burial, and higher gravel contents indicating prolonged surface exposure and slower sedimentation (an exception is unit 4 at pit 4, the gravel deposit). The mean silt particle size (figures S5f and S6f) varies inversely with gravel content, indicating two separate depositional processes for the two size fractions.

The compositional laminae of unit 3 at Pit 4

The color variation within the laminar deposit, from dark brown to white, is due to variations in calcium carbonate content and, to a lesser extent, organic matter (figure S8c and d). While the source of the calcium carbonate within the sediments is probably loess, certain laminae within unit 3 have carbonate contents well above that of the primary loess of unit 2. There doesn’t seem to be a very strong relationship between the particle size distribution of the fine fraction and calcium carbonate content, and these two variables probably reflect different processes; increased carbonate content is not simply a product of loess accumulation. In thin section, calcium carbonate enrichment within lighter laminae includes the precipitation of calcium carbonate pendants on lithics and coarser clasts (these are almost always on the bottom), as well as micritic calcite precipitation on the groundmass of aggregates, and is therefore likely post-depositional. There is some evidence that the enrichment forms at or very near to the ground surface (pronounced white laminae frequently co-occur with lines of cobbles, and often display higher organic matter contents than the over- and underlying sediments). The calcium carbonate enrichment is laminar in character but does not seem to form nodules or coat smaller grains in thin section as is usually expected for a k-horizon. Apart from disaggregated grains of root cell calcite, which can be abundant, calcium carbonate does not seem to be related to roots, pores, or voids (although, since these are subsequently affected by frost action, which merges them to a lenticular microstructure, it can be hard to tell); it exists as micrite precipitated directly on the groundmass from the soil solution. Its finely laminar character, alternating with less carbonate-rich laminae, is most consistent with crust formation. In the sedimentary regime represented by most of unit 3, crusts are buried by colluvium, creating compositional laminae.

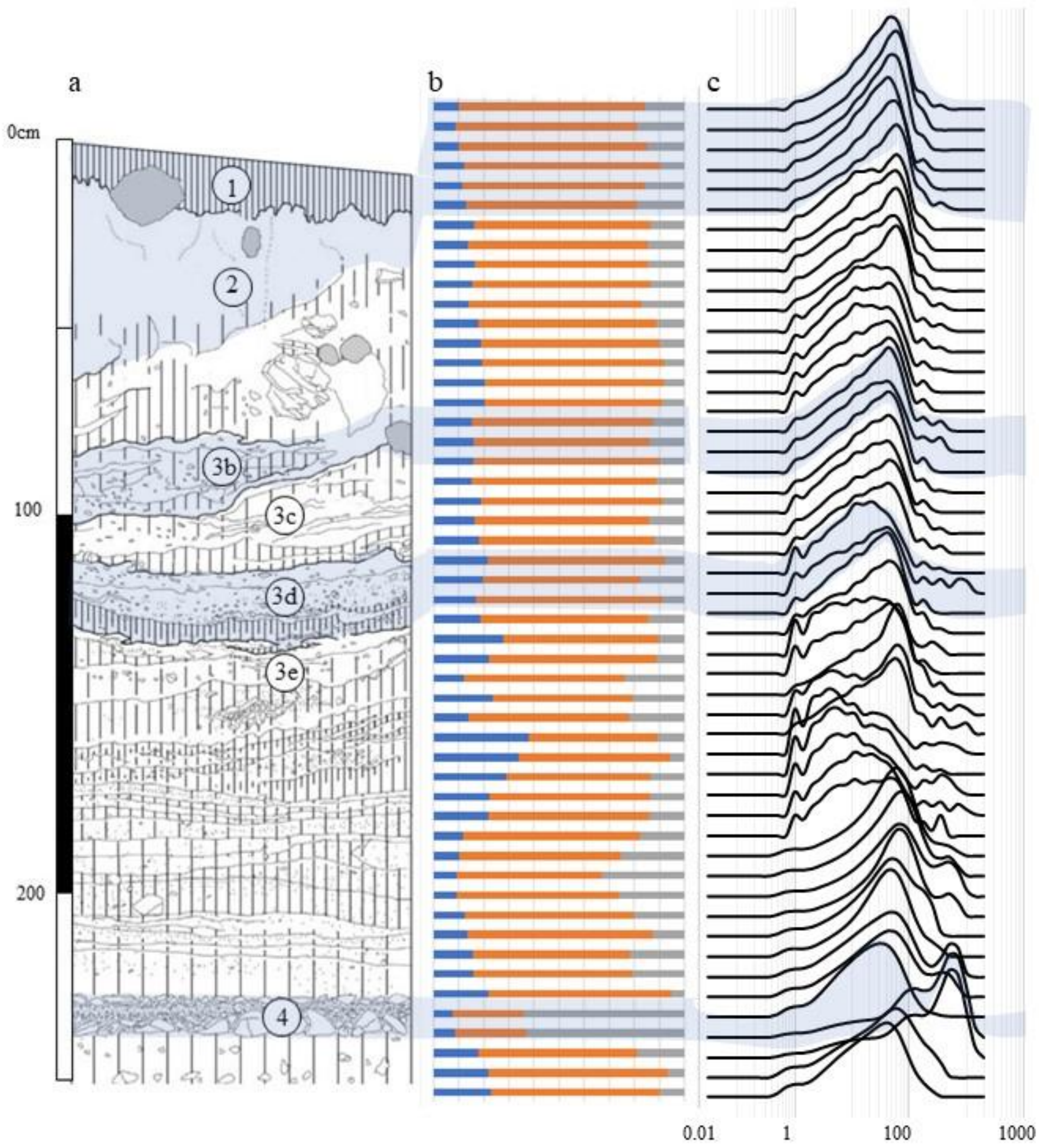

Figure S7. a. The sampled section. b. Proportion of clay (blue), silt (orange), and sand (grey) in the sampled sediments. c. Particle size distribution curves for each of the sampled sediments. The best-sorted sediments (sharply peaked) represent primary loess or reworked loess. Although loess is the parent material for most of the sampled sediments, secondary sorting events (reworking, colluviation, redeposition by flowing water) or weathering and soil development (the breakdown of coarser silt particles, clay enrichment) change the shape of the curves. Blue shading is intended to distinguish stratigraphic units.

The question of what climatic situation is represented by the combination of calcium carbonate enrichment, stone or gravel lines, and increased organic matter is important, since it relates to the sediments which house AH6. The enhanced organic matter content alone is indicative of soil formation, and it looks like the reworked soil material in unit 3 shows various degrees of soil development through the section according to the climate at the time of pedogenesis. The repeated episodes of sheet erosion which form unit 3 are

linked with precipitation, and the alternation of sheet erosion and the development of thin surface crusts of evaporites (the fact that strong evaporation frequently follows precipitation) tells us something about the seasonality of that precipitation. Either the very thick white laminae at the surfaces of solifluction lobes 3c,d, and e form during soil development, growing more pronounced as colluviation slows with longer-term surface stability (and therefore the soil itself, which in some cases is even more strongly humic than the present one, reflects hydrological conditions very different than the present-day), or they form in a cold arid period just after the end of soil development. One possible scenario is a moist active layer sitting over permafrost or seasonally frozen ground, with arid conditions in particular seasons bringing about strong evaporation.

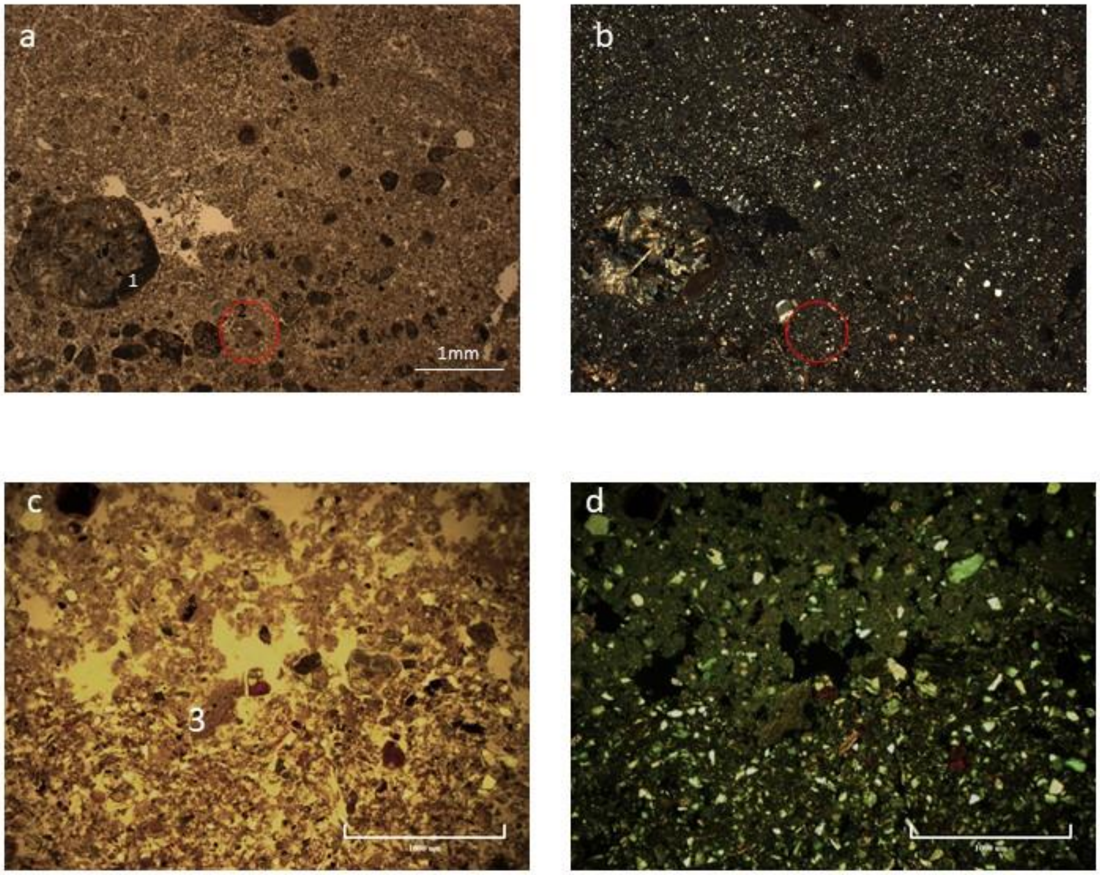

Figure S8. Textural and compositional laminae in unit 3 at Pit 1. a and b: Sand stringers under PPL(a) and XPL(b). A redeposited aggregate of soily material is circled (b). A carbonate pendant on grain of coarse sand is marked 1 (a). c and d: compositional laminae in unit 3 at Pit 1 under PPL (c) and XPL(d). A carbonate-rich lamina (top half) glows brightly under XPL, compared to the less carbonate-rich material in the bottom half of the images. A redeposited fragment of a crust or coating is marked 3 (c). The different mineralogies of the silt (quartz; white and grey specks), and sand fractions of the sediments are evident in the XPL images (b and d).

The climatic record

The variations in organic matter and carbonate content, sedimentation rate (for which gravel content is used here as a proxy), and in the predominance of high and low energy processes and eolian or water-mediated sedimentation in the deposition of the section can be put together into a climatic record for the sediments of Pit 4. This record is augmented – but also disrupted – by the episodes of solifluction which took place intermittently until the initiation of loess deposition in unit 2. We should probably assume a lacuna where solifluction lobes meet, and there may be some repetition or thickening of the sediments; owing to the small area of exposure below 3c, it is difficult to say whether 3d is a repetition of 3e, and within the column of samples it appears that top of lobe 3c is caught up in the base of solifluction lobe 3b, locally doubling the sediment. Lobe 3a is not sampled (see figures S3 and S4), but radiocarbon dates make it the same age as 3c. Finally, the solifluction event at the base of unit 2 truncates unit 3 (probably to a different depth than at Pit 1), which means that some of the climatic record is missing. These are the interpretive challenges the section presents, but bearing them in mind, it nevertheless preserves a fairly high-resolution climatic record.

Carbonate content is generally lower in the bottom half of the section (as yet undated). Together with particle size distribution curves consistent with sorting by flowing water (figure S7c), and relatively low organic matter contents, we can infer cool and wet conditions during the deposition of unit 4 and most of lobe 3e (bearing in mind that because the trench was stepped, these samples are closer to the mouth of the bedrock drainage which supplied sediment and moisture). Within unit 3, precipitation (as evidenced by sheet erosion; figure S7c) seems roughly to covary with organic matter content; areas of clay enrichment may reflect the deposition of reworked soils or in situ clay development and translocation. Both organic matter and carbonate content peak within these sediments; the combination of sheet erosion, soil development, high organic matter content, and high carbonate content with crust formation collectively suggest a different hydrological regime than today. Changes in the seasonality of precipitation may be invoked to account for the covariation of seemingly opposed processes and constituents. While organic matter content declines above lobe 3c, carbonate content remains generally high, suggesting overall cooler and more arid conditions; a change from sheet erosion to eolian deposition takes place above lobe 3b in the sampled section.

The climatic cyclicity implied by repeated episodes of solifluction is confirmed by other proxies – apparent warm events in sub-units 3c, d, and e see increased organic matter content, sheet erosion, evidence for soil development including increased clay content, and a reduction in the overall rate of sedimentation, followed by climatic deterioration and a change toward eolian sedimentation. Despite possible gaps and repetitions in the record, the sediments within unit 3 seem broadly to confirm a series of (at least two) warmer climatic episodes interspersed with relatively serious deteriorations. That a climatic deterioration takes place at the top of sub-unit 3c, which houses AH6, is supported by a change to loess-type sedimentation and a reduction in organic matter content in the uppermost part of this lobe. Regardless of any hiatus or doubling between the top of 3c and the overlying sub-unit 3b, we can associate this climate deterioration chronologically with end of the occupations of AH6. Aside from solifluction, evidence for this climate crash at Pit 1 includes the formation of a thick white (calcium carbonate-rich) lamina, most likely connected with arid conditions and a reduction in the number of heavy rainfall (sheet erosion) events.

3. Luminescence Dating

The chronologic framework for human occupation at Tolbor 16, and for contextual landscape change in the surrounding valley, was determined by both radiocarbon and luminescence techniques. 16 luminescence samples were collected from Tolbor.

Luminescence dating determines the antiquity of the most recent exposure of sediment grains to sunlight, and hence the depositional age (10). Luminescence dating is a highly suitable method for determining the timing of human activity at archaeological sites such as Tolbor 16, where archaeological traces are clearly directly associated with the sediments in which they are found. Quartz is generally the preferred material for luminescence dating due to its more stable luminescence signal and negligible internal dosimetry; fine-grained wind-blown quartz has successfully been used for dating in comparable archaeological sites in the Eurasian landscape (11, 12). However, only 12 of the 16 samples collected yielded sufficient quartz for dating with luminescence. For these samples, both quartz and polymineral (feldspar-dominated) aliquots were measured for independent age determination and comparison. For all polymineral samples we used the post-infrared-infrared (pIR-IR) protocol at elevated temperature (290°C), which overcomes the problems of time-dependent fading of the feldspar luminescence signal (13). These protocols have been shown to be accurate with independent age control in Eurasian loess contexts (11, 14).

3.1. Sample collection

16 luminescence samples were collected from the Tolbor 16 profiles in Pits 1, 2 and 3 (Table S1, Fig. S9). Sampling was undertaken by driving stainless steel tubes horizontally into the cleaned profile. Approximately 300 g of additional sediment was collected from the same position as the luminescence samples, for analyses of moisture content and laboratory dosimetry measurements using gamma spectrometry (Fig. S9A). The sampling procedure aimed at getting ages for the three main sedimentary units (Unit1-3) and check for their consistency.

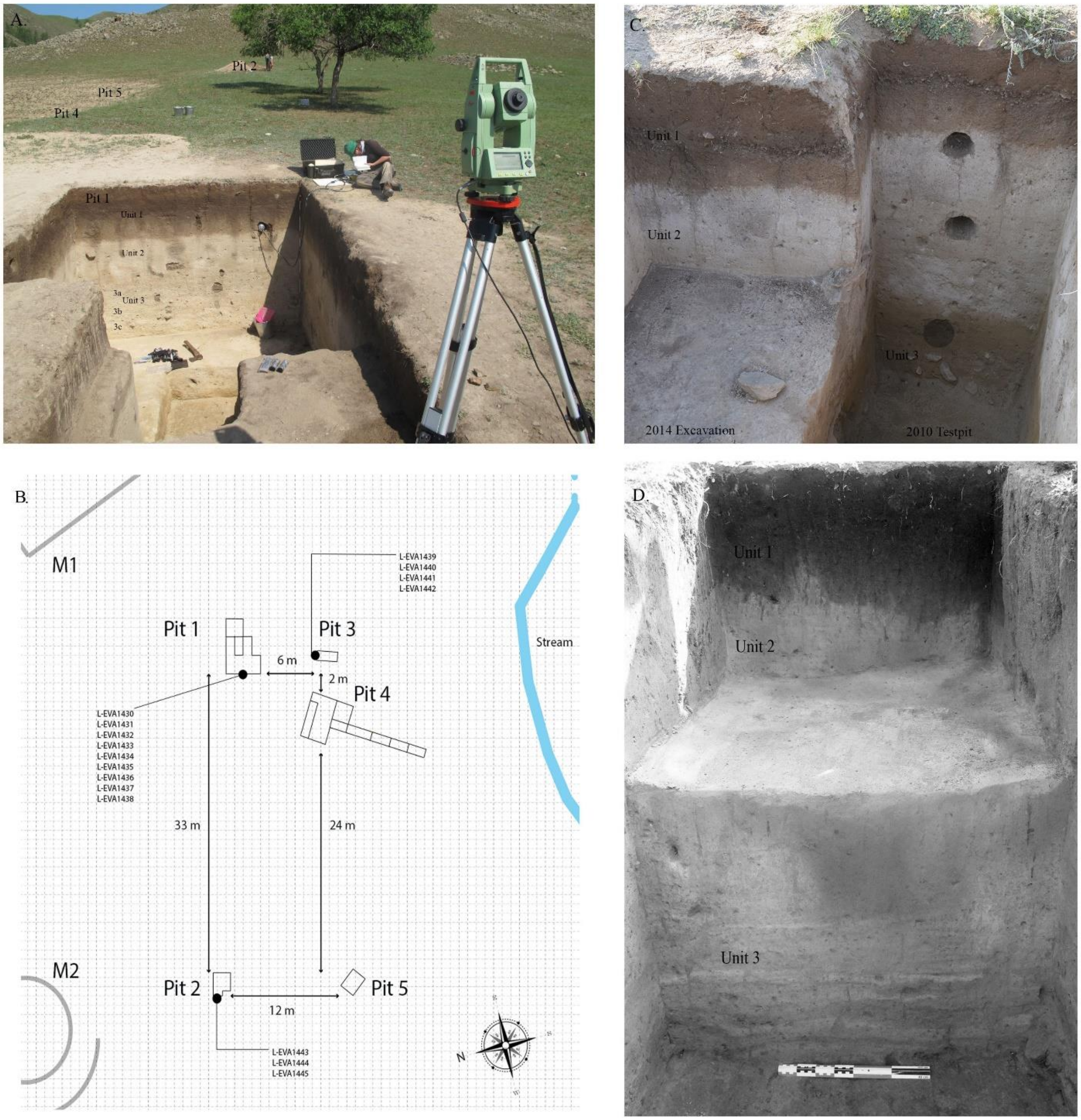

Figure S9: A. Sampling (L-EVA1430) and gamma spectrometry in Pit 1, Western Wall. B. Map with location of the OSL samples in Pit 1, Pit 2 and Pit 3. C. Pit 2, sampling in Western wall of Gladyshev's test pit (from top to bottom: L-EVA1443, L-EVA1444 and L-EVA1445). Only the top of Unit 3 is visible here and the lowermost sample provides a minimum age for it. D. Pit 3 is a ca. 2x1 m geological cut exposing Unit 1, Unit 2 and the top Unit 3.

Table S1. Description of samples with respect to test pit and stratigraphy. Depths attract an uncertainty of ± 0.05 m.

Field ID	Lab Code	Pit	Depth (m)	Unit	Comments
3044	L-EVA1430	1	0.6	1	Lowest organic unit
3048	L-EVA1431		0.73	2	Top
3052	L-EVA1432		0.97	2	Middle
3054	L-EVA1433		1.20	2	Bottom
3057	L-EVA1434		1.40	3a	Top, laminated unit
3062	L-EVA1435		1.60	3b	Middle, laminated unit
3065	L-EVA1436		1.90	3c	Bottom/whitish laminated unit
3046	L-EVA1437		2.06	3c/3d	Top, unit without artefacts
3050	L-EVA1438		2.17	3d	Lowest part, unit without artefacts
3071	L-EVA1439	3	0.47	2	Top
3073	L-EVA1440		0.75	2	Bottom
3075	L-EVA1441		0.82	3	Top, laminated unit
3077	L-EVA1442		1.15	3	Lowest part, laminated unit
3080	L-EVA1443	2	0.60	1	Top, no artefacts
3082	L-EVA1444		1.08	2	Middle, no artefacts
3084	L-EVA1445		1.70	2	Lowest part, no artefacts

3.2. Laboratory preparation and equivalent dose measurements

Sample preparation and measurement for OSL dating was undertaken in the luminescence dating laboratory of the Department of Human Evolution, Max Planck Institute for Evolutionary Anthropology, Leipzig. Sample preparation was completed under subdued red light using published methods (14-16). The fine-grained polymineral (feldspar-bearing) fraction (4-11 µm) was first isolated from other components of the sediment for measurement of the equivalent dose (D_e). This involved sediment settling to obtain the preferred grain-size fraction, removal of carbonates by digestion in HCl and of organic matter by digestion in H_2O_2. Fine-grained quartz was extracted from subsamples of the 4-11 µm polymineral fraction by etching in fluorosilicic acid, however not all samples yielded sufficient quartz for measurement following this process and since no further sample could be spared, only the polymineral fraction was measured for those samples. For each sample, 18 aliquots of 1cm diameter were prepared by pipette in water solution onto stainless steel discs for measurement.

Equivalent dose (D_e) measurements were undertaken using an automated Risø TL-DA-20 reader equipped with infrared light-emitting diodes for light stimulation of single aliquots (17), and with U340 and D410 filters inserted in front of the photomultiplier tube to detect the quartz and feldspar luminescence emissions respectively. Irradiation was provided by calibrated $^{90}Sr/^{90}Y$ beta sources (17).

Quartz aliquots were measured using the single aliquot regenerative dose (SAR) protocol of Murray and Wintle (18, 19), including an additional repeat dose at the end of the protocol which provided an infrared depletion ratio as a means to assess potential contamination of the quartz OSL signal by feldspar. Preheat

tests were conducted on three samples (Fig. S10) and indicated optimal preheat and cut heat temperatures of 220ºC and 200ºC respectively. Since the calculated dose distributions for each set of aliquots yielded Gaussian distributions (Fig. S11), the Central Age Model (20) was used to calculate the D_e for each sample.

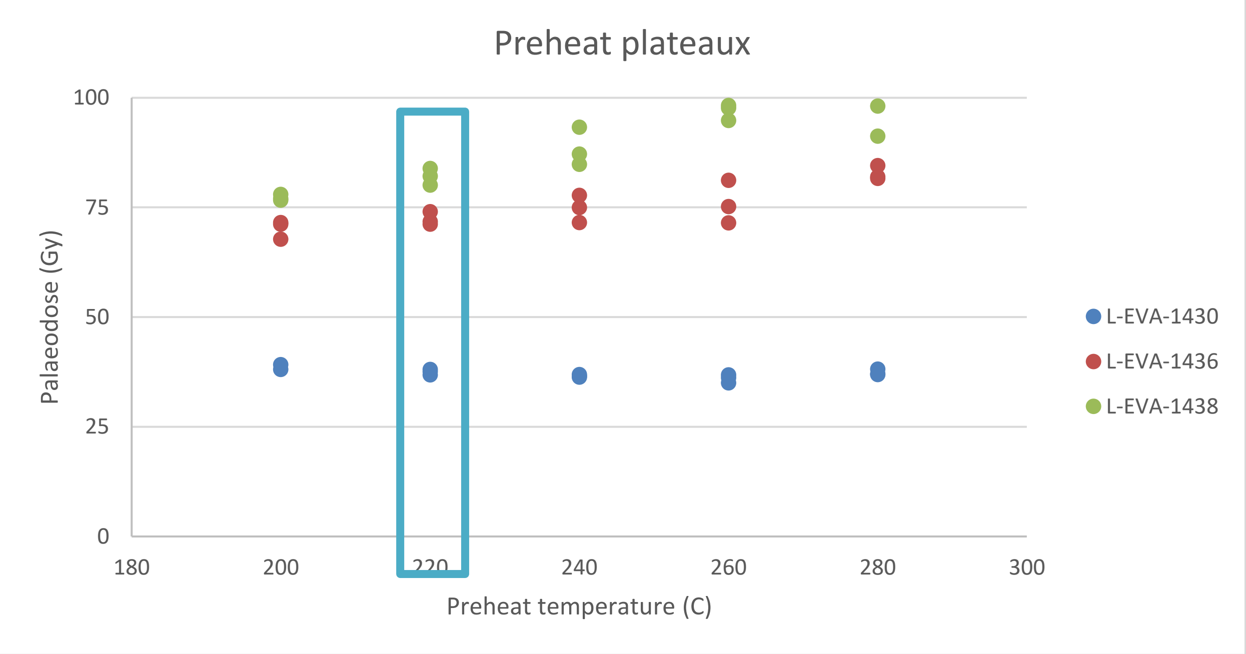

Figure S10. Preheat plateaux for quartz subsamples L-EVA-1430, L-EVA-1436 and L-EVA-1438 (top-bottom), showing optimal preheat temperature of 220C.

The pIR-IR_{290} protocol of Buylaert et al. (13) was applied to 18 polymineral aliquots of each sample, since higher preheat and measurement temperatures have been shown to reduce feldspar signal fading in loess (13; 21-23.

3.3. Luminescence characteristics

Both quartz and feldspar-bearing polymineral fine-grained aliquots yield very bright luminescence signals, which decay at rates typical for quartz and feldspar respectively (Fig. S11).

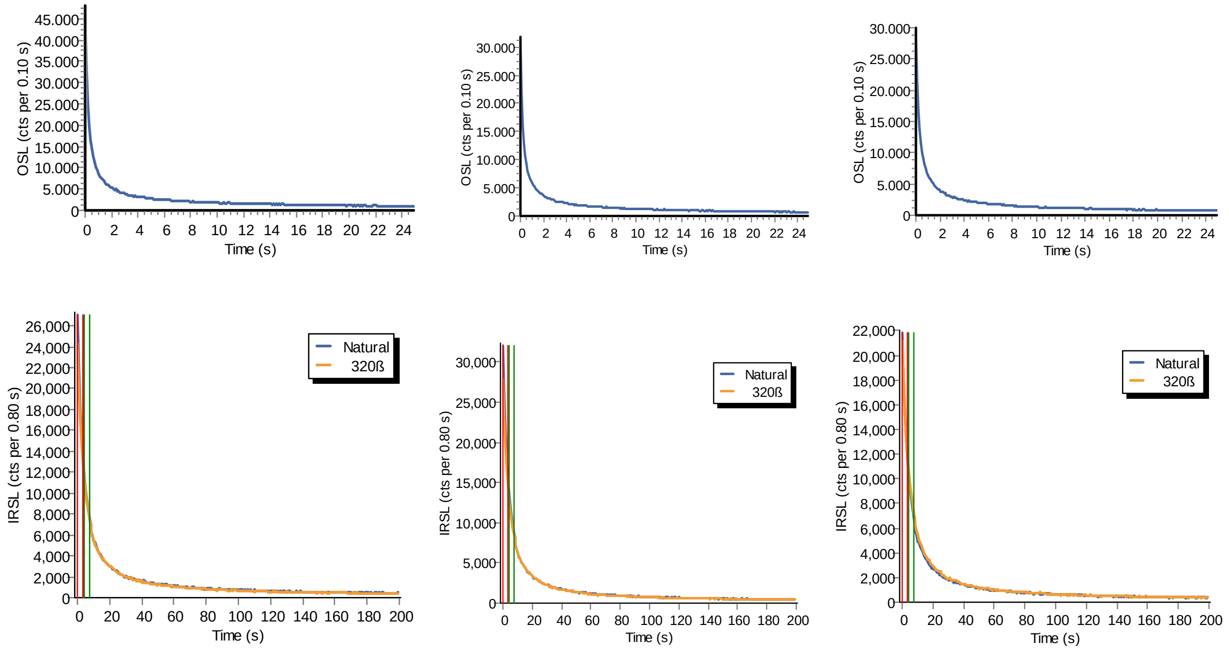

Figure S11. Luminescence decay curves for the natural luminescence signal for (top) three quartz aliquots of sample L-EVA-1436 (Unit 3-3), and (bottom) three polymineral aliquots of sample L-EVA-1433 (Unit 2-3).

The samples record variable, although acceptable, sensitivity change throughout the regenerative dose steps of the measurement protocol, remaining below a magnitude of two (Figure S12).

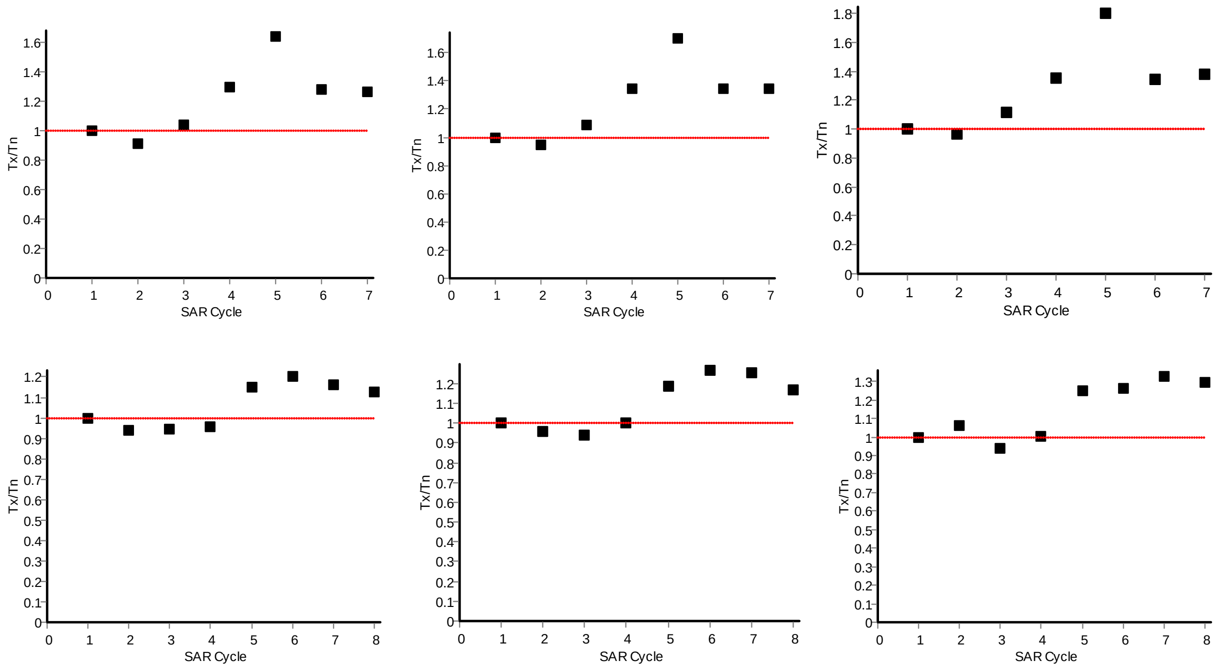

Figure S12. Quartz sensitivity change with respect to regenerative dose step for (top) three quartz aliquots of sample L-EVA-1436 (Unit 3-3), and (bottom) three aliquots of sample L-EVA-1433 (Unit 2-3), normalised to the first test dose. The zero dose step corresponds to SAR cycle 7, and the repeated regenerative dose (in this case c. 10 Gy) to SAR cycle 8.

The dose-response curves of all samples could be fit to a single saturating exponential or exponential-plus-linear function (Figure S13).

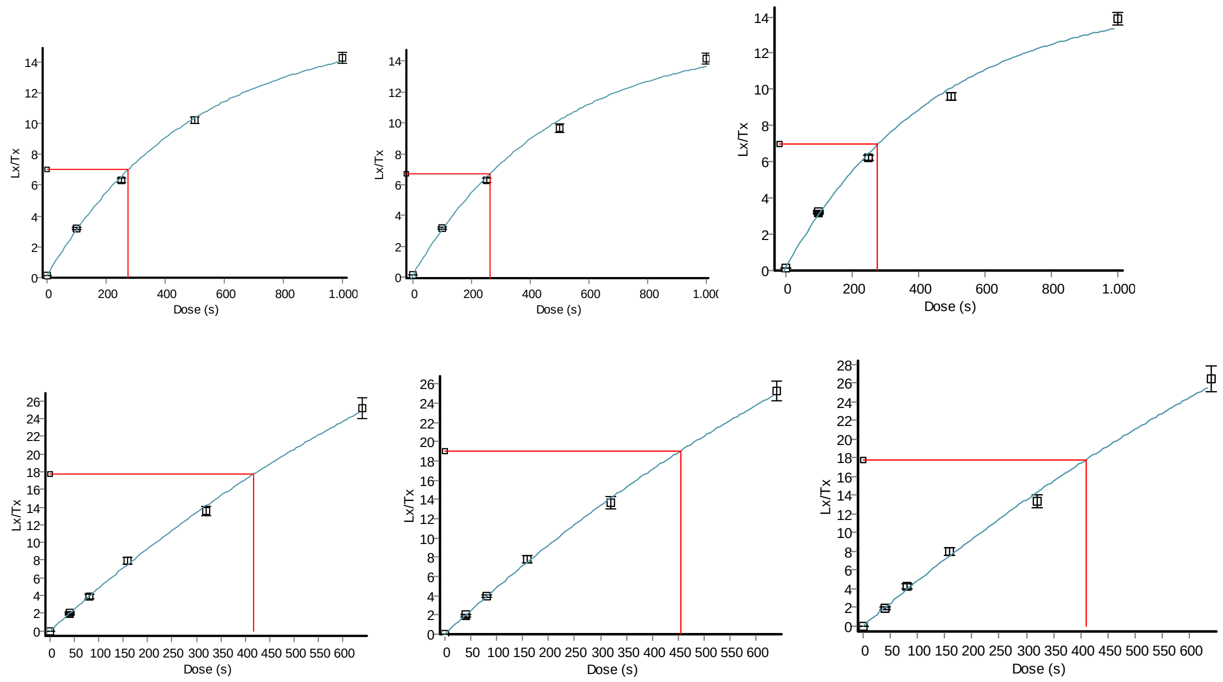

Figure S13. Quartz dose response curves for (top) three quartz aliquots of sample L-EVA-1436 (Unit 3-3), and (bottom) three aliquots of sample L-EVA-1433 (Unit 2-3).

Almost all aliquots, both quartz and polymineral, passed selection criteria for equivalent dose determination (Table S2). Mean recycling ratios lie within 5% of unity and overdispersion across dose distributions lies below 20% for all samples (and below 15% for all but one sample).

Table S2. Relevant statistics for luminescence dating of the Tolbor-16 samples.

		Quartz aliquots			Polymineral aliquots	
**Field ID**	**Lab code**	**Recycling ratio**	**IR depletion ratio**	**Overdispersion (%)**	**Recycling ratio**	**Overdispersion (%)**
3044	L-EVA1430	1.08 ± 0.02	1.08 ± 0.02	0.0	1.01 ± 0.13	10.9
3048	L-EVA1431	1.04 ± 0.04	1.04 ± 0.04	1.5	1.00 ± 0.07	5.6
3052	L-EVA1432	1.04 ± 0.04	1.03 ± 0.04	5.1	1.03 ± 0.12	18.5
3054	L-EVA1433	1.04 ± 0.02	1.05 ± 0.02	7.8	0.95 ± 0.09	5.9
3057	L-EVA1434	-	-	-	1.00 ± 0.12	12.2
3062	L-EVA1435	1.03 ± 0.02	1.03 ± 0.02	4.9	0.99 ± 0.11	4.5
3065	L-EVA1436	1.02 ± 0.02	1.01 ± 0.02	0.0	0.99 ± 0.05	2.6
3046	L-EVA1437	1.02 ± 0.02	1.03 ± 0.02	0.0	0.98 ± 0.08	8.8
3050	L-EVA1438	1.01 ± 0.01	1.01 ± 0.01	0.0	1.00 ± 0.07	14.2
3071	L-EVA1439	-	-	-	0.98 ±0.13	11.0
3073	L-EVA1440	-	-	-	0.94 ± 0.09	11.0
3075	L-EVA1441	1.05 ± 0.02	1.05 ± 0.03	0.0	1.03 ± 0.07	14.9
3077	L-EVA1442	-	-	-	1.02 ± 0.07	12.1
3080	L-EVA1443	1.07 ± 0.02	1.07 ± 0.02	0.0	1.00 ± 0.09	0.0
3082	L-EVA1444	1.06 ± 0.04	1.06 ± 0.04	4.3	1.04 ± 0.11	7.8
3084	L-EVA1445	1.03 ± 0.03	1.04 ± 0.03	0.0	1.00 ±0.13	4.0

Dose recovery ratios were calculated for representative quartz sample L-EVA-1430 (Unit 1) and two representative polymineral samples, L-EVA1433 (Unit 2-3) and L-EVA1438 (Unit 4-3). All fall within 10% of unity, indicating acceptable recovery of dose and suitability for dating (Figures S14 and S15).

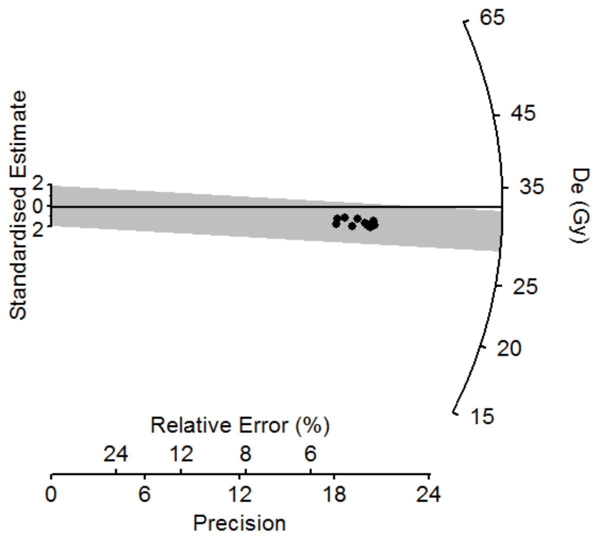

Dose recovery ratio L-EVA-1430 0.92

Figure S14. Quartz dose recovery test results for L-EVA-1430 (quartz; Unit 1), illustrated as a radial plots. The black line corresponds to the administered dose and the shaded lines to 2σ on either side of the central age.

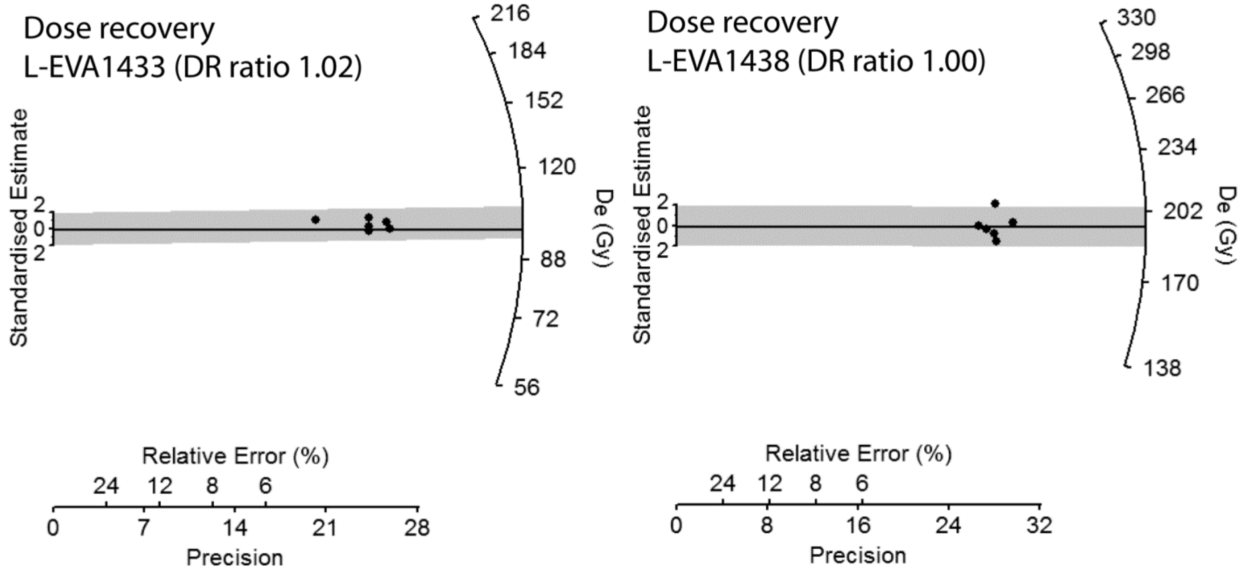

Figure S15. Polymineral dose recovery test results for L-EVA1433 (Unit 2-3) and L-EVA1438 (Unit 4-3), illustrated as radial plots. The black lines correspond to the administered dose and the shaded lines to 2σ on either side of the central age.

The resulting D_e values from both quartz and polymineral samples form broadly Gaussian distributions (Figures S16-S19). Therefore the Central Age Model (CAM) was used to calculate the equivalent dose for each sample (20).

L-EVA1430: 37.1 ± 0.5 Gy

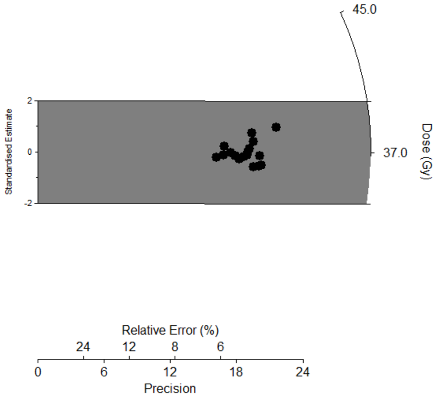

L-EVA1431: 44.4 ± 1.5 Gy

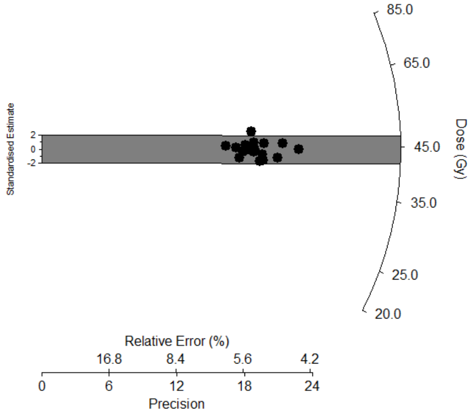

L-EVA1432: 56.0 ± 1.1 Gy

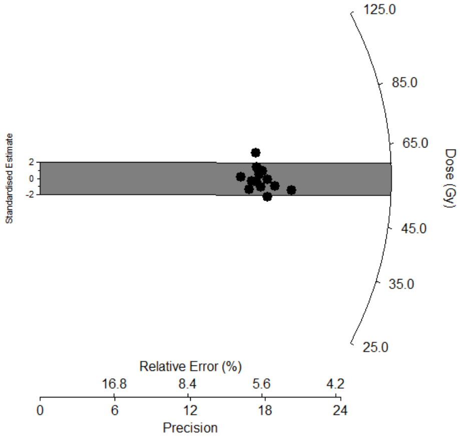

L-EVA1433: 64.7 ± 1.5 Gy

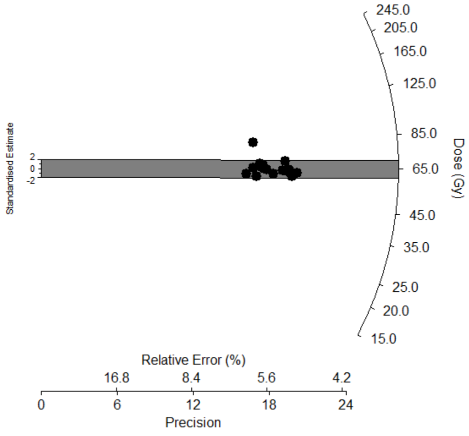

L-EVA1435: 61.8 ± 0.9 Gy

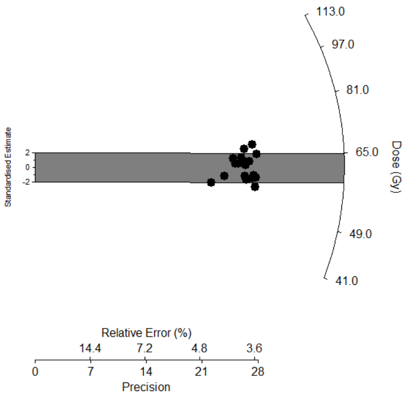

L-EVA1436: 72.3 ± 0.7 Gy

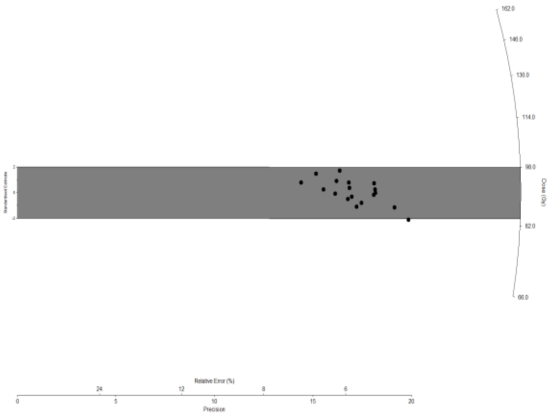

Figure S16. Equivalent dose distributions for quartz samples L-EVA1430-1433, 1435-1436 from Tolbor-16.

L-EVA1437: 90.8 ± 1.3 Gy

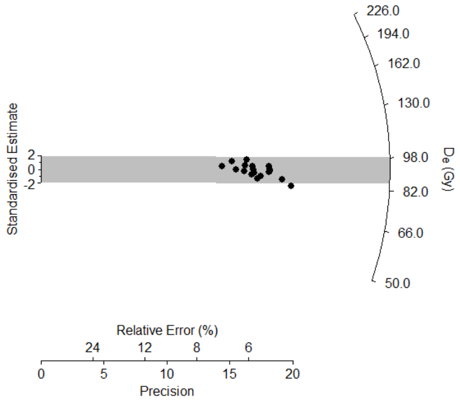

L-EVA1438: 92.1 ± 1.3 Gy

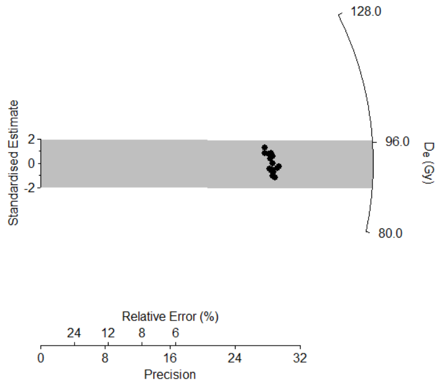

L-EVA1441: 104 ± 2 Gy

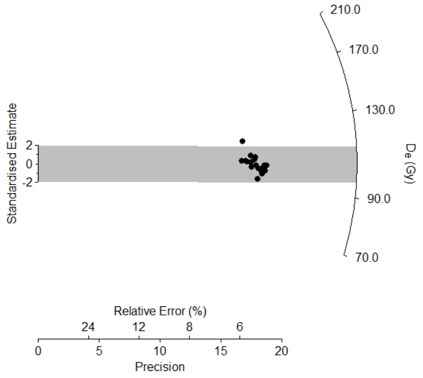

L-EVA1443: 40.1 ± 0.4 Gy

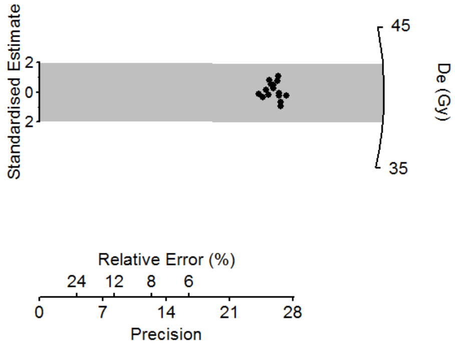

L-EVA1444: 52.5 ± 0.8 Gy

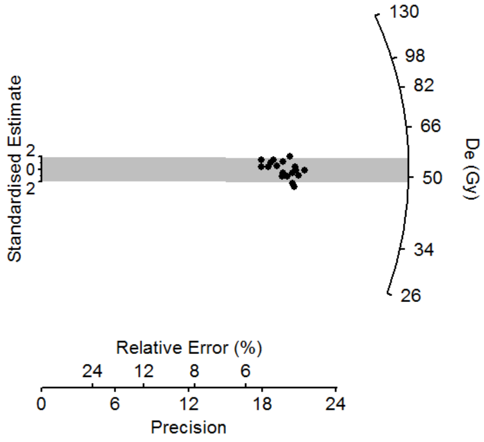

L-EVA1445: 86.1 ± 1.2 Gy

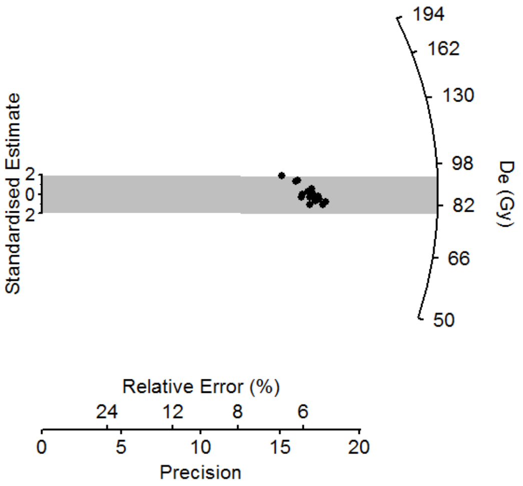

Figure S17. Equivalent dose distributions for quartz samples L-EVA1437-1438, 1441, 1443-1445 from Tolbor-16.

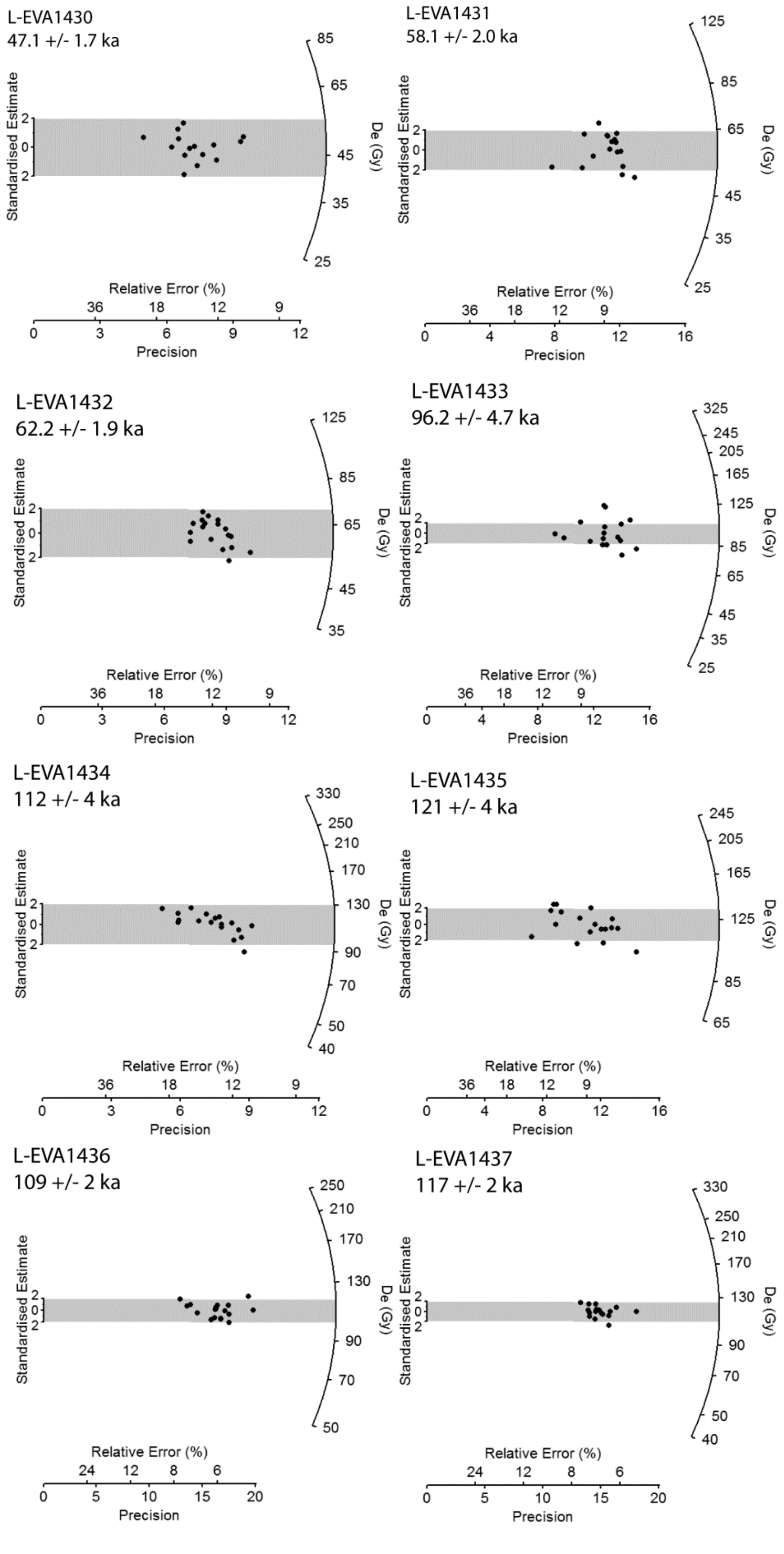

Figure S18. Equivalent dose distributions for polymineral samples L-EVA1430-1437 from Tolbor-16.

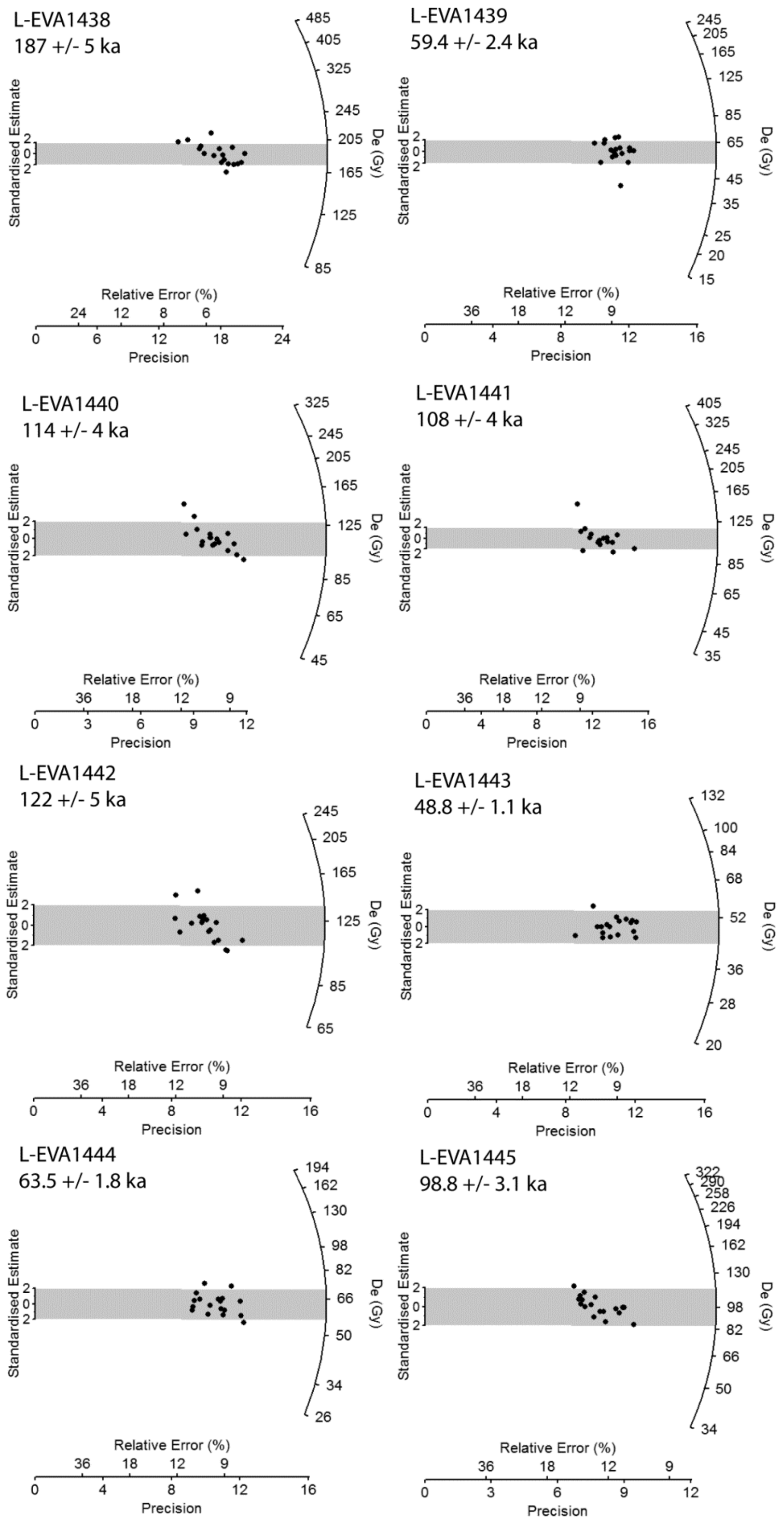

Figure S19. Equivalent dose distributions for polymineral samples L-EVA1438-1445 from Tolbor-16.

3.4. Dose rate calculations

Dose rates were determined based on high resolution germanium gamma spectrometric analysis of the radioactivity of uranium, thorium, potassium, and their daughter isotopes, undertaken on the bulk sediment samples at the "Felsenkeller" laboratory (VKTA) in Dresden. Gamma dose rates were calculated using published attenuation factors (24). The beta component of the dose rate was measured using an in-house beta counter from Risø. Dose rate attenuation by moisture was accounted for using average measured water content values of each sample, and incorporating an uncertainty value reflecting the saturation potential of the sediments based on laboratory measurements (10 ± 5%). The cosmic ray component of the dose rate was calculated based on published formulae (25).

3.5. Age calculations

The age calculations are summarized in Table S3. Alpha-values of 4 ± 2% and 8 ± 2% (26, 27) were used to correct for internal dosimetry within the quartz and polymineral fine grains, respectively.

Table S3. Equivalent dose (D_e), dose rate data and quartz OSL (upper values, italics) and pIR-IRSL$_{290}$ polymineral (lower values, plain text) age estimates for Tolbor-16. Dose rates are listed as attenuated. Moisture content is 10 ± 5% for all samples.

**Lab code**	**D_e (Gy)**	**Dose rates (Gy/ka)**				**Age (ka)**
		**γ**	**β**	**Cosmic**	**Total**	
			**Pit 1**			
L-EVA1430	*37.1 ± 0.5*	0.97 ± 0.10	1.84 ± 0.18	0.21 ± 0.02	*3.36 ± 0.30*	***11.1 ± 1.0***
	47.1 ± 1.7				3.69 ± 0.31	12.8 ± 1.2
L-EVA1431	*44.4 ± 1.5*	0.89 ± 0.09	1.64 ± 0.16	0.21 ± 0.02	*3.06 ± 0.28*	***14.5 ± 1.4***
	58.1 ± 2.0				3.39 ± 0.30	17.2 ± 1.6
L-EVA1432	*56.0 ± 1.1*	0.88 ± 0.09	1.62 ± 0.16	0.20 ± 0.02	*3.03 ± 0.30*	***18.5 ± 1.9***
	62.2 ±1.9				3.35 ± 0.31	18.6 ± 1.8
L-EVA1433	*64.7 ± 1.5*	0.90 ± 0.09	1.52 ± 0.15	0.20 ± 0.02	*2.97 ± 0.28*	***21.8 ± 2.1***
	96.2 ± 4.7				3.32 ± 0.29	29.0 ± 2.9
L-EVA1434	112 ± 4	0.86 ± 0.09	1.45 ± 0.15	0.19 ± 0.02	3.17 ± 0.29	35.3 ± 3.5
L-EVA1435	*61.8 ± 0.9*	0.80 ± 0.08	1.37 ± 0.14	0.19 ± 0.02	*2.84 ± 0.28*	***21.8 ± 2.2***
	121 ± 4				3.08 ± 0.28	39.3 ± 3.8
L-EVA1436	*72.3 ± 0.7*	0.80 ± 0.08	1.45 ± 0.15	0.18 ± 0.02	*2.72 ± 0.27*	***26.6 ± 2.6***
	109 ± 2				3.09 ± 0.27	35.3 ± 3.2
L-EVA1437	*90.8 ± 1.3*	0.82 ± 0.08	1.53 ± 0.15	0.18 ± 0.02	*2.76 ± 0.26*	***32.9 ± 3.13***
	117 ± 2				3.14 ± 0.26	37.3 ± 3.2
L-EVA1438	*92.1 ± 0.8*	0.84 ± 0.08	1.51 ± 0.15	0.17 ± 0.02	*2.83 ± 0.25*	***32.6 ±2.9***
	187 ± 5				3.18 ± 0.27	58.8 ± 5.3

			**Pit 3**			
L-EVA1439	59.4 ± 2.4	0.81 ± 0.08	1.53 ± 0.15	0.22 ± 0.02	3.13 ± 0.25	19.0 ± 1.7
L-EVA1440	114 ± 4	0.86 ± 0.09	1.65 ± 0.16	0.21 ± 0.02	3.39 ± 0.28	33.6 ± 3.0
L-EVA1441	*104 ± 2*	0.87 ± 0.09	1.54 ± 0.15	0.21 ± 0.02	*2.85 ± 0.24*	***36.5 ± 3.4***
	108 ± 4				3.33 ± 0.29	32.4 ± 3.1
L-EVA1442	122 ± 5	0.93 ± 0.09	1.56 ± 0.16	0.20 ± 0.02	3.49 ± 0.32	34.9 ± 3.5
			**Pit 2**			
L-EVA1443	*40.1 ± 0.4*	0.85 ± 0.09	1.57 ± 0.16	0.21 ± 0.02	*2.85 ± 0.24*	***14.1 ± 1.2***
	48.8 ± 1.1				3.22 ± 0.26	15.2 ± 1.3
L-EVA1444	*52.5 ± 0.8*	0.81 ± 0.08	1.61 ± 0.16	0.20 ± 0.02	*3.06 ± 0.27*	***17.2 ± 1.5***
	63.5 ± 1.8				3.15 ± 0.25	20.2 ± 1.7
L-EVA1445	*86.1 ± 1.2*	0.75 ± 0.08	1.56 ± 0.16	0.18 ± 0.02	*2.98 ± 0.28*	***28.9 ± 2.7***
	98.8 ± 3.1				3.02 ± 0.24	32.8 ± 2.8

The quartz and polymineral ages generally lie within 2σ error of one another, which supports their accuracy. In one instance, the polymineral age differed from the quartz age by more than 2σ (sample 3071/ L-EVA-1439, Pit 3). In this case, the quartz age was assumed to be the more likely accurate value. However, since this sample was collected from an archaeologically sterile unit and falls into stratigraphic order, the age of this sample has little bearing on our overall interpretation of the chronology of occupation of the Tolbor 16 site.

Table S4. Summary

Pit	Level (Unit)	Sample Code	Field ID	OSL Age (poly)	2s Err	OSL Age (quartz)	2s Err
1	1	L-EVA1430	3044	12.8	2.4	11.1	2
1	2	L-EVA1431	3048	17.2	3.2	14.5	2.8
1	2	L-EVA1432	3052	18.6	3.6	18.5	3.8
1	2	L-EVA1433	3054	29	5.8	21.8	4.2
1	3a	L-EVA1434	3057	35.3	7	\	\
1	3b	L-EVA1435	3062	39.3	7.4	21.8	4.4
1	3c	L-EVA1436	3065	35.3	6.4	26.6	5.2
1	3d	L-EVA1437	3046	37.3	6.4	32.9	6.4
1	3d	L-EVA1438	3050	58.8	10.6	32.6	5.8
2	2	L-EVA1443	3080	15.2	2.6	14.1	2.4
2	2	L-EVA1444	3082	20.2	3.4	17.2	3
2	2\3	L-EVA1445	3084	32.8	5.6	28.9	5.4
3	2	L-EVA1439	3071	19	3.4	\	\
3	2\3	L-EVA1440	3073	33.6	6	\	\
3	3	L-EVA1441	3075	32.4	6.2	36.5	6.8
3	3	L-EVA1442	3077	34.9	7	\	\

4. Radiocarbon dating: sample selection, pretreatment, dating, calibration and modelling

4.1 Samples selection and location

In Tolbor-16, most of the cortical surface of the bones is gone. Hence, it was not possible to identify anthropogenic or animal post-depositional modifications (e.g. cut marks). Overall, 20 % (N=13) of the bone fragments larger than 2cm have been dated. Our selection of samples was limited by the preservation issues and by the complexity of the stratigraphy. In Pit 4, MAMS-31815 was collected at the interface of unit 2 and unit 3. Although there are possible intrusions of the material from unit 3 (Figure 1), we note that the age is consistent with the dates obtained on samples MAMS-24089 and MAMS-24090 but also with OSL age-estimates for the top of 3a in Pit 1 and the unit 2/3 interface in Pit 2 and Pit 3 (SI3). MAMS-240873 is associated with 3b and at 95.4% confidence interval; it is consistent with OSL measurements from unit 3 polymineral samples. The same applies to the rest of the samples collected in solifluction lobe 3c. The ages obtained for the Pit 4 –unit 3c (archaeological component referred to as IUP) is further confirmed by the sample MAMS-20981, obtained in unit 3c in Pit 1. It is associated with the same archaeological material stratigraphically below a thick white lamina.

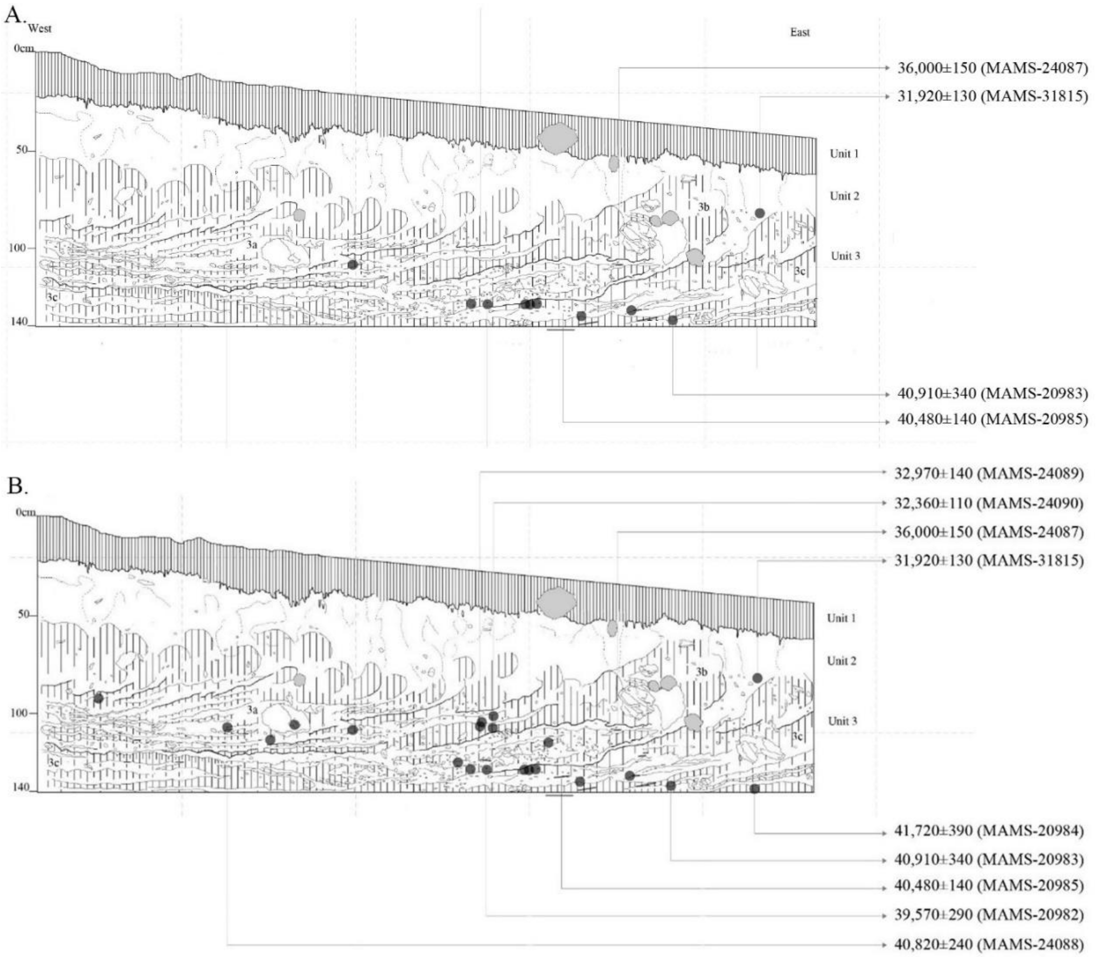

Figure S20: Pit 4, projection of piece-plotted bones and bone fragments on the North wall. A. 1m projection, B., 3m projection.

4.2 Pre-treatment and dating

Samples were selected for dating when showing a satisfying amount of collagen yield (>1%) (Ambrose, 1990; Weber et al., 2005; Hublin et al., 2012) and C:N between 2.9 and 3.5 (Klinken, 1999). The samples were pretreated at MPI-EVA Leipzig using the method described in Talamo and Richards (2011). Approximately 500 mg of samples were first cleaned and then demineralized in 0.5 M HCl at room temperature until no CO_2 effervescence could be observed. 0.1 M NaOH were then added for 30 min to remove humics. The NaOH step was followed by further rinsing with 0.5 M HCl for 15 min. The sample was then gelatinized, following Longin (1971), in a pH3 solution at 75 C for 20 h. The resulting gelatin was first filtered in an Eeze-Filter™ (Elkay Laboratory Products (UK) Ltd.) to remove small (<8 mm) particles and then through a 30 kDa ultrafilter (Sartorius “Vivaspin 15”) (Brown et al., 1988). Prior to use, the filter was cleaned to remove carbon containing humectants (Brock et al., 2007). The sample was then lyophilized for 48 h. The selected samples were dated by AMS at the Klaus-Tschira-Labor für Physikalische Altersbestimmung (Curt-Engelhorn- Zentrums für Arch€aometrie), Mannheim, Germany (Kromer et al., 2013).

Table S4: Radiocarbon dates, isotopic values, % of collagen and C:N ratios of Tolbor-16. The results of AMS radiocarbon dating of 13 samples from Tolbor-16. And the respective amount of collagen extracted (%Coll, >30 kDa fraction) and C:N ratios

**MPI Code**	**Level**	**Cult. Layer**	**% Coll**	**C:N**	**AMS Nr**	**^{14}C Age**	**1s Err**	**Z**
S-EVA 24026	Pit 1-Unit 2	AH2	1,99	3,3	MAMS-14938	15660	40	99,456
S-EVA 28443	Pit 1-Unit 3a	AH3	11,48	3,1	MAMS-20980	32910	160	99,303
S-EVA 24020	Pit 1-Unit 3a	AH4	2,82	3,3	MAMS-14932	33320	180	98,978
S-EVA 28442	Pit 1-Unit 3a	AH4	12,69	3,1	MAMS-20979	33520	170	99,329
S-EVA 28444	Pit 1-Unit 3c	AH6	10,81	3,1	MAMS-20981	41030	350	98,942
S-EVA 31445	Pit 4-Unit 2\3a	AH3	11,42	3,2	MAMS-24089	32970	140	100,04
S-EVA 31446	Pit 4-Unit 2\3a	AH3	10,66	3,2	MAMS-24090	32360	110	100,1
R-EVA 1899	Pi4-Unit 2\3a	AH3	11	3,2	MAMS-31815	31920	130	100,06
S-EVA 31442	Pit 4-Unit 3b	AH5	10,69	3,2	MAMS-24087	36000	150	100,02
S-EVA 28445	Pit 4-Unit 3c	AH6	8,26	3,3	MAMS-20982	39570	290	99,793
S-EVA 28448	Pit 4-Unit 3c	AH6	12,63	3,2	MAMS-20985	40480	320	99,595
S-EVA 31444	Pit 4-Unit 3c	AH6	13,46	3,2	MAMS-24088	40820	240	100,03
S-EVA 28446	Pit 4-Unit 3c	AH6	11,03	3,2	MAMS-20983	40910	340	99,715
S-EVA 28447	Pit 4-Unit 3c	AH6	10,85	2,7	MAMS-20984	41720	390	99,698

Table S5: Calibrated AMS 14C dating of Tolbor (unmodeled). Modelled calibrated boundaries and ages of the site performed using OxCal 4.3 (37)

	**Unmodelled (BP)**				**Modelled (BP)**			
Amodel 92.3	**from**	**to**	**from**	**to**	**from**	**to**	**from**	**to**
Aoverall 92.1	68,20%		95,40%		68,20%		95,40%	
Ust-Ishim Weighted mean (41410 960)	45770	44010	46880	43210	45790	44010	46900	43220
**Tolbor**								
**End Unit 2/3a**					**36060**	**35090**	**36240**	**33230**
MAMS-24089 (32970 140)	37300	36640	37680	36460	37140	36530	37590	36370
MAMS-31815 (31920 130)	36030	35650	36190	35480	36100	35720	36240	35530
MAMS-24090 (32360 110)	36390	36110	36540	35930	36390	36110	36550	35940
**Transition Unit 3b/Unit 2/3a**					**38380**	**36590**	**40290**	**36470**
MAMS-24087 (36000 150)	40890	40410	41110	40200	40890	40410	41120	40190
**Transition Unit 3c/Unit 3b**					**43780**	**42480**	**44210**	**41190**
MAMS-20985 (40480 320)	44400	43700	44710	43370	44410	43740	44710	43410
MAMS-20984 (41720 390)	45490	44770	45850	44420	45160	44450	45560	44130
MAMS-20983 (40910 340)	44810	44110	45140	43730	44730	44090	45040	43720
MAMS-20982 (39570 290)	43500	42970	43870	42760	43790	43060	44240	42850
MAMS-24088 (40820 240)	44660	44110	44920	43800	44620	44090	44880	43800
**Start Unit 3c**					**45590**	**44570**	**46690**	**44220**

4.3 Modelled age estimates:

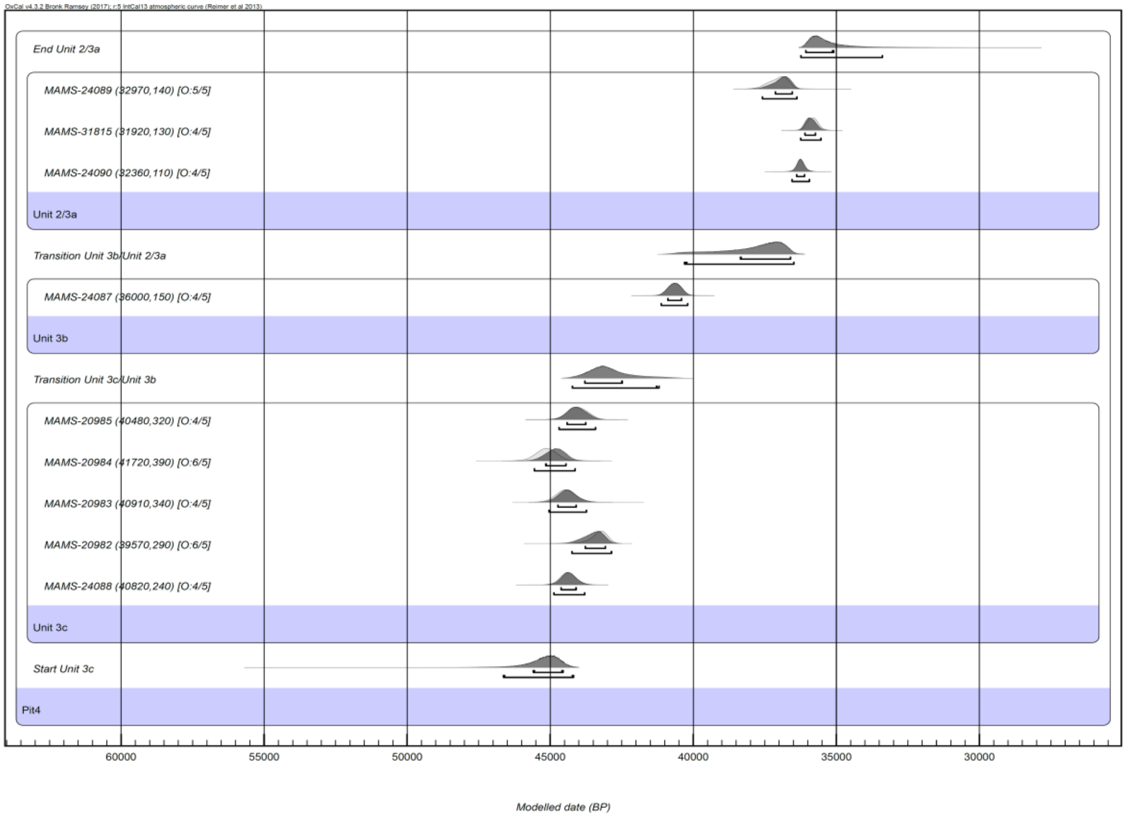

Figure S21: Tolbor-16, Pit 4, modelled age estimates with boundaries calculated from an outlier model (38) in OxCal 4.3 (37)

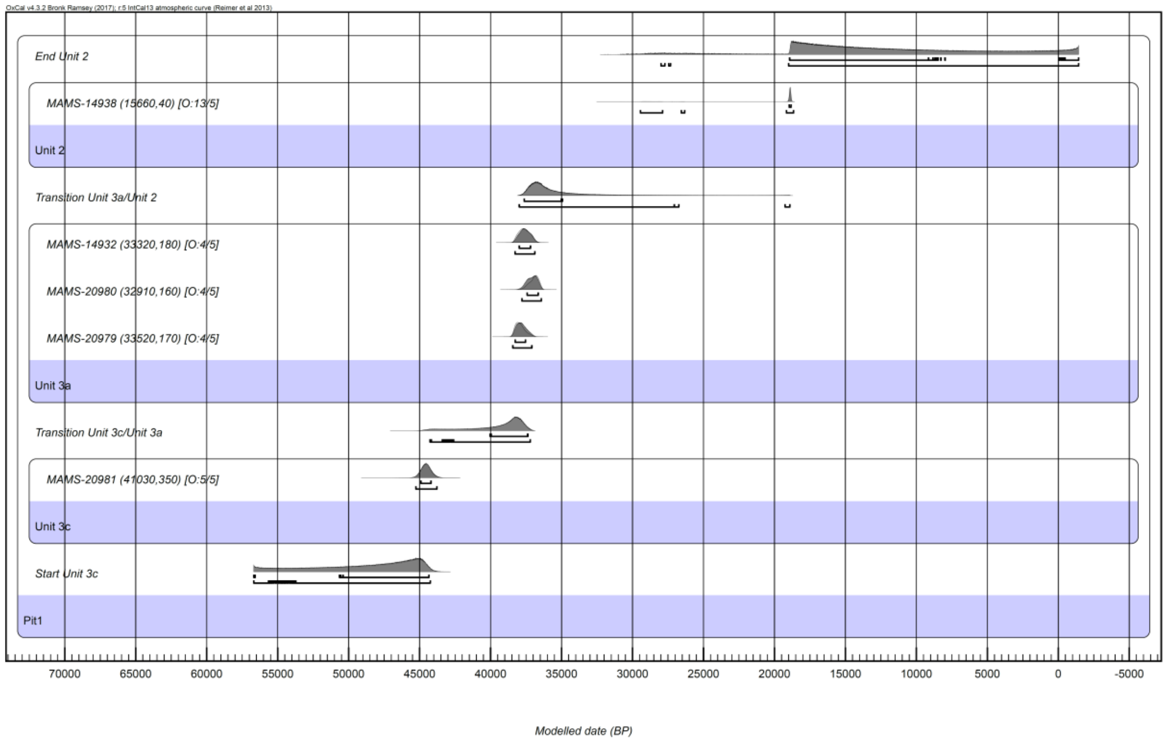

Figure S22: Tolbor-16, Pit 1, modelled age estimates with boundaries calculated from an outlier model (38) in OxCal 4.3 (37)

5. AH6 Lithic assemblage: sampling, fabric, technology and implications

5.1. Spatial distribution and sampling procedure

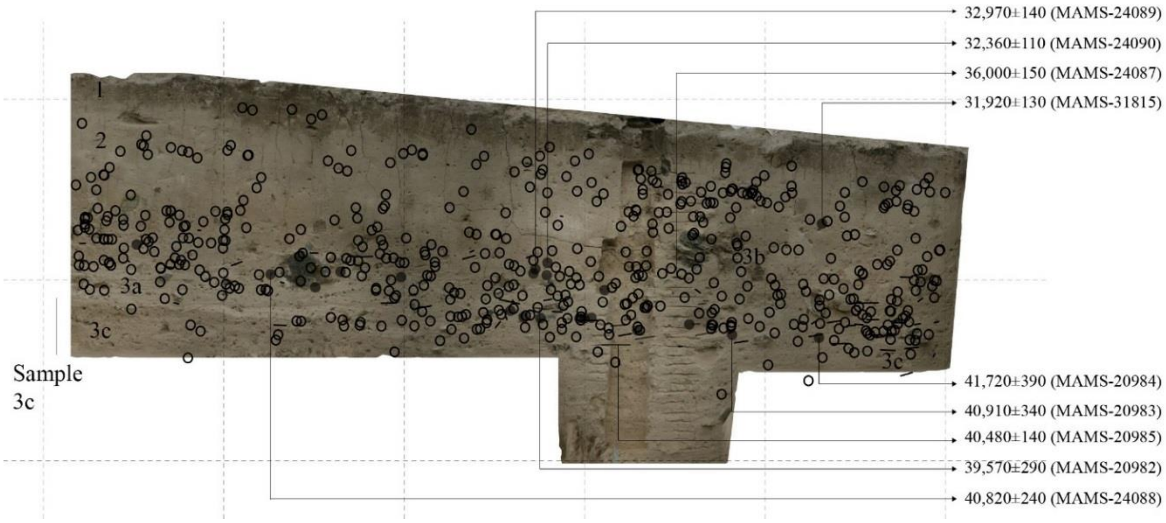

Figure S23: projection on North Wall of excavation Pit 4. Hollow dots are lithic artefacts (1m projection), black dots are bones (3m projection). Lines are 2-shots artefacts, bold lines are 2-shots bones

Although all geological evidence points to low-energy deposition, slopewash, shearing or doubling of sediments may have altered the spatial organization of the remains. Erosion and slope wash may have led to depleted occupation horizons or alternatively, post-depositional processes may artificially inflate the thickness of the archaeological layer (time averaging). Such a situation is best illustrated by the eastern end of the north section where the assemblage at the interface of sedimentary units 2 and 3 sits directly on the top of the assemblage of unit 3c (Fig. S23). Because the solifluction challenges the laws of superposition, we sampled the lithic material that is clearly attributed to solifluction lobe 3c. AH6 corresponds to the lowest archaeological deposit in an excavated surface of 15 m^2. To draw the upper limit of lobe 3c, we used observations made during the excavations and projection of the piece-plotted material on to the western and northern walls. In the western wall, lobes 3a and 3c dip northward; hence, vertical projections on a wall from a greater distance than 1m are likely to be inaccurate. Instead, we used 3D projections of piece-plotted artefacts (<2cm) on a 3D model of Pit 4 generated by photogrammetry. Fig. S24A illustrates the distribution of the AH6 and the distribution of AH1-AH5. Fig. S24B shows that bone distribution is in close association with AH6, artefact from AH6 and the sediment bulk samples. Note that bone fragments are in close association with AH6 and AH5.

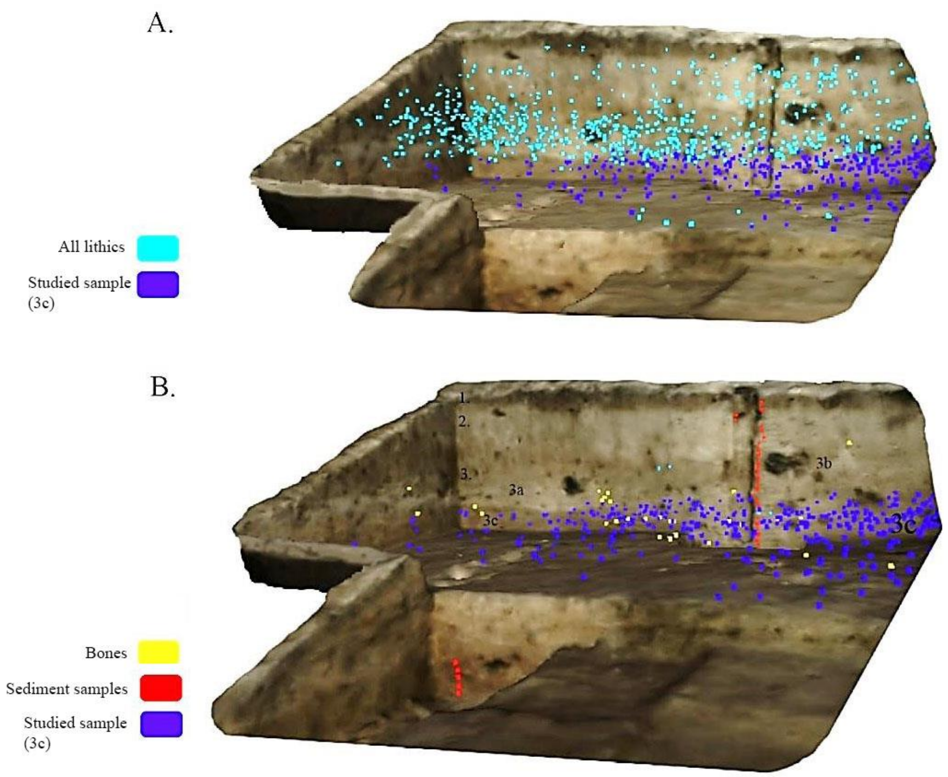

Figure S24: 3D models of the Western and Northern wall on Pit 4 with projection of piece-plotted objects (<2cm, here excluding the two-points). A. Piece-plotted artefacts from AH1-AH5 (light blue) and the sample studied here, AH6 (dark blue) in solifluction lobe c, Unit 3. B. Piece-plotted artefacts from AH6 (dark blue), piece plotted bones (yellow) and bulk sediment samples (red). Geo-localized 3D photogrammetry in Agisoft Photoscan, data collected in New Plot (8, 9) 3D plot generated in ISTI-CNR MeshLab.

5.2 Fabric analyses

We performed a standard fabric analysis (39, 40). ‘Orientation of clast’ refers to an alignment of an elongated object in three-dimensional space in terms of horizontal and vertical displacement. ‘Bearing’ refers to a horizontal angle of object’s long axis relative to the arbitrary north. ‘Plunge’ or dip is a vertical angle relative to a horizontal plane. Rose and Schmidt diagrams best represent these data (Fig. S25). Statistical procedure for orientation data applies uniformity tests for bearing and plunge values. Bearing data are captured in 360 degrees so the use of circular instead of linear statistics is more appropriate (e.g. Rayleigh test of uniformity). Plunge, on the other hand, follows regular statistical testing (e.g. t-test). If uniformity is confirmed for bearing angles, a preferential orientation of clasts is indicated suggesting post-depositional processes that could have aligned the objects. On the other hand, a non-random pattern of plunge values suggest that object dip angles correspond to the surface on which they were deposited and didn’t go through significant disturbance.

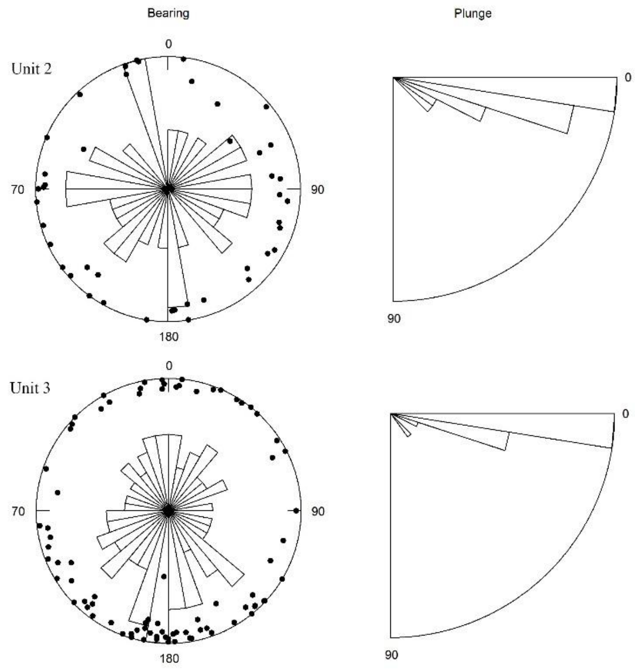

Figure S25: Schmidt diagram and plunge for Unit 2 (above) and Unit 3c (below)

Results are presented on a Benn diagram (41) that considers both bearing and plunge angles simultaneously. Following Benn's method, Lenoble and Bertran (40) provided a comparative data from experimentally and archaeologically derived data for debris flow, runoff on steep and shallow slopes, and solifluction. The range of Benn values for these processes are used as a reference.

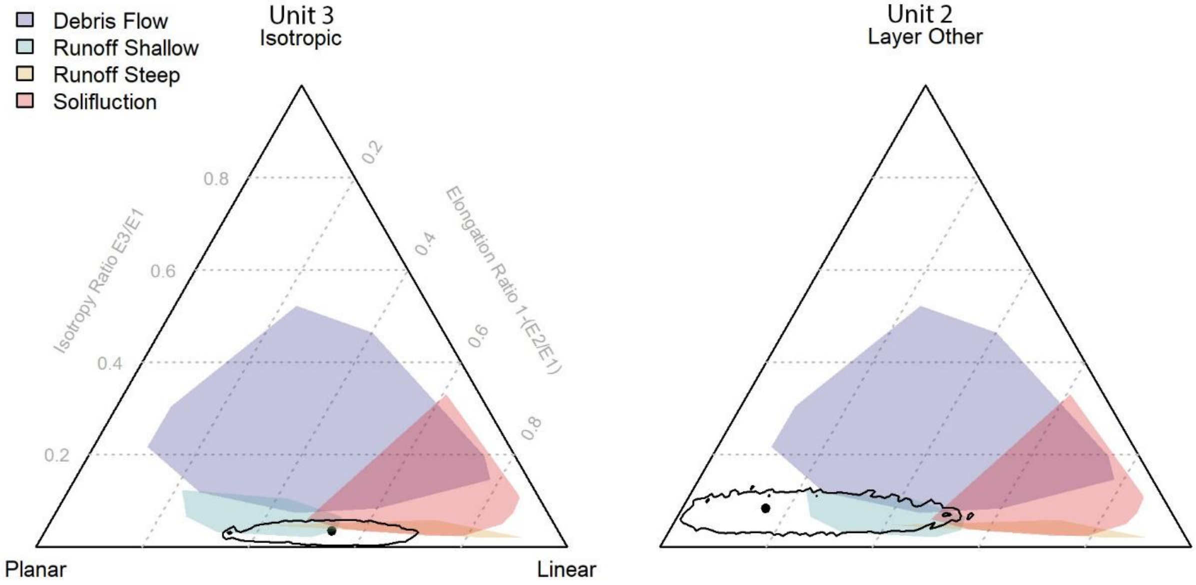

Figure S26. Benn-diagram for unit 3 (left) and unit 2 (right).

We recorded two points for elongated with the total station (42). Due to scarcity of bone remains and natural clasts, two points are mainly recorded for elongated lithic artefacts (blades). Pit 4 provided a sample of 83 objects for unit 3 and 45 objects for unit 2. To reach an acceptable sample size (ca. 50) (39, 43, 44) in Pit 4, we considered Unit 3 as one single unit (lumping 3a, 3b and 3c) and we used Unit 2 as a control sample. Analyses and figures have been produced in R (45) using the protocols and the codes described by McPherron (44).

Unit 3 shows overlap with three processes: runoff, both shallow and steep, and solifluction, while unit 2 plots in a planar portion of Benn diagram (Figure S26). Permutation test is used to assess whether two assemblages have different orientations. This test allows for assessing and analyzing orientations in three-dimensional space (treated together as a vector that describes the orientation of an object) rather than bearing and plunge aspects separately (44). The test assesses the probability that two assemblages come from the same population (the null that they have the same orientation cannot be rejected). Permutation test between Benn ratios of unit 3 and unit 2, based on 10000 resampling, shows that we cannot reject the null hypothesis ($p = .92$) that the samples are the same. This result likely reflects the overlap between confidence intervals in the planar range of the diagram. Overall, the results are in line with the geological descriptions (SI section 2) that noted the role of run-off in the formation of unit 3 and identified post-depositional events such as solifluction (3a-3c). We interpret the large standard deviation from Unit 2 as reflecting the impact of an interface unit 2/unit 3 (which features solifluction and intrusion of material from unit 3 into unit 2) on the overall fabric signatures. We also note that by lumping together the 3 solifluction lobes into one unit, we may have increased the standard deviation of unit 3, hence resulting in the slight overlap observed between the two units.

5.3 Technological description

The lithic assemblage is mainly produced on a medium- to fine-grain, local, cryptocrystalline raw material for which the primary sources occur in the whole valley as sub-vertical uplifted bands. Eroded clasts of this material along streams and canyons around the site, or buried under alluvium along the Tolbor River, constitute a possible secondary source. Although the use of fine-grained chert, flint or jasper-like material (possibly exotic) is reported during the later phases of the Upper Palaeolithic, it is absent in the coarse fraction of the AH6 sample (>2cm). A light blue patina, semi-extensive with root-like patterns, alters most of the archaeological material. On the opposite side of the artefacts, we note the frequent occurrence of calcium-carbonate concretions usually located on the lower face (in contact with the sediments). This phenomenon is consistent with the macro- and microscopic geological observations and supports the idea that the concretion formed by ground water exposure after the re-deposition by solifluction.

Table S6. Sample composition

	N	*f*
**Preform**	3	0.4%
**Core**	16	1.9%
**Blank**	196	23.8%
**Retouched Tool**	91	11.0%
**Shatter**	56	6.8%
**Screen (<20 mm)**	463	56.1%
**Total**	825	100.0%

Among piece-plotted blanks, flake and blade frequencies are balanced, with blades being slightly better represented (N=107; 55%). When calculating a minimum number of individuals (MNI= sum of platform blanks), blades frequency decreases sharply (N=42; 36%). The same way, retouched blades are prominent within the total sample (N=59; 65%) but less so in the MNI (N=25; 37% of flakes). The core to blank and tools ratio is 1/15, but the latter is strongly affected by the fragmentation of blades. Considering the MNIs for blades and tools on blades drops the ratio to 1/5. Retouched flake frequencies are low in the whole sample (N=32; 36%) but high in the MNI (N=21; 65%) reflecting the difficulty to differentiate flake fragments from shatters. The flake category is mainly composed of platform and complete flakes (N=74; 88%). Blades, retouched or not, are represented by 90% of fragments.

Table S7: blade and retouched (R) dorsal pattern

	**Blade**		**RBlade**		**MNI Blade**		**MNI RBlade**	
	N	*f*	N	*f*	N	*f*	N	*f*
**Unidirectional**	19	18.45%	9	14.75%	13	30.95%	7	26.92%
**Bidirectional**	18	17.48%	18	29.51%	6	14.29%	4	15.38%
**Crest**	1	0.97%	2	3.28%	1	2.38%	1	3.85%
**First crest**	2	1.94%	0	0.00%	0	0.00%	0	0.00%
**Second crest**	8	7.77%	9	14.75%	6	14.29%	5	19.23%
**Neo crest**	3	2.91%	1	1.64%	0	0.00%	1	3.85%
**Cortical**	13	12.62%	4	6.56%	4	9.52%	1	3.85%
**Undetermined**	39	37.86%	18	29.51%	12	28.57%	7	26.92%
**Total**	103	100.00%	61	100.00%	42	100.00%	38	100.00%

The general structure of the blade assemblage is presented in Table S6; we highlight several features relevant to the present case. First, the blade dorsal faces include both unidirectional and bidirectional patterns. Explaining the variations in frequencies between these two categories is beyond the scope of this paper, but it is notable that the high degree of fragmentation influences the visibility of the dorsal patterns; hence there are numerous undetermined specimens. In general terms, the co-existence of bi-and unidirectional dorsal patterns suggests a core reduction system involving two-opposed platforms and alternate short sequences of flaking (Table S7). When the whole sample is considered, bidirectional blades are particularly well-represented among the

retouched tools. Second, the good representation of crested blades demonstrates that this method is part of the behavioural repertoire. It also underlines efforts to produce blades as opposed to an opportunistic production and illustrates core-management operations. The lack of initial crests as opposed to the second crests could indicate the import of semi-prepared cores from an unknown distance (arguably, the initial flaking could occur not further than a few meters away). This corroborates the low frequency of primary or secondary cortex on both blade and flake blanks. Another observation is that all categories of blades are retouched – including technical elements such as crested blades. Looking at the advanced stage of exhaustion of blade cores, a ratio of 1/5 is too low, and part of the blade production might be missing. The frequent use of technical pieces as retouched tools corroborates the idea of an expedient use.

The platform preparations show substantial variations between object categories (Table S8). For blades, plain, facetted and dihedral convex platforms are the most common. With retouched blades, dihedral and faceting preparations are the most frequent whereas plain platforms rise among unretouched blades. With flakes have very little faceting and plain, or unprepared platforms are the most frequents. This is consistent with the little investment observed in the flake production and a more careful preparation of blade platforms, some of which will be retouched into formal tools.

Table S8. Platform preparation type

	**Blade**		**RBlade**		**Flake**		**RFlake**	
	N	*f*	N	*f*	N	*f*	N	*f*
**Plain**	17	40.48%	4	15.38%	52	70.27%	10	47.6%
**Facetted**	11	26.19%	7	26.92%	2	2.70%	0	0.0%
**Dihedral**	2	4.76%	3	11.54%	7	9.46%	3	14.3%
**Dihedral convex**	7	16.67%	7	26.92%	1	1.35%	2	9.5%
**Cortical**	1	2.38%	1	3.85%	5	6.76%	1	4.8%
**Punctiform**	2	4.76%	1	3.85%	4	5.41%	2	9.5%
**Undetermined**	2	4.76%	3	11.54%	3	4.05%	3	14.3%
	42	100.00%	26	100.00%	125	100.00%	29	138.1%

The external platform angle (EPA) is prepared using various methods but notable are the marginal faceting and the pecking (Fig. S28-2), that occur in high frequencies in the Initial Upper Palaeolithic (IUP) from the Altai and the Baikal region (46, 47). Defined respectively as a faceting or a hammering of the platform edge, it is often combined with a prominent plain or dihedral platform. It mostly consists in preparation of the EPA (and not the platform per se) and therefore in Table S8, it is grouped here with the plain or dihedral platforms.

Table S9: platform thickness (in mm)

**Blade**		**RBlade**		**Flake**		**RFlake**	
μ	σ_X	μ	σ_X	μ	σ_X	μ	σ_X
5.4	2.9	7.1	3.4	5.5	2.6	4.4	1.7

Most platform thickness means are above 4mm, which for blades, would fall into the expected range of direct percussion with mineral hammer (Table S9) (48). Considering the large standard deviations and the lack of controlled experiments on the specifics of the raw material, direct percussion with hard hammer is not exclusive of the use of other techniques (e.g. soft stone hammer). The platform thickness of the retouched blades stands out as the thickest in the sample.

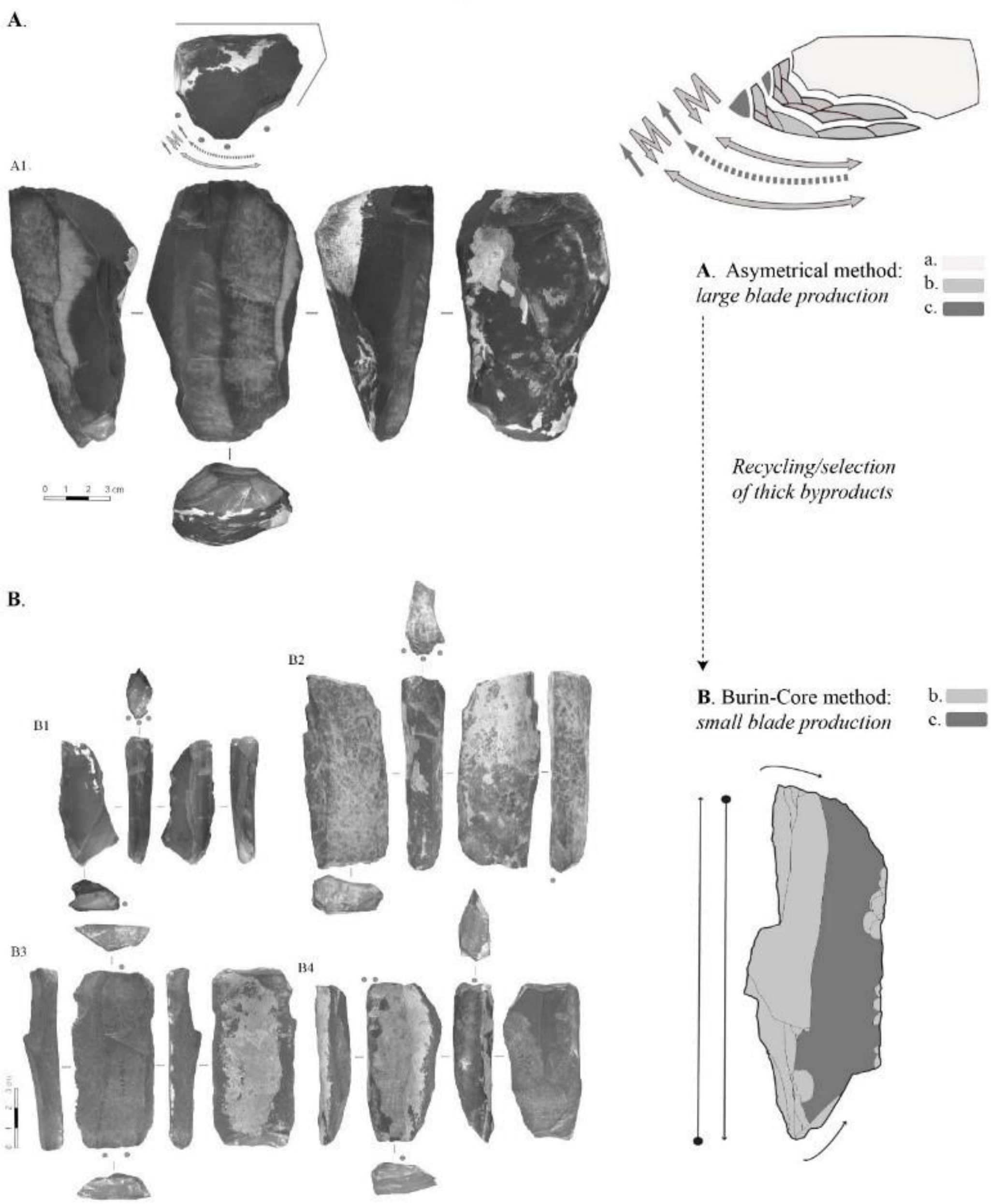

Figure S27: AH6, blade core reduction. Asymmetrical reduction pattern (A1) that produces large-medium size blades (Ab; Fig. S28-2, 3 and 5) and thick debordant/crests blades (Ac, Fig. S28-4). The latter (Ac) are snapped and turned into Burin-Cores (B1, B2, B4) or more rarely into truncated-facetted (B3).

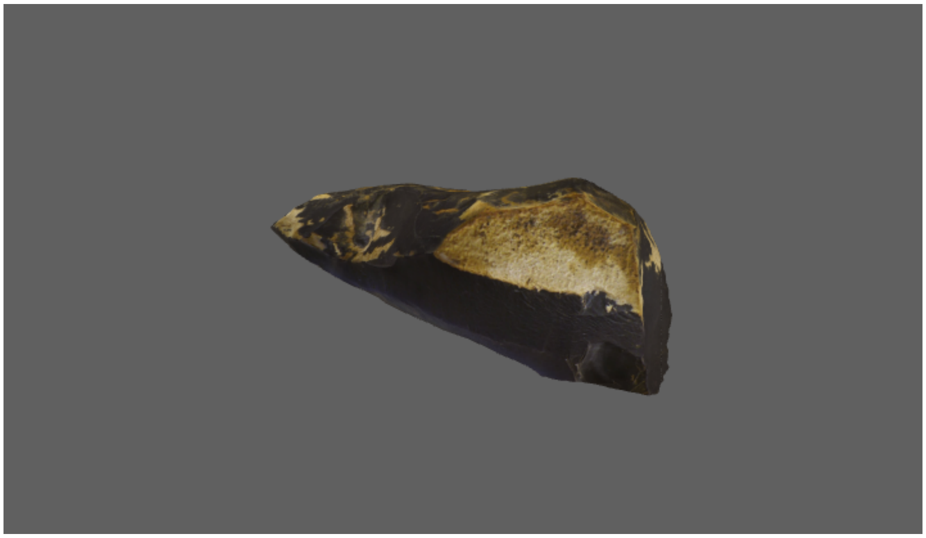

Figure S28: 3D model of Mode A core (Asymmetrical reduction)

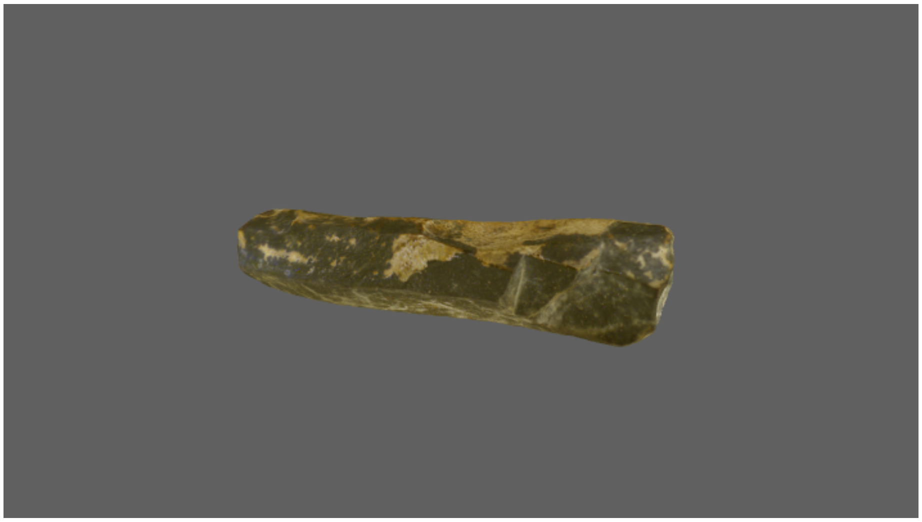

Figure S29: 3D model of Mode B core (Burin-Core)

Mode A cores (Fig. S27Aa) are reduced on both broad and narrow face (Fig. S27Ab) (Fig. S28), with neo-crest or thick *debordant* blades removed to manage lateral convexities (Fig. S27Ac). Mode A illustrates the production medium to large blade following a specific asymmetric pattern (49-51). These blades are of three different kinds based of their overall contour: with convergent (Fig. S30-2,3), sub-parallel (Fig. S30-5) and parallel edges (Fig. S30-2). Some of the thickest blades are further reduced using the Burin-Core method as defined by Zwyns et al. (52) (Fig.S29) 2). The latter show a flaking surface parallel to the long axis of the blade. Mode A cores are exhausted in different ways and at the end of the reduction process, they can either produce blades or flakes. It is worth noting that the method is relatively systematic and does not deviate until the end of the process. No specific production of flakes can be identified based on the cores and the production seems to be primarily oriented toward the production of blades.

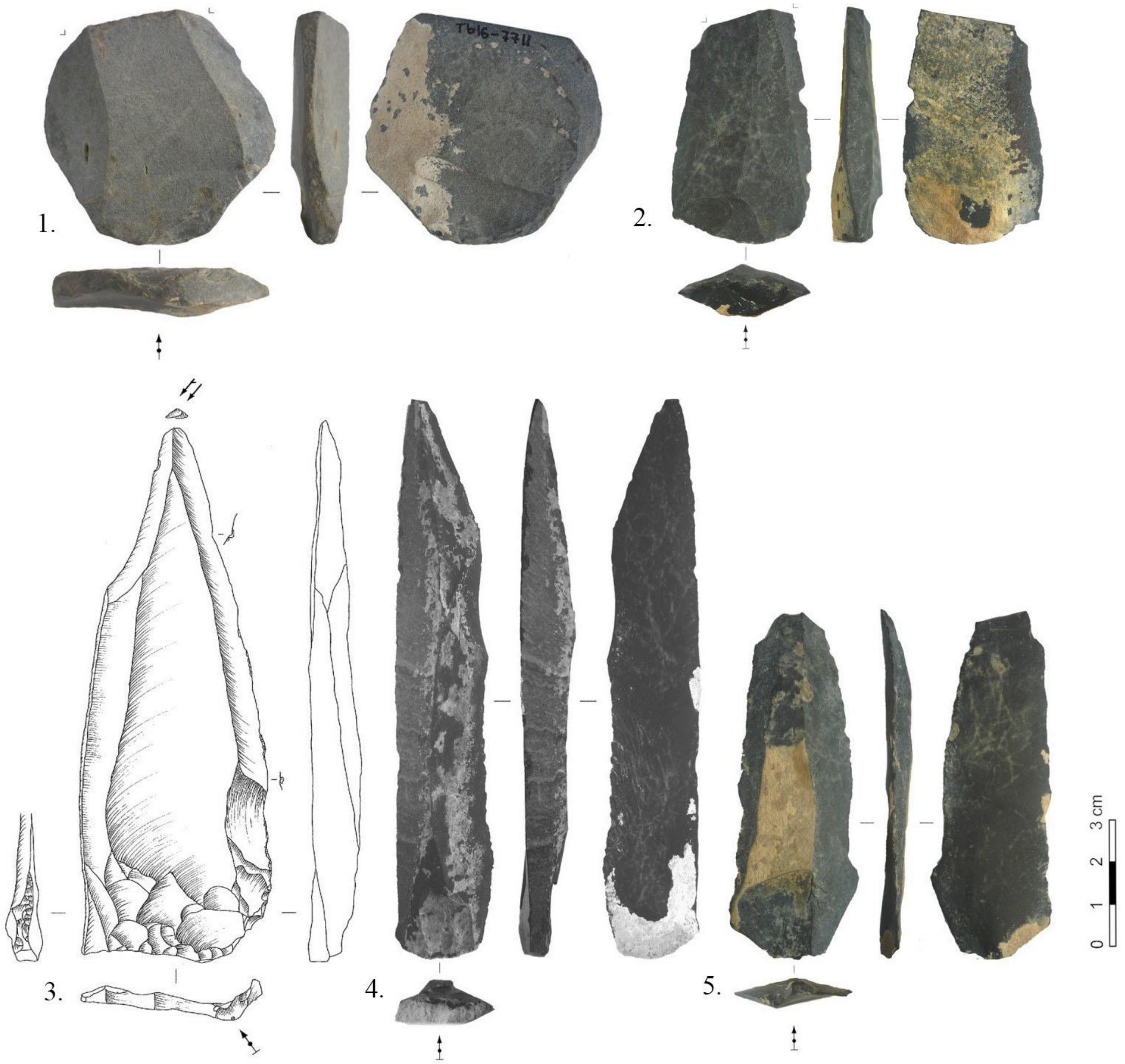

Figure S30: AH6, blade and tools. 1. Convergent laminar flake with basal thinning (inverse proximal removals - Kombewa). 2 and 3, convergent blades. 4 Debordant/naturally backed parallel blade. 5. Sub parallel blade with secondary cortex (rolled).

Half of the tools are retouched blades and retouched flakes and overall, there is a lack of formal tools. Among the standard types, notch and denticulate are the most numerous. The presence of Upper Palaeolithic tool types such as endscrapers and truncation is noted but except for a single dihedral burin, classic burin types are absent from the studied sample. This typological signature is typical of the IUP but it is notable that tools on flakes (including Middle Palaeolithic types such as notch and denticulate) are not produced on standardized blanks. Instead, they appear to be linked with management operations of Mode A blade core. These include core tablets and crest-shaping flakes. Anecdotally, the toolkit includes a small fragmented bifacial tool/preform.

Table S10. Retouched tool types

	**Blade**		**Flake**		**Total**	
	N	*f*	N	*f*	N	*f*
**Rblade**	41	67.2%	0	0.0%	41	44.1%
**Rflake**	0	0.0%	12	37.5%	12	12.9%
**Scraper**	0	0.0%	1	3.1%	1	1.1%
**Endscraper**	2	3.3%	2	6.3%	4	4.3%
**Burin**	1	1.6%	0	0.0%	1	1.1%
**Point**	2	3.3%	0	0.0%	2	2.2%
**Truncation**	2	3.3%	0	0.0%	2	2.2%
**Notch**	7	11.5%	9	28.1%	16	17.2%
**Denticulate**	6	9.8%	8	25.0%	14	15.1%
	61	100.0%	32	100.0%	93	100.0%

The assemblage AH6 from the Unit 3c is a blade-based assemblage. The main characters of the blade production fall into the definition of the Upper Palaeolithic (sub-volumetric reduction and volumetric reduction of the core, reliance on crest method, management of striking platform by core tablet removal). The technique used to produce blades likely involves a mineral hammer although this aspect of the blade technology would require further attention. Careful faceting of platforms and bevel-shaped EPA (marginal faceting) indicate an interest to produce robust blades whereas the alternate bidirectional system seems well adapted to the production of straight blanks. Mode A core shows a specific conception of the volume (50) described here as *asymmetrical reduction.*

The thickest blanks are selected for two distinct purposes. First, they are used as tools with expedient modifications. The selection of technical elements and the lack of formal blades, the exhaustion of the Mode A cores are elements indicative of a specific economic behaviour. Second, the thickest blades are further reduced using the Burin-Core method, or as truncated-facetted. There is no evidence for a specific production of flakes except for the exhausted blade cores (same reduction method, smaller size).

5.4 *Relevance for the regional record*

This exceptional combination of features assigns these assemblages to a united Initial Upper Palaeolithic (IUP) techno-complex and not only to a generic Upper Palaeolithic classification. Both Mode A and Mode B methods are documented in the Altai and in the Baikal regions and are typical

of the Asian IUP (48, 51, 53) but seem absent from the European variants such as the Bohunician (54, 55).

In the Altai, the IUP has been recognized at Ust-Karakol 1 (sector 1, OH5.5 and OH5.4), Kara-Tenesh, Maloyalomanskaya Cave (for a review see the Kara-Bom variant in 56). It can be traced eastward in the Cis-Baikal (e.g., Makarovo-4) (53, 57) Trans-Baikal (e.g. Khotyk, Kamenka A and C and Podzvonkaya) (58-60) Mongolia (e.g. Tolbor 4) (3, 6, 61) and China (Shuiddongou) (62-65). The IUP complex also marks the first appearance of the use of pigments, body ornaments, formal bone tools and even musical instruments in Siberia (53, 66). The quantity and quality of chronological data associated with the IUP vary depending on the region (for a review of the labs and methods see 67). Large standard deviation and occasional infinite ages are frequently associated with estimation close to the limit of the method. Younger results are not reported here as they may reflect differences between measurements (Conventional/AMS) or pre-treatment methods, contaminations by younger material. They could also indicate that in some regions the IUP may have lasted a few thousands of years.

Table S11. Chronological data of comparable IUP technologies

	**Layer**	**Material**	**^{14}C**	**sigma**	**calib. BP**	**sigma**	**Lab number**
**Kara Bom**	OH5-6	Charcoal	43200	1500	46931	1995	GX-17597
**Kara Bom**	OH5-6	Charcoal	43300	1600	47025	2052	GX-17596
**Kamenka**	A	Bone	40500	3800	44848	3587	AA-26743
**Khotyk**	3	Bone	38200	2800	42039	2598	AA-60267
**Podzvonkaya**	3	Bone	38900	3300	42755	3132	AA-26741
**Tolbor 4**	OH6	Bone	37400	2600	41354	2475	AA-79314

From the archaeological material, the IUP can be clearly distinguished from the Middle Palaeolithic assemblages from Siberia. From a chronological point of view, these two complexes overlap with the UI human remains. The Mousterian of the Sibiryachikha variant is defined based on two cave sequences, Okladnikov Cave and Chagyrskaya Cave (68, 69). Radiocarbon dates available for Okladnikov place the Mousterian occupation between 44,000 ± 4000 BP to 33,500 ± 700 BP suggesting that it overlaps with appearance of the IUP complex (70) whereas new results from Chagyrskaya would indicate an age beyond the limit of the radiocarbon range, perhaps during (OIS4-early OIS3). Both sequences are now clearly associated with easternmost Neanderthal human remains (71, 72).

In Siberia, the IUP lasted around 5,000 years before being replaced by a full-fledged Early Upper Palaeolithic (EUP) (51). The latter correspond to series of behavioural changes in terms of knapping techniques and methods. A notable switch toward the use of soft-hammer concomitantly occurs with a genuine bladelet production indicating a change in weaponry (composite spears) also observed in Europe around the same time. Although a possible continuity between IUP and EUP cannot be ruled out, it is generally assumed that these two complexes correspond to distinct human groups. As it is the case in Europe, the EUP would correspond to a permanent human settlement in Siberia.

6. ZooMS

6.1 Sample composition

In T16, there are few bones compared to the number of lithic artefacts. Bones are associated with the archaeology; where no artefacts are found bones are rare if present at all. When bones are preserved, they are fragmented, and the cortical surface is missing. The same situation is known at other sites in the valley and although it likely reflects preservation biases, the mechanism responsible for the decay of organic material is unknown.

Table S12: bone assemblage from Tolbor-16

	Bones >2cm		<2cm		Total	
	N	*f*	N	*f*	N	*f*
**T16**	68	100.0%	186	100.0%	254	100.0%
**Pit4**	31	45.6%	75	40.3%	106	41.7%
**Unit 3**	30	44.1%	49	26.3%	79	31.1%
**Unit 3c**	19	27.9%	49	26.3%	68	26.8%

6.2 Method

Hence, 24 fauna specimens were submitted for ZooMS analysis. ZooMS analysis was conducted using the ammonium-bicarbonate extraction protocol following Welker et al., (2016, PNAS). MALDI-TOF-MS analysis was conducted at the University of York in triplicate for each extract. Replicate spectra were merged for each sample, with taxonomic identification based on comparison with peptide marker mass lists for Eurasian fauna (73, 74).

In contrast to traditional morphological analysis, ZooMS analysis was successful for 23/24 studied specimens. One specimen could only be attributed to a lower taxonomic specificity due to poor collagen preservation (assigned to the family Bovidae, or reindeer). The other specimens comprise a variety of taxonomic clades but are dominated by *Bos*/*Bison* specimens. Despite the small sample size, we observe fauna components such as Felinae or Elephantidae, suggesting that the highly fragmented Late Pleistocene bone assemblages across the Eurasian steppe harbour a complexity which requires further biomolecular exploration.

6.3 Results

Table S13: results of the ZooMS analysis (all samples come from Pit4 except when indicated otherwise)

Tolbor_ZooMS	Code	S-EVA	Unit	ZooMS ID	Suggested Species
1	6732	31442	5	Bovidae/Reindeer	
2	2802	31443	3	Bos/Bison	Bos sp or Bison sp.
3		31444	3	Bos/Bison	Bos sp or Bison sp.
4	6281	31445	4/5	Elephantidae	Mammuthus sp.
5	6223	31446	2	Bos/Bison	Bos sp or Bison sp.
6	week 27/7 - 01/08		unknown	Bos/Bison	Bos sp or Bison sp.
7	Pit 5		Pit 5	Bos/Bison	Bos sp or Bison sp.
8	6160		4	Bos/Bison	Bos sp or Bison sp.
9	6243		5	Bos/Bison	Bos sp or Bison sp.
10	6630		3	Bos/Bison	Bos sp or Bison sp.
11	6629		3	Bos/Bison	Bos sp or Bison sp.
12	6610		3	Bos/Bison	Bos sp or Bison sp.
13	6553		3	Bos/Bison	Bos sp or Bison sp.
14	7893		3	Felinae*	Felis sp., Lynx sp.
15	6831		3	Bos/Bison	Bos sp or Bison sp.
16	6825		3	Caprinae*	Capra sp., Ovis sp., Ovibos sp.
17	6569		3	Equidae	Equus sp.
18	7876		3	Felinae*	Felis sp., Lynx sp.
19	6923		3	Bos/Bison	Bos sp or Bison sp.
20	6541		3	Bos/Bison	Bos sp or Bison sp.
21	6552		3	Bos/Bison	Bos sp or Bison sp.
22	6843		3	Bos/Bison	Bos sp or Bison sp.
23	6385		3	Bos/Bison	Bos sp or Bison sp.
24	6609		3	Equidae	Equus sp.

*****Asian representatives of the subfamilies Felinae and Caprinae are absent in the ZooMS peptide marker database. It is therefore difficult to provide more precise taxonomic identifications for these specimens.

References

(1) Gillam JC, Gladyshev SA, Tabarev AV, Olsen J 2012 Halfway to Mörön: shedding new light on Palaeolithic landscapes of northern Mongolia. *Legacy*, **16** (2): 14-17.

(2) Derevianko AP, Zenin AN, Rybin EP, Gladyshev, SA, Tsybankov AA, Olsen JW, Tseveendorj D, Gunchinsuren B (2007) The technology of early Upper Palaeolithic lithic reduction in Northern Mongolia: The Tolbor-4 site, *Archaeol Ethnol Anthropol Eurasia* **29**(1):16–38.

(3) Gladyshev SA, Olsen JW, Tabarev AV, Kuzmin YV (2010) Chronology and periodization of Upper Palaeolithic sites in Mongolia. *Archaeol Ethnol Anthropol Eurasia* **38**(3):33–40.

(4) Gladyshev SA, Olsen JW, Tabarev AV, Jull AJ (2012) The Upper Palaeolithic of Mongolia: Recent finds and new perspectives. *Quat Int* **281**: 36-46.

(5) Tabarev AV, Gunchinsuren B, Gillam JCh, Gladyshev SA, Dogandzic T, Zwyns N, Bolorbat Ts, Odsuren D (2012) Kompleks Pamiatnikov, Kamennogo Veka v Doline r.Ikh Tulberiin-Gol, Secevernaya Mongolia (razvedochniie rabotyi s ispolzovaniem GIC-Technnologii v 2011 g.). *Studia Archaeologica Insituti Archaeologici Academiae Scientiarum Mongolicae*, **32**(2): 26-43.

(6) Derevianko AP, Rybin EP, Gladyshev SA, Tsybankov AA, Gunchinsuren B, Olsen JW (2013) Early Upper Palaeolithic Stone Tool Technologies of Northern Mongolia: The Case of Tolbor-4 and Tolbor-15. *Archaeol Ethnol Anthropol Eurasia*, **41** (4): 21-37.

(7) Zwyns N, Gladyshev SA, Gunchinsuren B, Bolorbat T, Flas D, Dogandzic T, Gillam JC, Khatsenovich AM, McPherron SP, Paine CH, Purevjal KE, Stewart JR (2014) The open-air site of Tolbor 16 (Northern Mongolia): preliminary results and perspectives. *Quat Int* **347**: 53–65.

(8) McPherron S, Dibble H (2002) *Using Computers in Archaeology: A Practical Guide*. New York: McGraw Hill.

(9) www.oldstoneage.com/software

(10) Aitken MJ (1998) *An introduction to optical dating: the dating of Quaternary sediments by the use of photon-stimulated luminescence*. Oxford University Press, New York.

(11) Fitzsimmons KE, Iovita R, Sprafke T, Glantz M, Talamo S, Horton K, Beeton T, Alipova S, Bekseitov G, Ospanov Y, Deom JM, Sala R, Taimagambetov Z (2017) A chronological framework connecting the early Upper Palaeolithic across the Central Asian piedmont. *J Hum Evol* **113**: 107-126.

(12) Vandenberghe DAG, Flas D, De Dapper M, Van Nieuland J, Kolobova K, Pavlenok K, Islamov U, De Pelsmaeker E, Debeer AE, Buylaert JP (2014) Revisiting the Palaeolithic site of Kulbulak (Uzbekistan): First results from luminescence dating. *Quat Int* **324**:180-189.

(13) Buylaert JP, Jain M, Murray AS, Thomsen KJ, Thiel C, Sohbati R (2012) A robust feldspar luminescence dating method for Middle and Late Pleistocene sediments. *Boreas* **41**: 435-451.
(14) Fitzsimmons K, Hambach U, Veres D, Iovita R (2013) The Campanian Ignimbrite eruption: new data on volcanic ash dispersal and its potential impact on human evolution. *PLoS One* **8**: e65839.
(15) Frechen M, Schweitzer U, Zander A (1996) Improvements in sample preparation for the fine grain technique. *Ancient TL* **14**: 15-17.
(16) Timar A, Vandenberghe D, Panaiotu EC, Panaiotu CG, Necula C, Cosma C, van den haute P (2010) Optical dating of Romanian loess using fine-grained quartz. *Quat Geochron* **5**:143-148.
(17) Botter-Jensen L, Bulur E, Duller GAT, Murray AS (2000) Advances in luminescence instrument systems. *Radiation Measurements* **32**: 523-528.
(18) Murray AS, Wintle AG (2000) Luminescence dating of quartz using an improved single-aliquot regenerative-dose protocol. *Radiation Measurements* **32**:57-73.
(19) Murray AS, Wintle AG (2003) The single aliquot regenerative dose protocol: potential for improvements in reliability. *Radiation Measurements* **37**: 377-381.
(20) Galbraith RF, Roberts RG, Laslett GM, Yoshida H, Olley JM (1999) Optical dating of single and multiple grains of quartz from Jinmium rock shelter, northern Australia. Part 1, Experimental design and statistical models. *Archaeometry* **41**: 339-364.
(21) Jain M, Buylaert JP, Thomsen KJ, Murray AS (2015) Further investigations on ‘non-fading’ in K-Feldspar *Quat Int* **362**: 3-7.
(22) Thiel C, Buylaert JP, Murray A, Terhorst B, Hofer I, Tsukamoto S, Frechen M (2011) Luminescence dating of the Stratzing loess profile (Austria) - Testing the potential of an elevated temperature post-IR IRSL protocol. *Quat Int* **234**: 23-31.
(23) Thiel C, Horváth E, Frechen M (2014) Revisiting the loess/palaeosol sequence in Paks, Hungary: A post-IR IRSL based chronology for the ‘Young Loess Series’. *Quat Int* **319**: 88-98.
(24) Adamiec G, Aitken M (1998) Dose-rate conversion factors: update. *Ancient TL* **16**: 37-50.
(25) Prescott JR, Hutton JT (1994) Cosmic ray contributions to dose rates for luminescence and ESR dating: Large depths and long-term variations. *Radiation Measurements* **23**: 497-500.
(26) Rees-Jones J (1995) Dating young sediments using fine grained quartz. *Ancient TL* **13**: 9-1.
(27) Rees-Jones J, Tite MS (1997) Optical dating results for British archaeological sediments. *Archaeometry* **36**: 177-187.
(28) Ambrose SH (1990) Preparation and characterization of bone and tooth collagen for isotopic analysis. Journal of Archaeological Science, **17** (4): 431-451.
(29) Weber, A. W., McKenzie, H.G., Beukens, R., Goriumova, O.I. 2005.Evaluation of radiocarbon dates from the Middle-Holocene hunter-gatherer cemetery Khuzhir-Nuge XIV, Lake Baikal, Siberia. *J Archaeol Sci* **32**:1481-1500.

(30) Hublin JJ, Talamo S, Julien M, David F, Connet N, Bodu P, Vandermeersch B, Richards M.P. (2012) Radiocarbon dates from the Grotte du Renne and Saint-Césaire support a Neandertal origin for the Châtelperronian *Proc Natl Acad Sci USA* **109** (46): 18743-18748.
(31) Klinken GVJ (1999) Bone collagen quality indicators for palaeodietary and radiocarbon measurements. *J Archaeol Sci* **26**: 687-695.
(32) Talamo S, Richards MP (2011) Comparison of bone pretreatment methods for AMS dating of samples >30, 000 BP. *Radiocarbon* **53**: 443-449.
(33) Longin R (1971) New method of collagen extraction for radiocarbon dating. *Nature* **230**: 241-242.
(34) Brown TA, Nelson DE, Vogel JS, Southon JR (1988) Improved collagen extraction by modified Longin method. *Radiocarbon* **30**: 171-177.
(35) Brock F, Ramsey CB, Higham T (2007) Quality assurance of ultrafiltered bone dating. *Radiocarbon* **49**: 187-192.
(36) Kromer B, Lindauer S, Synal HA, Wacker L (2013) MAMS – a new AMS facility at the Curt-Engelhorn-Centre for Achaeometry, Mannheim, Germany. *Nuclear Instruments and Methods in Physics Research Section B: Beam Interactions with Materials and Atoms* **29**: 11-13.
(37) Bronk Ramsey, C (2009) Bayesian analysis of radiocarbon dates. *Radiocarbon* **51**(1):337–360.
(38) Bronk Ramsey, C (2009) Dealing with outliers and offsets in radiocarbon dating. *Radiocarbon* **51**(3): 1023–1045.
(39) Bertran P, Texier J-P (1995) Fabric Analysis: Application to Palaeolithic Sites. *J Archaeol Sci* **22**: 521-535.
(40) Lenoble A, Bertran P (2004) Fabric of Palaeolithic levels: Methods and implications for site formation processes. *J Archaeol Sci* **31**: 457-469.
(41) Benn D (1994) Fabric shape and the interpretation of sedimentary fabric data. *Journal of Sedimentary Research* **64**: 910-915.
(42) McPherron SP 2005 Artifact orientations and site formation processes from total station proveniences. *J Archaeol Sci* **32** (7): 1003-1014.
(43) Ringrose TJ, Benn DI (1997) Confidence regions for fabric shape diagrams. *J Struct Geol* **19**: 1527-1536.
(44) McPherron, SP (2018) Additional statistical and graphical methods for analyzing site formation processes using artefact orientations. *PLoS ONE* **13**(1): e0190195.
(45) R Core Team (2018) R: A language and environment for statistical computing. R Foundation for Statistical Computing, Vienna, Austria. URL https://www.R-project.org/
(46) Slavinsky VS, Rybin EP, Belousova NE, Fedorchenko AY, Khatsenovich AM, Anoikin AA, (2017) Specific technique of Core platform preparation in the initial upper Palaeolithic of South Siberia and Central Asia. *Stratum plus* **17** (1): 221–244.

(47) Zwyns N, Lbova LV (2018) The Initial Upper Paleolithic of Kamenka site, Zabaikal region (Siberia): A closer look at the blade technology. *Archaeol Res Asia* (in press).
(48) Pelegrin J (1995) *Technologie lithique: le Chatelperronien de Roc-de-Combe (Lot) et*
de La Cote (Dordogne). CNRS edition, Paris.
(49) Boëda, E., 1990. De la surface au volume, analyse des conceptions, des débitages Levallois et laminaire. Paléolithique moyen et Paléolithique supérieur ancien en Europe. Memoires du Musée de Prehistoire d'ile de France 3, pp. 63 – 68
(50) Roussel, M., Soressi, M., Hublin, J.-J. 2016. The Châtelperronian conundrum: Blade and bladelet lithic technologies from Quinçay, France. Journal of Human Evolution, 13-32.
(51) Zwyns N (2012) *Laminar technology and the onset of Upper Palaeolithic in the Altai (Siberia)* (Leiden Univ Press, Leiden, The Netherlands).
(52) Zwyns N, Rybin EP, Hublin JJ, Derevianko AP (2012) Burin-core technology & laminar reduction sequence in the initial Upper Palaeolithic from Kara-Bom (Gorny-Altai, Siberia). *Quat Int* **259**: 33–47.
(53) Rybin EP (2014) Tools, beads, and migrations: Specific cultural traits in the Initial Upper Palaeolithic of Southern Siberia and Central Asia. *Quat Int* **347**: 39–52.
(54) Kuhn SL, Zwyns N (2014) Rethinking the Initial Upper Palaeolithic. *Quat Int* **347**:29–38.
(55) Škrdla P (2013) The Bohunician in Moravia and adjoining regions. *Archaeol Ethnol Anthropol Eurasia* **41**(3):2–13.
(56) Derevianko AP (2011) *The Upper Palaeolithic in Africa and Eurasia and the Origin of Anatomically Modern Humans*. Institute of Archaeology and Ethnography, Siberian Branch, Russian Academy of Sciences, Novosibirsk.
(57) Goebel T, Aksenov M (1995) Accelerator radiocarbon dating of the Initial Upper Palaeolithic in Southeast Siberia. *Antiquity* **69**(263):349–57.
(58) Lbova L (2008) Chronology and paleoecology of the Early Upper Palaeolithic in the Transbaikal region (Siberia). *Eurasian Prehistory* **5**: 109-114.
(59) Orlova L, Kuzmin Y, Lbova L (2005) *Palaeolithic cultures of the Transbaikal and Mongolia (New facts, methods and hypotheses)*. Institute of Archaeology and Ethnography, Siberian Branch, Russian Academy of Sciences Press, Novosibirsk, 2005) (in Russian).
(60) Tashak V (2009) Laminar Industries from Transbaikal. *Stratum plus* **1**: 79-93 (2009) (in Russian).
(61) Derevianko AP, Rybin EP, Gladyshev SA, Gunchinsuren B, Tsybankov AA, Olsen JW (2013) Early upper paleolithic stone tool technologies of northern mongolia: The case of tolbor-4 and tolbor-15. *Archaeol Ethnol Anthropol* **41**(4): 21-37.

(62) Brantingham, PJ, Gao, X, Madsen, DB, Bettinger, RL, Elston, RG. (2004). The Initial Upper Palaeolithic at Shuidonggou, northwestern China. *The Early Upper Palaeolithic Beyond Western Europe*. eds Brantingham PJ, Kuhn SL, Kerry KW (Los Angeles: University of California Press) pp 233–41.
(63) Peng F, Wang H, Gao X (2014) Blade production of Shuidonggou locality 1 (northwest China): a technological perspective. *Quat Int* **347**: 12–20.
(64) Morgan C, Barton L, Xing G, Mingjie Y, Bettinger R, Gao X (2014) Re-Dating Shuidonggou Locality 1 and Implications for the Initial Upper Palaeolithic in East Asia. *Radiocarbon* **56**:165-179.
(65) Li F, Kuhn SL, Gao X, Chen FY (2013) Re-examination of the dates of large blade technology in China: a comparison of Shuidonggou Locality 1 and Locality 2. *J Hum Evol* **64**:161-168.
(66) Lbova L (2011) Evidence of Modern Human Behavior in the Baikal Zone during the Early Upper Palaeolithic Period. *Bulletin of the Indo-Pacific Prehistory Association* **30**: 9–13.
(67) Kuzmin YV, Orlova LA (1998) Radiocarbon Chronology of the Siberian Palaeolithic. *J World Prehist* **12** (1): 1-53.
(68) Derevianko AP, Markin SV, Shunkov MV (2013) The Sibiryachikha Facies of the Middle Palaeolithic of the Altai. *Archaeol Ethnol Anthropol Eurasia* **41**(1): 89–103.
(69) Derevianko AP, Markin SV, Zykin VS, Zykina VS, Zazhigin VS, Sizikova AO, Solotchina EP, Smolyaninova LG, Antipov AS (2013) Chagyrskaya Cave: A Middle Palaeolithic site in the Altai. Archaeology, *Archaeol Ethnol Anthropol* **41**: 2-27.
(70) Kuzmin, Y. V. (2004) Origin of Upper Palaeolithic in Siberia: Geoarchaeological Perspective *The Early Upper Palaeolithic Beyond Western Europe*. eds Brantingham PJ, Kuhn SL, Kerry KW (Los Angeles: University of California Press) pp.196-206.
(71) Viola, TB *New Hominid Remains from Central Asia and Siberia: the Easternmost Neanderthals?* (PhD Thesis, University of Vienna, Vienna, 2009).
(72) Viola TB, Markin SV, Zenin A, Shunkov MV, Derevianko AP (2011) Late Pleistocene hominins from the Altai mountains, Russia. eds Derevianko AP, Shunkov MV *Characteristic features of the Middle to Upper Palaeolithic Transition in Eurasia* pp. 207-213 (Publishing Department of the Inst. f. Archaeology and Ethnography of the SB RAS, Novosibirsk, 2011).
(73) Buckley M, Collins M, Thomas-Oates J, Wilson JC (2009) Species identification by analysis of bone collagen using matrix-assisted laser desorption/ionisation time-of-flight mass spectrometry. *Rapid Communications in Mass Spectrometry* **23**: 3843–3854.

(74) Welker F, Hadjinjak M, Talamo S, Jaouen K, Dannemann M, David F, Julien M, Meyer M, Kelso J, Barnes I, Brace S, Kamminga P, Fischer R, Kessler BM, Stewart JR, Paabo S, Collins MJ, Hublin JJ (2016) Palaeoproteomic evidence identifies archaic hominins associated with the Châtelperronian at the Grotte du Renne. *Proc. Natl Acad. Sci. USA* **113**:1162–1167.
